# Supplementary material for: Favipiravir, lopinavir-ritonavir, or combination therapy (FLARE): A randomised, double-blind, 2 × 2 factorial placebo-controlled trial of early antiviral therapy in COVID-19
Source: PLoS Med. 2022 Oct 19;19(10):e1004120. doi: 10.1371/journal.pmed.1004120 (PMC9629589; doi:10.1371/journal.pmed.1004120)
Supplement: S1 Appendix — (DOCX) [file pmed.1004120.s001.docx]

**S1 Appendix – FLARE Investigators**

**Trial Steering Committee:**

Kristina Nadrah (Chair), Robert C Read, Elizabeth Allen, Mahdia Sait

**Independent Data Monitoring Committee:**

Stephen Owens (Chair), David Chadwick, April Slee, Andrew Ustianowski

**UCL Comprehensive Clinical Trials Unit:**

Krishneya Anojan, Gemma Jones, Nazma Begum-Ali, Natasha Majid

**Royal Free Hospital Clinical Trials team:**

Rachel Ochiel, Debbie Falconer, Stella O’Connor, Karl Salazar, Tung Le, Francesca Gowing, Ivy Wanjiku Dakouri, Tanaka Ngcozana, Sandra Lopez Garces, Karima Oduka, Daniel Jones, Eva Torok-Pollok

**University College London Hospital Clinical Trials team:**

Michelle Berkeley, Esther King, Kimberlee Gunn

**Great Ormond Street Hospital laboratories:**

Francis Yongblah, Mabel Csatari, Kimberly Gilmour

**Royal Free Hospital and UCL (Royal Free campus) laboratories:**

Naseem Ahmed, Janki Kavi, Nimesha Patel, Hatim Ebrahim

**University of Birmingham laboratories:**

Alex Richter, Adrian Shields
