# Supplementary material for: Favipiravir, lopinavir-ritonavir, or combination therapy (FLARE): A randomised, double-blind, 2 × 2 factorial placebo-controlled trial of early antiviral therapy in COVID-19
Source: PLoS Med. 2022 Oct 19;19(10):e1004120. doi: 10.1371/journal.pmed.1004120 (PMC9629589; doi:10.1371/journal.pmed.1004120)
Supplement: S2 Appendix — (DOCX) [file pmed.1004120.s002.docx]

**S2 Appendix**

**Sample size simulation code in R**

## vectors to collect data

rm(list = ls())

record_trtA_signif <- c()

record_trtB_signif <- c()

record_trtAB_signif <- c()

record_trtAB <- c()

record_trtA <- c()

record_trtB <- c()

## assumptions / data generating mechanism

n.simul=10000

ss_arm = 54

sd <- 1.3

effect_A <- 0.9

effect_B <- 0.9

interaction <- 1

effect_AB <- effect_A + effect_B + interaction

## simulations

for (i in 1:n.simul){

placebo <- rnorm(n=ss_arm,mean=0,sd=sd)

arm_A <- rnorm(n=ss_arm,mean=effect_A,sd=sd)

arm_B <- rnorm(n=ss_arm,mean=effect_B,sd=sd)

arm_AB <- rnorm(n=ss_arm,mean=effect_AB,sd=sd)

y <- c(placebo,arm_A,arm_B,arm_AB)

## analysis as factorial

A <-c(rep(0,ss_arm),rep(1,ss_arm),rep(0,ss_arm),rep(1,ss_arm))

B <-c(rep(0,ss_arm),rep(0,ss_arm),rep(1,ss_arm),rep(1,ss_arm))

AB <-c(rep(0,ss_arm),rep(0,ss_arm),rep(0,ss_arm),rep(1,ss_arm))

dataset <- data.frame(y=y,A=A, B=B, AB=AB)

fit<- lm(y ~ A + B + AB)

summary(fit)

table(predict(fit))

## treatment effects

trtA_signif <- ifelse((summary(fit)$coefficients[2,4]<0.025)&&(summary(fit)$coefficients[2,1]>0),1,0) # 2.5 pct

trtB_signif <- ifelse((summary(fit)$coefficients[3,4]<0.025)&&(summary(fit)$coefficients[3,1]>0),1,0) # 2.5 pct

trtAB_signif <- ifelse((summary(fit)$coefficients[4,4]<0.05)&&(summary(fit)$coefficients[4,1]>0),1,0) # 5 pct

record_trtA_signif <- c(record_trtA_signif, trtA_signif)

record_trtB_signif <- c(record_trtB_signif, trtB_signif)

record_trtAB_signif <- c(record_trtAB_signif, trtAB_signif)

record_trtAB <- c(record_trtAB,summary(fit)$coefficients[4,1])

record_trtA <- c(record_trtA,summary(fit)$coefficients[2,1])

record_trtB <- c(record_trtB,summary(fit)$coefficients[3,1])

}

## results

power_A<-sum(record_trtA_signif)/length(record_trtA_signif)

power_B<-sum(record_trtB_signif)/length(record_trtB_signif)

power_AB<-sum(record_trtAB_signif)/length(record_trtAB_signif)

par(mfrow=c(2,2))

hist(record_trtAB,main="interaction")

hist(record_trtB,main="trt B")

hist(record_trtA,main="trt A")

power_A

power_B

power_AB
