# Supplementary material for: Favipiravir, lopinavir-ritonavir, or combination therapy (FLARE): A randomised, double-blind, 2 × 2 factorial placebo-controlled trial of early antiviral therapy in COVID-19
Source: PLoS Med. 2022 Oct 19;19(10):e1004120. doi: 10.1371/journal.pmed.1004120 (PMC9629589; doi:10.1371/journal.pmed.1004120)
Supplement: S1 Table — (DOCX) [file pmed.1004120.s003.docx]

**S1 Table. Summary statistics of viral load by treatment.**

| **Viral load (log10), mean (SD)** | **Favipiravir+LPV/r (N=61)** | | | **Favipiravir+Placebo (N=59)** | | | **LPV/r+Placebo (N=60)** | | | **Placebo (N=60)** | | |
| --- | --- | --- | --- | --- | --- | --- | --- | --- | --- | --- | --- | --- |
|  | Day 1 | Day 5 | Change | Day 1 | Day 5 | Change | Day 1 | Day 5 | Change | Day 1 | Day 5 | Change |
| ITT | 4.5 (1.6) | 2.6 (2.1) | -1.7 (1.9) | 4.1 (1.6) | 1.9 (2.0) | -2.1 (2.0) | 4.3 (1.6) | 2.5 (2.0) | -1.8 (1.6) | 4.3 (2.0) | 2.7 (2.2) | -1.6 (1.8) |
| mITT | 4.4 (1.5) | 2.6 (2.1) | -1.8 (1.9) | 4.4 (1.0) | 2.1 (2.0) | -2.3 (2.0) | 4.6 (1.1) | 2.7 (2.0) | -1.9 ( 1.55) | 4.8 (1.4) | 3.0 (2.1) | -1.8 (1.8) |

**LPV/r: lopinavir-ritonavir, SD: standard deviation, ITT: intention to treat, mITT: modified intention to treat**
