# Supplementary material for: Favipiravir, lopinavir-ritonavir, or combination therapy (FLARE): A randomised, double-blind, 2 × 2 factorial placebo-controlled trial of early antiviral therapy in COVID-19
Source: PLoS Med. 2022 Oct 19;19(10):e1004120. doi: 10.1371/journal.pmed.1004120 (PMC9629589; doi:10.1371/journal.pmed.1004120)
Supplement: S2 Table — (DOCX) [file pmed.1004120.s004.docx]

**S2 Table. Summary of adverse events.**

|  | **Favipiravir+LPV/r**  **(N=61)** | **Favipiravir+Placebo**  **(N=59)** | **LPV/r+Placebo**  **(N=60)** | **Placebo**  **(N=60)** | **Total  (N=240)** |
| --- | --- | --- | --- | --- | --- |
| **Number of Patients reporting at least 1 AE; N (%)** | 55 (90.1) | 38 (64.4) | 59 (98.3) | 39 (65.0) | 191 (80.0) |
| **Patients with at least one related event** | 53 (87.9) | 27 (45.8) | 56 (93.3) | 21 (35.0) | 157 (65.4) |
| **Number of AEs** | 159 | 92 | 175 | 92 | 518 |
| **Related events** | 108 (67.9) | 44 (47.3) | 116 (65.9) | 27 (29.3) | 295 (56.7) |
|  |  |  |  |  |  |
| **AE Event [# events]** | | | | | |
| Diarrhoea | 41 | 8 | 47 | 10 | 106 |
| Nausea | 16 | 13 | 28 | 6 | 63 |
| Dyspnea | 5 | 6 | 7 | 6 | 24 |
| Headache | 6 | 7 | 4 | 6 | 23 |
| Anosmia | 5 | 3 | 9 | 5 | 22 |
| Fatigue | 4 | 4 | 7 | 7 | 22 |
| Vomiting | 8 | 1 | 6 | 2 | 17 |
| Cough | 2 | 5 | 4 | 5 | 16 |
| Dysgeusia | 3 | 4 | 6 | 3 | 16 |
| Abdominal pain | 2 | 3 | 2 | 5 | 12 |
| Anorexia | 2 | 1 | 7 | 2 | 12 |
| Dizziness | 4 | 1 | 6 | 0 | 11 |
| Alanine aminotransferase increased | 6 | 1 | 1 | 1 | 9 |
| Myalgia | 3 | 2 | 1 | 3 | 9 |
| Rash maculo-papular | 2 | 4 | 1 | 2 | 9 |
| Aspartate aminotransferase increased | 4 | 0 | 1 | 2 | 7 |
| Nasal congestion | 1 | 3 | 1 | 2 | 7 |
| Non-cardiac chest pain | 4 | 0 | 1 | 2 | 7 |
| Hyperuricemia | 0 | 2 | 0 | 0 | 2 |

**LPV/r: lopinavir-ritonavir, AE: adverse event**
