# Supplementary material for: Favipiravir, lopinavir-ritonavir, or combination therapy (FLARE): A randomised, double-blind, 2 × 2 factorial placebo-controlled trial of early antiviral therapy in COVID-19
Source: PLoS Med. 2022 Oct 19;19(10):e1004120. doi: 10.1371/journal.pmed.1004120 (PMC9629589; doi:10.1371/journal.pmed.1004120)
Supplement: S3 Table — (DOCX) [file pmed.1004120.s005.docx]

**S3 Table. Serum liver function tests and uric acid at Day 1 and Day 7.**

|  | **Favipiravir+LPV/r  (N=61)** | | **Favipiravir+Placebo  (N=59)** | | **LPV/r+Placebo  (N=60)** | | **Placebo  (N=60)** | | **Total  (N=240)** | |
| --- | --- | --- | --- | --- | --- | --- | --- | --- | --- | --- |
|  | median  (IQR) | Outside normal range  N (%) | median  (IQR) | Outside normal range  N (%) | median  (IQR) | Outside normal range  N (%) | median  (IQR) | Outside normal range  N (%) | median  (IQR) | Outside normal range  N (%) |
| **ALT (IU/L)** | | | | | | | | | | |
| Day 1 | 27.0 (19.0-41.0) | 18 (30.0) | 28.0 (20.0-45.0) | 15 (25.9) | 24.0 (15.0-36.5) | 10 (16.9) | 24.5 (15.0-38.5) | 15 (25.0) | 26.0 (16.0-41.0) | 58 (24.5) |
| Day 7 | 27.0 (18.0-37.0) | 15 (25.0) | 35.5 (24.5-50.5) | 22 (37.9) | 21.0 (15.0-28.0) | 4 (6.8) | 22.5 (17.5-36.0) | 13 (21.7) | 26.0 (18.0-38.5) | 54 (22.8) |
| Change | -2.0 (-12.0- 6.0) |  | 1.0 (-3.0-12.0) |  | -2.0 (-8.0- 1.0) |  | 0.0 (-4.5- 5.0) |  | -1.0 (-7.0- 5.0) |  |
| **AST (IU/L)** | | | | | | | | | | |
| Day 1 | 32.0 (28.0-41.0) | 18 (30.0) | 34.0 (28.0-43.0) | 19 (32.8) | 31.0 (27.0-36.5) | 12 (20.3) | 30.5 (26.0-36.5) | 16 (26.7) | 32.0 (27.0-38.0) | 65 (27.4) |
| Day 7 | 28.0 (25.0-34.0) | 13 (21.7) | 32.5 (28.0-39.5) | 15 (25.9) | 28.0 (25.0-31.0) | 3 (5.1) | 28.0 (24.0-32.5) | 9 (15.0) | 29.0 (25.0-34.0) | 40 (16.9) |
| Change | -3.0 (-8.0- 1.0) |  | -2.0 (-6.0- 3.0) |  | -2.0 (-6.0- 0.0) |  | -1.5 (-5.0- 1.5) |  | -2.0 (-7.0- 2.0) |  |
| **ALP (IU/L)** | | | | | | | | | | |
| Day 1 | 60.0 (53.0-74.0) | 1 (1.7) | 60.5 (54.0-70.0) | 0 (0.0) | 57.5 (49.0-72.5) | 0 (0.0) | 60.5 (52.0-72.5) | 1 (1.7) | 60.0 (52.0-72.0) | 2 (0.8) |
| Day 7 | 67.0 (57.0-83.0) | 2 (3.3) | 66.0 (58.5-77.0) | 1 (1.7) | 60.0 (51.0-76.0) | 1 (1.7) | 66.0 (54.5-77.0) | 1 (1.7) | 65.0 (55.0-78.0) | 5 (2.1) |
| Change | 4.0 (1.0-12.0) |  | 6.0 (2.0-13.0) |  | 2.0 (-2.0- 6.0) |  | 1.5 (-1.0- 8.0) |  | 4.0 (0.0-10.0) |  |
| **Bilirubin (μmol/L)** | | | | | | | | | | |
| Day 1 | 5.0 (4.0- 8.0) | 1 (1.7) | 6.0 (4.0- 8.0) | 1 (1.7) | 6.0 (3.0- 7.0) | 0 (0.0) | 7.0 (4.0- 9.0) | 0 (0.0) | 6.0 (4.0- 8.0) | 2 (0.8) |
| Day 7 | 10.0 (6.0-14.0) | 2 (3.3) | 6.0 (4.0- 9.0) | 1 (1.7) | 8.5 (6.0-13.0) | 4 (6.8) | 7.0 (5.0-10.0) | 1 (1.7) | 8.0 (5.0-12.0) | 8 (3.4) |
| Change | 4.0 (1.0- 8.0) |  | 1.0 (-1.0- 3.0) |  | 4.5 (0.0- 8.5) |  | 2.0 (-2.0- 3.0) |  | 2.0 (0.0- 5.0) |  |
| **Uric acid (μmol/L)** | | | | | | | | | | |
| Day 1 | 275.0 (209.0-336.0) | 7 (11.5) | 253.0 (216.0-315.0) | 5 (8.5) | 256.5 (196.0-297.5) | 6 (10.0) | 285.0 (210.5-327.0) | 5 (8.3) | 265.0 (209.0-320.0) | 23 (9.6) |
| Day 7 | 369.5 (299.0-441.0) | 18 (29.5) | 422.5 (349.5-498.0) | 22 (37.3) | 258.5 (203.5-316.0) | 5 (8.3) | 275.5 (238.5-346.0) | 2 (3.3) | 329.0 (251.0-401.0) | 47 (19.6) |
| Day 14 | 306.0 (265.0 - 400.0) | 2 (8.0) | 329.0 (287.0 - 354.0) | 1 (3.7) | 288.5 (247.5 - 331.5) | 1 (5.0) | 310.0 (237.0 - 353.0) | 2 (9.1) | 303.5 (250.0 - 253.0) | 6 (6.4) |
| Change | -2.0 (-12.0-6.0) |  | 1.0 (-3.0-12.0) |  | -2.0 (-8.0- 1.0) |  | 0.0 (-4.5- 5.0) |  | -1.0 (-7.0- 5.0) |  |

**LPV-r: lopinavir/ritonavir, IQR: interquartile range, ALT: alanine aminotransferase, AST: aspartate aminotransferase, ALP: alkaline phosphatase, IU: international units, μmol: micromoles**
