# Supplementary material for: Favipiravir, lopinavir-ritonavir, or combination therapy (FLARE): A randomised, double-blind, 2 × 2 factorial placebo-controlled trial of early antiviral therapy in COVID-19
Source: PLoS Med. 2022 Oct 19;19(10):e1004120. doi: 10.1371/journal.pmed.1004120 (PMC9629589; doi:10.1371/journal.pmed.1004120)
Supplement: S4 Table — (DOCX) [file pmed.1004120.s006.docx]

**S4 Table. Baseline characteristics of the cohort with pharmacokinetic measurements.**

| **Characteristics of Pk group at screening** |  | **Favipiravir+LPV/r**  **(N=28)** | **Favipiravir+Placebo**  **(N=31)** |
| --- | --- | --- | --- |
| Age (years) | mean (sd) | 39.4 (13.4) | 40.9 (11.7) |
| Gender | N (%) |  |  |
| Male |  | 16 (57.1) | 17 (54.8) |
| Female |  | 12 (42.9) | 14 (45.2) |
| Ethnicity | N (%) |  |  |
| Caucasian |  | 23 (82.1) | 27 (87.1) |
| Other |  | 5 (17.9) | 4 (12.9) |
| BMI (kg/m^2^) | N (%) |  |  |
| <30 |  | 23 (82.1) | 49 (83.1) |
| ≥30 |  | 5 (17.9) | 5 (16.1) |

**LPV/r: lopinavir-ritonavir; Pk: pharmacokinetics.**
