# Supplementary material for: Favipiravir, lopinavir-ritonavir, or combination therapy (FLARE): A randomised, double-blind, 2 × 2 factorial placebo-controlled trial of early antiviral therapy in COVID-19
Source: PLoS Med. 2022 Oct 19;19(10):e1004120. doi: 10.1371/journal.pmed.1004120 (PMC9629589; doi:10.1371/journal.pmed.1004120)
Supplement: S5 Table — (DOCX) [file pmed.1004120.s007.docx]

**S5 Table. Number of patients (i) having fever (temperature>37.8 °C) at Day 5 (ii) hospitalised during the trial (iii) admitted to Intensive care unit.**

|  | **Favipiravir+LPV/r (N=61)** | **Favipiravir+Placebo (N=59)** | **LPV/r+Placebo (N=60)** | **Placebo (N=60)** | **Total  (N=240)** |
| --- | --- | --- | --- | --- | --- |
| (i) Having fever at Day 5 | 2 | 3 | 3 | 3 | 11 |
| (ii) Hospitalised | 1 | 1 | 1 | 0 | 3 |
| (iii) Admitted to Intensive Care Unit | 0 | 1 | 0 | 0 | 1 |
