# Supplementary figures and images for: Favipiravir, lopinavir-ritonavir, or combination therapy (FLARE): A randomised, double-blind, 2 × 2 factorial placebo-controlled trial of early antiviral therapy in COVID-19
Source: PLoS Med. 2022 Oct 19;19(10):e1004120. doi: 10.1371/journal.pmed.1004120 (PMC9629589; doi:10.1371/journal.pmed.1004120)

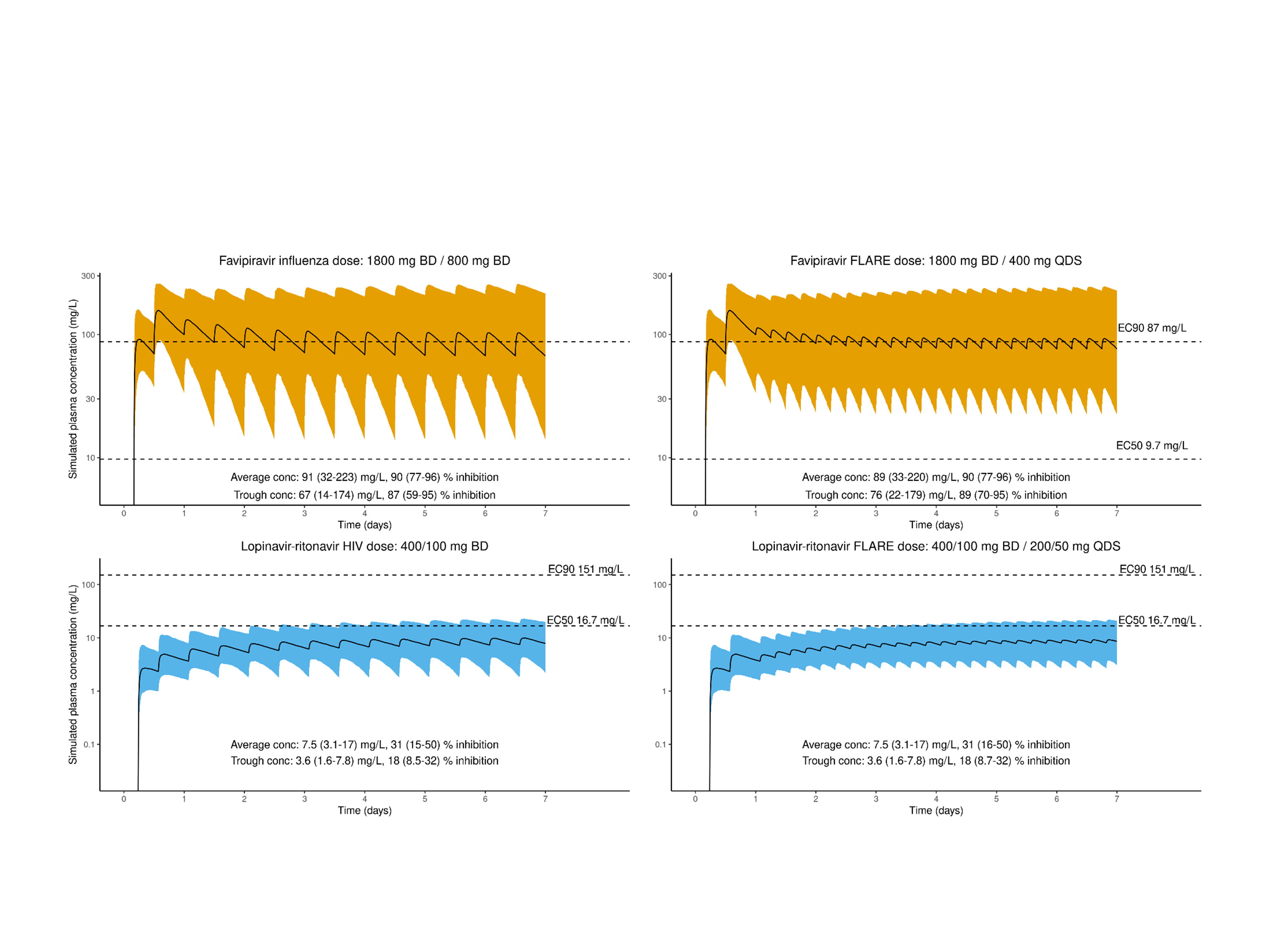

Supplement: S1 Fig — Simulations are presented for a twice daily (BD) dosing regime and four times daily (QDS) dosing regime. (TIF) [file pmed.1004120.s008.tif]

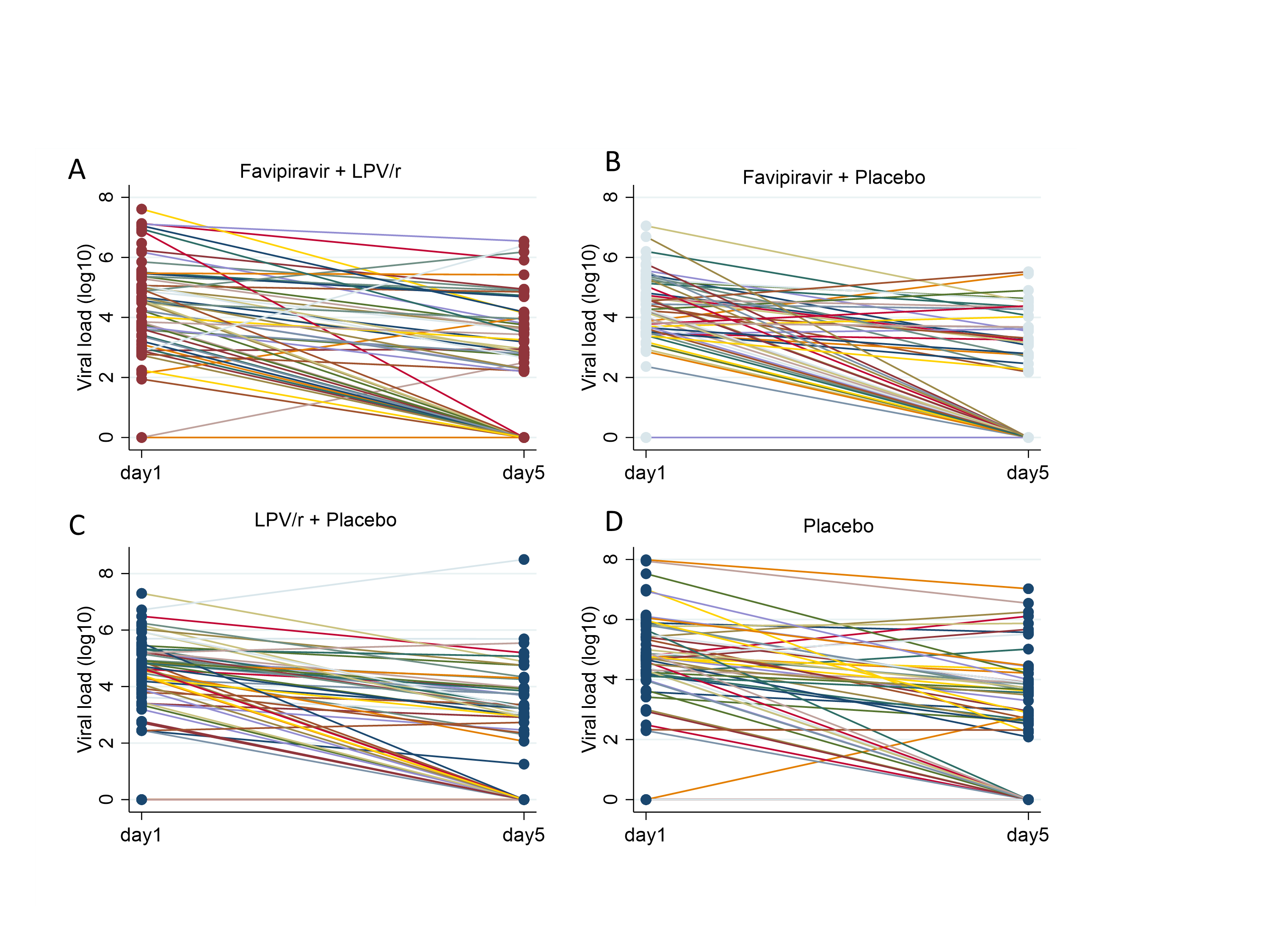

Supplement: S2 Fig — Log10 SARS-CoV-2 viral load at baseline (Day 1) and Day 5 presented per participant for (A) favipiravir+lopinavir-ritonavir (LPV/r), (B) favipiravir+placebo, (C) LPV/r + placebo and (D) placebo only. (TIF) [file pmed.1004120.s009.tif]

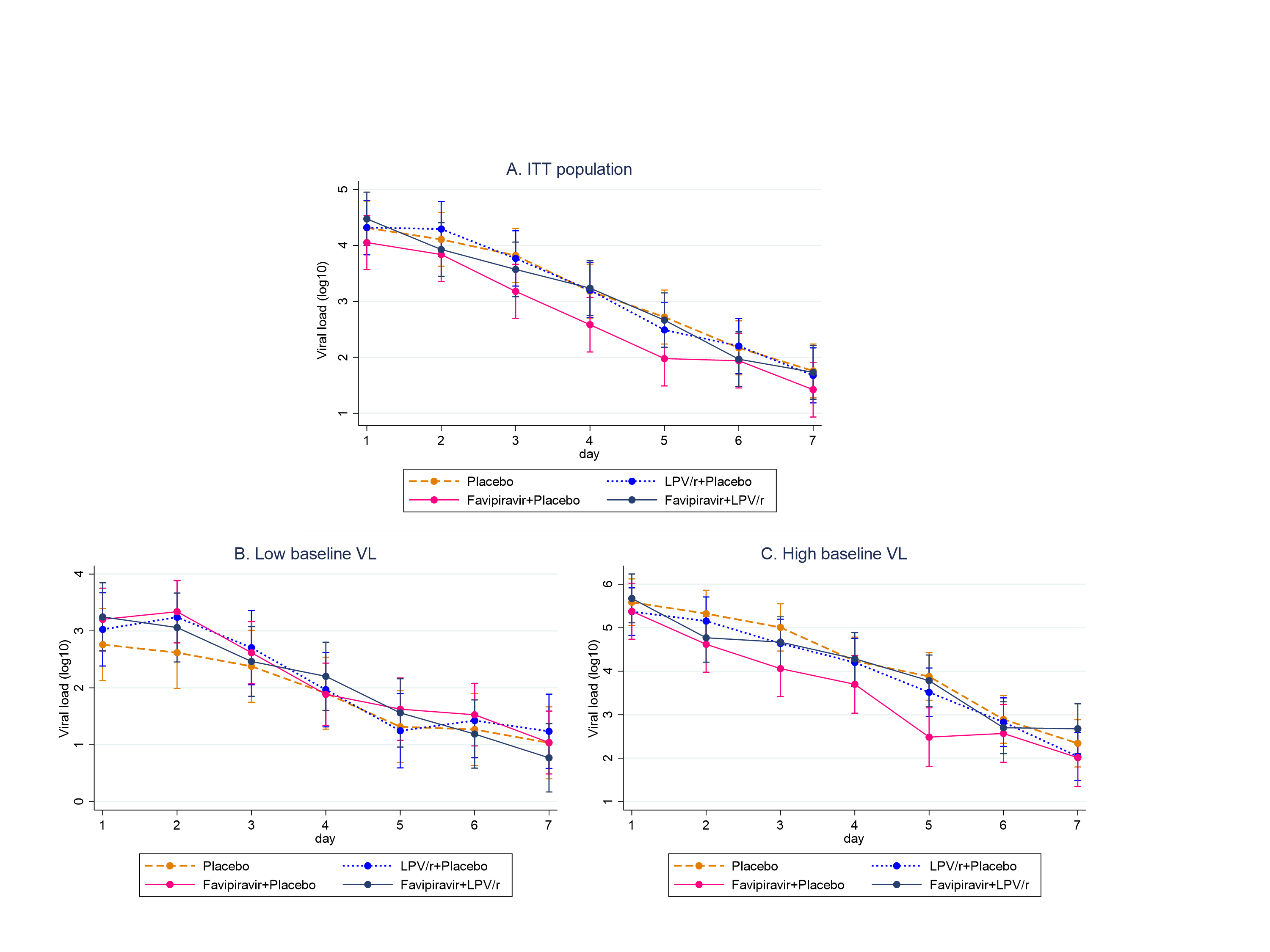

Supplement: S3 Fig — Mean log10 SARS-CoV-2 viral load per treatment arm on each day of treatment in (A) the entire cohort, (B) participants with baseline viral load below or equal to the median level and (C) participants with baseline viral load above the median level. (TIF) [file pmed.1004120.s010.tif]

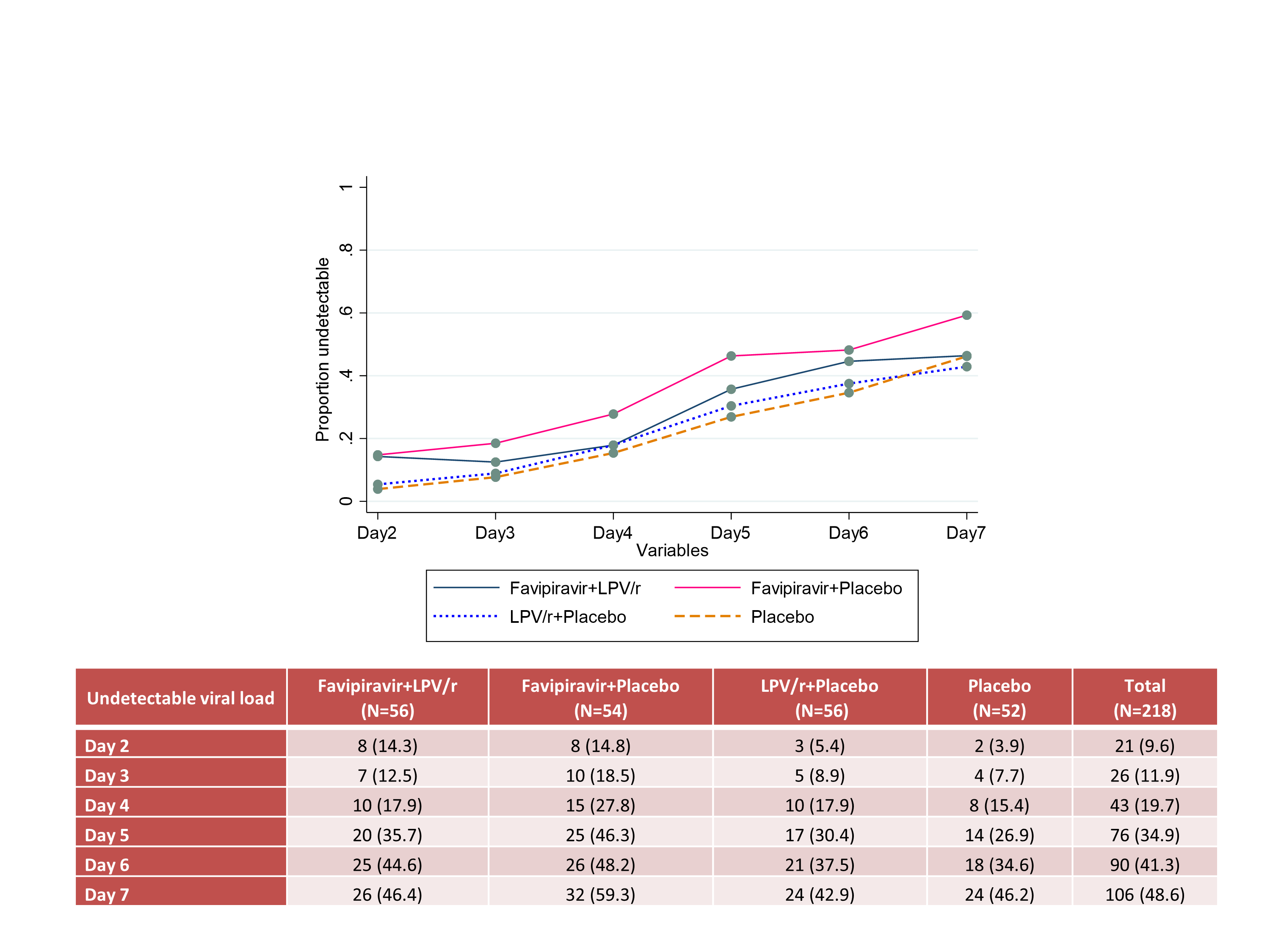

Supplement: S4 Fig — Underlying data are presented in the accompanying table. (TIF) [file pmed.1004120.s011.tif]

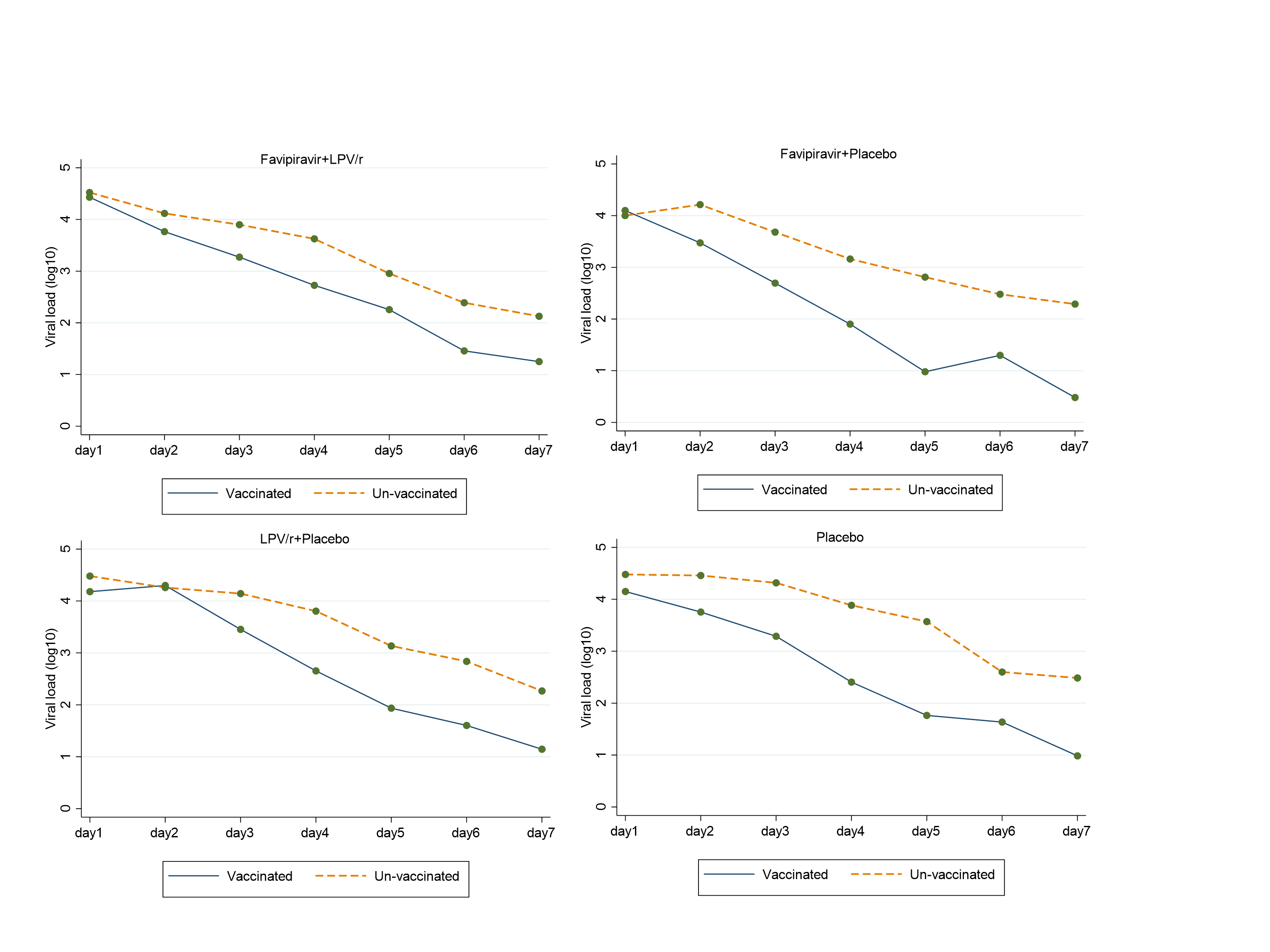

Supplement: S5 Fig — LPV/r, lopinavir-ritonavir. (TIF) [file pmed.1004120.s012.tif]

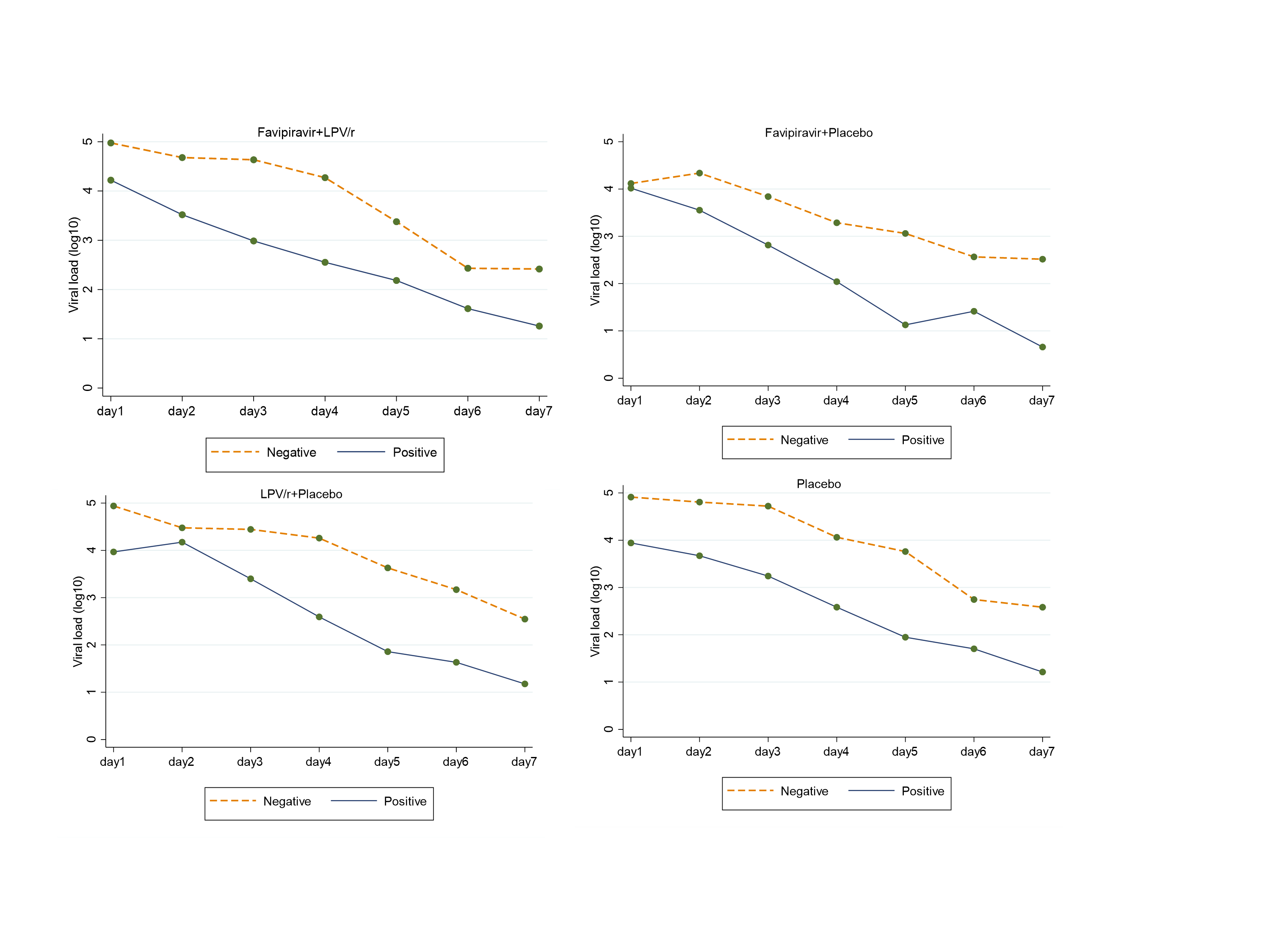

Supplement: S6 Fig — LPV/r, lopinavir-ritonavir. (TIF) [file pmed.1004120.s013.tif]

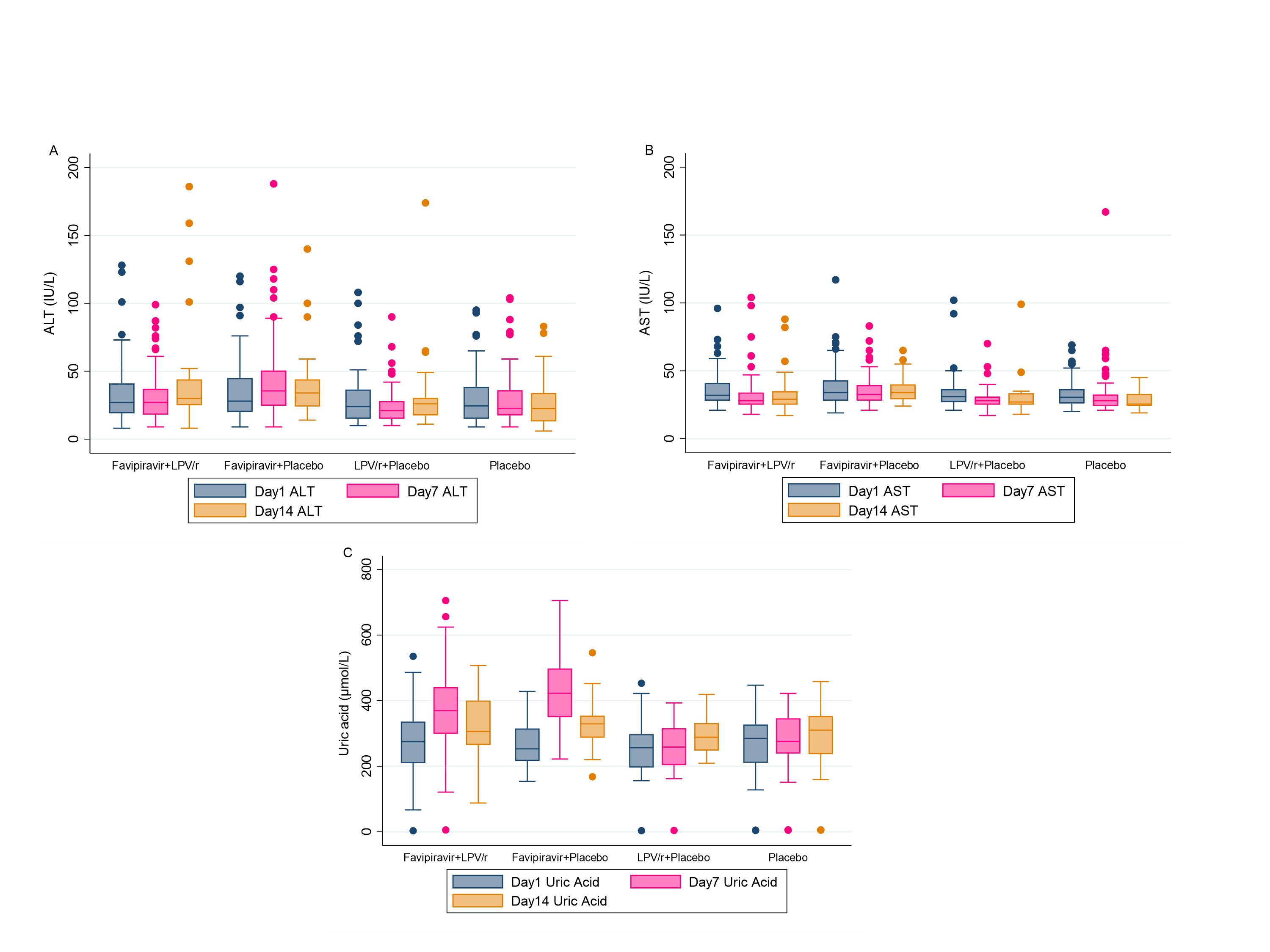

Supplement: S7 Fig — (A) Serum ALT concentration, (B) serum AST concentration and (C) serum uric acid concentration at Day 1, Day 7 and Day 14 according to treatment arm. Blood tests were usually only taken at Day 14 if abnormal at Day 7. Boxes represent IQR and whiskers represent 1.5*IQR. ALT, alanine aminotransferase; AST, aspartate aminotransferase; IQR, interquartile range. (TIF) [file pmed.1004120.s014.tif]
