# Supplementary material for: Favipiravir, lopinavir-ritonavir, or combination therapy (FLARE): A randomised, double-blind, 2 × 2 factorial placebo-controlled trial of early antiviral therapy in COVID-19
Source: PLoS Med. 2022 Oct 19;19(10):e1004120. doi: 10.1371/journal.pmed.1004120 (PMC9629589; doi:10.1371/journal.pmed.1004120)
Supplement: S1 Consort Checklist — (DOC) [file pmed.1004120.s015.doc]

CONSORT 2010 checklist of information to include when reporting a randomised trial*

| Section/Topic | Item No | Checklist item | Reported on page No |
| --- | --- | --- | --- |
| Title and abstract | | | |
|  | 1a | Identification as a randomised trial in the title | 1 (Title) |
|  | 1b | Structured summary of trial design, methods, results, and conclusions (for specific guidance see CONSORT for abstracts) | Abstract |
| Introduction | | | |
| Background and objectives | 2a | Scientific background and explanation of rationale | Introduction paragraphs 1-5 |
|  | 2b | Specific objectives or hypotheses | Introduction paragraph 5 |
| Methods | | | |
| Trial design | 3a | Description of trial design (such as parallel, factorial) including allocation ratio | Materials and Methods paragraph 1 |
|  | 3b | Important changes to methods after trial commencement (such as eligibility criteria), with reasons | Materials and Methods paragraph 2 |
| Participants | 4a | Eligibility criteria for participants | Materials and Methods paragraph 2 |
|  | 4b | Settings and locations where the data were collected | Materials and Methods paragraphs 3-4, 8 and 9 |
| Interventions | 5 | The interventions for each group with sufficient details to allow replication, including how and when they were actually administered | Materials and Methods paragraph 1, 7 and 8 |
| Outcomes | 6a | Completely defined pre-specified primary and secondary outcome measures, including how and when they were assessed | Materials and Methods paragraph 10 |
|  | 6b | Any changes to trial outcomes after the trial commenced, with reasons | Materials and Methods paragraph 11 |
| Sample size | 7a | How sample size was determined | Materials and Methods paragraph 15 |
|  | 7b | When applicable, explanation of any interim analyses and stopping guidelines | N/A |
| Randomisation: |  |  |  |
| Sequence generation | 8a | Method used to generate the random allocation sequence | Materials and Methods paragraph 6 |
|  | 8b | Type of randomisation; details of any restriction (such as blocking and block size) | Materials and Methods paragraph 6 |
| Allocation concealment mechanism | 9 | Mechanism used to implement the random allocation sequence (such as sequentially numbered containers), describing any steps taken to conceal the sequence until interventions were assigned | Materials and Methods paragraph 6 |
| Implementation | 10 | Who generated the random allocation sequence, who enrolled participants, and who assigned participants to interventions | Materials and Methods paragraphs 3, 4, 6 and 7 |
| Blinding | 11a | If done, who was blinded after assignment to interventions (for example, participants, care providers, those assessing outcomes) and how | Materials and Methods paragraph 7 |
|  | 11b | If relevant, description of the similarity of interventions | Materials and Methods paragraph 7 |
| Statistical methods | 12a | Statistical methods used to compare groups for primary and secondary outcomes | Materials and Methods paragraphs 15-17 |
|  | 12b | Methods for additional analyses, such as subgroup analyses and adjusted analyses | Materials and Methods paragraphs 16-17 |
| Results | | | |
| Participant flow (a diagram is strongly recommended) | 13a | For each group, the numbers of participants who were randomly assigned, received intended treatment, and were analysed for the primary outcome | Figure 1 |
|  | 13b | For each group, losses and exclusions after randomisation, together with reasons | Results paragraph 2 and Figure 1 |
| Recruitment | 14a | Dates defining the periods of recruitment and follow-up | Results paragraph 1 |
|  | 14b | Why the trial ended or was stopped | N/A (met recruitment target) |
| Baseline data | 15 | A table showing baseline demographic and clinical characteristics for each group | Table 1 and Table 2 |
| Numbers analysed | 16 | For each group, number of participants (denominator) included in each analysis and whether the analysis was by original assigned groups | Figure 1, all Tables and Supplementary Tables |
| Outcomes and estimation | 17a | For each primary and secondary outcome, results for each group, and the estimated effect size and its precision (such as 95% confidence interval) | Results paragraphs 3-14, Tables 3-4, Supplementary Tables 1 and 5, Figures 2 and 4, Supplementary Figures 2-4 |
|  | 17b | For binary outcomes, presentation of both absolute and relative effect sizes is recommended | Results paragraph 5, Table 4, Supplementary Figure 4 |
| Ancillary analyses | 18 | Results of any other analyses performed, including subgroup analyses and adjusted analyses, distinguishing pre-specified from exploratory | Results paragraphs 4, 6 and 7, Figure 3, Tables 3 and 5, Supplementary Figure 5 |
| Harms | 19 | All important harms or unintended effects in each group (for specific guidance see CONSORT for harms) | Results paragraphs 9-12, Supplementary Tables 2 and 3, Supplementary Figure 6 |
| Discussion | | | |
| Limitations | 20 | Trial limitations, addressing sources of potential bias, imprecision, and, if relevant, multiplicity of analyses | Discussion paragraph 13 |
| Generalisability | 21 | Generalisability (external validity, applicability) of the trial findings | Discussion paragraph 13 |
| Interpretation | 22 | Interpretation consistent with results, balancing benefits and harms, and considering other relevant evidence | Discussion paragraphs 1, 2, 5-12 and 14 |
| Other information | | |  |
| Registration | 23 | Registration number and name of trial registry | Abstract, Materials and Methods paragraph 5 |
| Protocol | 24 | Where the full trial protocol can be accessed, if available | Submitted with manuscript |
| Funding | 25 | Sources of funding and other support (such as supply of drugs), role of funders | Abstract |

*We strongly recommend reading this statement in conjunction with the CONSORT 2010 Explanation and Elaboration for important clarifications on all the items. If relevant, we also recommend reading CONSORT extensions for cluster randomised trials, non-inferiority and equivalence trials, non-pharmacological treatments, herbal interventions, and pragmatic trials. Additional extensions are forthcoming: for those and for up to date references relevant to this checklist, see [www.consort-statement.org](http://www.consort-statement.org/).
