# Supplementary material for: Favipiravir, lopinavir-ritonavir, or combination therapy (FLARE): A randomised, double-blind, 2 × 2 factorial placebo-controlled trial of early antiviral therapy in COVID-19
Source: PLoS Med. 2022 Oct 19;19(10):e1004120. doi: 10.1371/journal.pmed.1004120 (PMC9629589; doi:10.1371/journal.pmed.1004120)
Supplement: S1 Statistical Analysis — (DOCX) [file pmed.1004120.s016.docx]

FLARE

**Favipiravir +/- Lopinavir: A RCT of Early antivirals**

Favipiravir, lopinavir/ritonavir or combination therapy: a randomised, double blind, 2x2 factorial placebo-controlled trial of early antiviral therapy in COVID-19

UCL CTU ID: CTU/2020/354

UCL SPONSOR ID: 132084

Protocol version: 6.0 dated 08 Jun 2021

European Clinical Trials Database Number (EudraCT):  2020-002106-68

**ClinicalTrials.gov Identifier: NCT04499677**

**Research Ethics Committee Number: 20/WA/0210**

**Statistical Analysis Plan (SAP)**

**Version 1.0, 04 November 2021**

| **Approved by:** |  | **Signature** |  | **Date** |
| --- | --- | --- | --- | --- |
| Dr David Lowe  Chief Investigator |  |  |  |  |
| Dr Hakim-Moulay Dehbi  Oversight Statistician CCTU |  |  |  |  |
|  |  |  |  |  |
| Prepared by: | Kashfia Chowdhury  Trial Statistician  Email: [k.chowdhury@ucl.ac.uk](mailto:k.chowdhury@ucl.ac.uk)  Tel: 020 3108 3942 | |  | |

**Table of contents**

[ABBREVIATIONS AND GLOSSARY 3](#_Toc86933877)

[1 Introduction 4](#_Toc86933878)

[1.1 Background and rationale 4](#_Toc86933879)

[1.2 Objectives 5](#_Toc86933880)

[2 study methods 5](#_Toc86933881)

[2.1 Trial design 5](#_Toc86933882)

[2.2 Randomisation 5](#_Toc86933883)

[2.3 Sample size 5](#_Toc86933884)

[2.4 Framework 6](#_Toc86933885)

[2.5 Statistical interim analyses and stopping guidance 6](#_Toc86933886)

[2.6 Timing of final analysis 6](#_Toc86933887)

[2.7 Timing of outcome assessments 6](#_Toc86933888)

[3 Statistical principles 6](#_Toc86933889)

[3.1 Confidence intervals and p-values 6](#_Toc86933890)

[3.2 Analysis population 7](#_Toc86933891)

[4 Trial population 7](#_Toc86933892)

[4.1 Screening, recruitment, withdrawal/follow-up 7](#_Toc86933893)

[4.2 Eligibility 7](#_Toc86933894)

[4.3 Baseline patient characteristics 7](#_Toc86933895)

[5 Analysis 8](#_Toc86933896)

[5.1 Outcome definitions 8](#_Toc86933897)

[5.1.1 Primary outcome 8](#_Toc86933898)

[5.1.2 Secondary outcomes 8](#_Toc86933899)

[5.1.3 Exploratory outcome 9](#_Toc86933900)

[5.1.4 Rationale and details for outcome measures 9](#_Toc86933901)

[5.2 Analysis methods 9](#_Toc86933902)

[5.2.1 Adjustment factors 10](#_Toc86933903)

[5.2.2 Primary outcome analysis 10](#_Toc86933904)

[5.2.3 Secondary outcome analysis 11](#_Toc86933905)

[5.2.4 Subgroup analysis 13](#_Toc86933906)

[5.2.5 Exploratory outcome analysis 14](#_Toc86933907)

[6 References 15](#_Toc86933908)

[7 revision history 16](#_Toc86933909)

[8 APPENDICES 17](#_Toc86933910)

# ABBREVIATIONS AND GLOSSARY

| Anosmia | Loss of the sense of smell, either total or partial |
| --- | --- |
| Cholangitis | Inflammation of the bile duct system |
| Coryza | Inflammation of the mucous membranes lining the nasal cavity, usually causing a running nose, nasal congestion and loss of smell |
| COVID-19 | Coronavirus disease caused by severe acute respiratory syndrome coronavirus 2 (SARS-CoV-2) |
| Highly effective contraception | For this trial, contraceptive methods that result in a failure rate of < 1% per year, supplemented with a barrier method (preferably male condom). This applies from the time of signing the informed consent form until 7 days after the last trial medication dose intake. |
| MERS-CoV | Middle East respiratory syndrome coronavirus (MERS-CoV) coronavirus causing previous outbreak in 2012 |
| SARS-CoV-1 | Severe acute respiratory syndrome-1 coronavirus causing previous outbreak in 2003 |
| SARS-CoV-2 | Severe acute respiratory syndrome coronavirus 2, causing COVID-19 pandemic in 2020 |
| Steatohepatitis | Type of fatty liver disease, characterised by inflammation of the liver with concurrent fat accumulation in liver |

# Introduction

## Background and rationale

The 2020 pandemic of SARS-CoV-2 causing COVID-19 disease is an unprecedented global emergency. COVID-19 appears to be a disease with an early phase where the virus replicates, coinciding with first presentation of symptoms, followed by a later ‘inflammatory’ phase which results in severe disease in some individuals. It is known from other rapidly progressive infections such as sepsis and influenza that early treatment with antimicrobials is associated with better outcome. The hypothesis is that this holds for COVID-19 and that early antiviral treatment may prevent progression to the later phase of the disease.

The antiviral drug favipiravir is active against a broad range of viruses and has shown promising results in COVID-19 disease in two small Chinese studies^1^. The drug has a well-established and currently available oral formulation with a good safety profile. The results of the early studies^1^ on favipiravir in COVID-19 urgently need to be confirmed or refuted in a high-quality placebo-controlled trial. However, it should also be noted that single agent oral favipiravir may not be sufficient for rapid viral eradication.

Another antiviral with potential activity in COVID-19 is the combined tablet lopinavir/ritonavir (LPV/r). This is a well-established therapy for HIV infection and lopinavir is known to have some antiviral activity against coronaviruses. Although LPV/r did not demonstrate a significant impact in COVID-19 as a single therapy in a recent study^4^ in severely unwell patients, a numerical reduction in mortality (8% versus 13%) and reduced intensive care stay was observed in patients who received the drug early. Favipiravir and LPV/r have different mechanisms of action so given together are likely to act at least additively, and possibly synergistically, providing strong rationale for a trial which examines combination therapy.

Therefore the plan is to conduct a proof-of-principle placebo-controlled clinical trial of favipiravir plus or minus LPV/r in adults. Participants with or without symptomatic COVID-19 or tested positive will be assigned to receive favipiravir plus LPV/r or favipiravir plus LPV/r placebo or favipiravir placebo plus LPV/r or favipiravir placebo plus LPV/r placebo.

## Objectives

FLARE is a multi-centre, phase IIA, randomised, placebo-controlled trial which aims to assess whether early antiviral therapy with either favipiravir + LPV/r, LPV/r or favipiravir is associated with a decrease in viral load compared with placebo, in participants with COVID-19. The primary objective described in this SAP is to compare the difference in the amount of virus (‘viral load’) in the upper respiratory tract after 5 days of therapy.

# study methods

## Trial design

FLARE is a double-blind, 2x2 factorial trial, as shown in table below. Patients will be randomised 1:1:1:1 to receive either favipiravir + LPV/r**,** favipiravir + LPV/r placebo, favipiravir placebo + LPV/r or favipiravir placebo + LPV/r placebo.

|  | | **Factor 1 - Favipiravir** | |
| --- | --- | --- | --- |
|  |  | Active treatment | Placebo |
| **Factor 2 - LPV/r** | Active treatment | Favipiravir + LPV/r | LPV/r alone |
|  | Placebo | Favipiravir alone | Placebo |

## Randomisation

Randomisation will be by minimisation, with the following factors: trial site, age (≤ 55 vs > 55 years old), gender, obesity (BMI <30 vs ≥30), symptomatic or asymptomatic, current smoking status (Yes = current smoker, No = ex-smoker, never smoker), ethnicity (Caucasian, other), previous COVID-19 specific vaccination (Yes/No) and presence or absence of comorbidity (defined as diabetes, hypertension, ischaemic heart disease (including previous myocardial infarction), other heart disease (arrhythmia and valvular heart disease), asthma, COPD, other chronic respiratory disease). The minimisation algorithm will incorporate a random element to maximise balance in the minimisation factors between the randomised groups.

## Sample size

We have conducted extensive power calculation and power simulation exercises for the proposed factorial design. In the proposed design 240 participants (216 evaluable participants, which implies a 10% allowance for attrition and other methodological challenges) will be recruited, so that half of participants receive each of the experimental treatments, one quarter receive both treatments, and one quarter receive neither (with double dummy placebos in place). Both active treatments (main effects) provide 90% power with alpha of 2.5% to find a difference of 0.9 log_10_ viral load. The interaction term is powered (with 80% power and nominal alpha of 5%) to find a difference, either added to or subtracted from the additive effect of each treatment, of 1.0 log_10_ viral load to be statistically significant.

## Framework

FLARE is a superiority trial, the primary purpose of which is to demonstrate that early antiviral therapy with either favipiravir + LPV/r, LPV/r or favipiravir is associated with a decrease in viral load compared with placebo.

## Statistical interim analyses and stopping guidance

There will be no interim analyses. Monitoring of the safety of the trial will be undertaken by an Independent Data Monitoring Committee (IDMC) which will have untrammelled access to the trial data, and whose work is governed by a separate charter.

The IDMC will review unblinded data and make recommendations to the Trial Steering Committee. Further details of the roles and responsibilities of the IDMC, including membership, relationships with other committees, decision making processes, and the timing and frequency of interim analyses, (and description of stopping rules and/or guidelines where applicable) are described in detail in the FLARE IDMC Terms of Reference (ToR).

## Timing of final analysis

It is expected that the last patient’s primary endpoint data visit will be in December 2021. All CRFs for primary endpoint data should be available within 7 days of this visit. Data query and cleaning will be ongoing during the course of the trial, but final cleaning will commence once this last CRF is entered into the database.

The final analysis will start when all data for the primary endpoint is entered into the database and all corresponding queries are resolved.

## Timing of outcome assessments

The timing of outcome assessments is provided in section 6.7 Participant Timeline of the protocol.

# Statistical principles

## Confidence intervals and p-values

All applicable statistical tests will be 2-sided and all p-values from hypothesis testing will be exact. The main treatment effects will be tested at the 2.5% in line with the sample size calculation. All confidence intervals presented will be 95% and two-sided.

## Analysis population

The primary outcome analysis will be conducted following the intention to treat (ITT) principle where all randomised patients are analysed in their allocated group whether or not they receive their randomised treatment.

An ITT analysis will be performed for all secondary outcomes.

There will be no imputation of missing data for any of the study outcomes.

# Trial population

## Screening, recruitment, withdrawal/follow-up

Patients screened but not enrolled in the trial and reasons for exclusions will be reported, and recruitment will be presented by centre and month.

The number of patients who have been withdrawn or were unwilling to continue trial follow-up will be reported by treatment arm.

The throughput of patients from those screened, enrolled, assessed for trial endpoints, and included in the analysis, will be summarised in a CONSORT flowchart.^2^

## Eligibility

Eligibility and inclusion/exclusion criteria are provided in the protocol in section 6.3.

## Baseline patient characteristics

The list of baseline characteristics to be summarised is provided in Appendix A at the end of this document.

Baseline characteristics will be summarised for all patients in the study. Summary measures for the baseline characteristics will be presented as mean and standard deviation for continuous (approximate) normally distributed variables, medians and interquartile ranges for non-normally distributed continuous variables, and frequencies and percentages for categorical variables. We will plot histograms of continuous variables to assess normality.

Baseline characteristics will include the percentage of patients within each of the categories defined by the minimisation factors: trial site, age, gender, obesity, symptomatic or asymptomatic disease, current smoking status, ethnicity and presence or absence of comorbidity.

# Analysis

## Outcome definitions

### Primary outcome

The primary outcome is the upper respiratory tract viral load at Day 5.

*Method of measurement: quantitative polymerase chain reaction (PCR) performed on saliva samples.*

### Secondary outcomes

The secondary outcomes are the following:

- Proportion of participants with undetectable upper respiratory tract viral load after 5 days of therapy.

*Method of measurement: quantitative polymerase chain reaction (PCR) performed on saliva samples.*

- Proportion of participants with undetectable stool viral load after 7 days of therapy.

*Method of measurement: PCR performed on stool samples.*

- Rate of decrease in upper respiratory tract viral load during 7 days of therapy.

*Method of measurement: PCR performed on daily saliva samples.*

- Duration of fever following commencement of medication.

*Methods of measurement: daily body temperature records between Day 1 and Day 7 post-randomisation.*

- Proportion of participants with hepatotoxicity after 7 days of therapy.

*Method of measurement: standard diagnostic laboratory assays for liver transaminases, alkaline phosphatase and bilirubin.*

- Proportion of participants with other medication-related toxicity after 7 days of therapy and 14 days post-randomisation.

*Methods of measurement: determination of medication-related adverse events by investigators.*

- Proportion of participants admitted to hospital with COVID-19 related illness.

*Methods of measurement: participant self-report, review of hospital records and discharge summaries.*

- Proportion of participants admitted to ICU with COVID-19 related illness.

*Methods of measurement: participant self-report, review of hospital records and discharge summaries.*

- Proportion of participants who have died with COVID-19 related illness.

*Methods of measurement: next of kin report, review of hospital records and discharge summaries.*

- Pharmacokinetic and pharmacodynamic analysis of favipiravir.

*Method of measurement: assay of favipiravir levels in plasma at Day 7 of therapy. All participants from each arm will provide a pre-dose trough sample and a post-dose (30 to 60 min) sample on Day 7 of therapy. A nonlinear mixed effects model will be fitted jointly to favipiravir pharmacokinetic and viral load (pharmacodynamic) data.*

The model will estimate the following primary PK parameters:

- PK: Clearance (CL), Volume of distribution (V), Absorption rate constant (Ka)
- From which the following secondary parameters will be derived:
- Maximum concentration (Cmax),
- Time to maximum concentration (Tmax),
- Elimination rate constant (Ke),
- Area Under the Curve extrapolated to infinity (AUC (0-inf))
- The model will also estimate the following pharmacodynamic parameters:
- Rate of viral load decline (delta),
- Maximum increase in viral load under drug treatment (Emax),
- Concentration to achieve half the maximum possible effect (EC50)

### Exploratory outcome

Proportion of participants with deleterious or resistance-conferring mutations in SARS-CoV-2.

*Method of measurement: deep sequencing of virus and bioinformatic analysis.*

### Rationale and details for outcome measures

In viral infections such as influenza, it is well recognised that early antiviral therapy is associated with improved clinical outcome. Viral load falls rapidly and hence placebo group participants may have negative viral loads by day 7 of treatment^3^ (day 14 post symptom onset as subjects up to Day 7 post onset could be recruited in this trial). Therefore, Day 5 is the current best estimate of the time point at which drug-induced differences in viral loads are expected to be seen, and hence is the primary endpoint and time at which a formal assessment will be conducted.

Viral load is measured in copies/ml and is expected to be a positively skewed variable. The sample size calculation assumes that viral load data are log normally distributed, therefore data will be log transformed (log_10_) for analysis.

## Analysis methods

The results of the analyses will be reported following the principle of the ICH E3 guidelines on the Structure and Content of Clinical Study Reports^4^. Dummy tables are presented in the Appendix.

### Adjustment factors

The primary outcome model will be adjusted for baseline viral load. The secondary outcome model of rate of decrease of viral load will also be adjusted for baseline viral load.

### Primary outcome analysis

An analysis of covariance (ANCOVA) model will be used to estimate the difference in viral load at 5 days post treatment between the treatment groups.

The model will include the treatment effects of each factor, interaction between factors and baseline viral load. The model for viral load at day 5, where Y_ij_ is the viral load of patient i at time j (days), is:

| Y_i,5_ | = *β* _i_ + *β* _1_(Z_1i_) +* β* _2_(Z_2i_) + *β* _3_(Z_1i_ * Z_2i_) + *β* _4_(Y_i_,_1_) + *ε*_i_ |
| --- | --- |

Where, Z_1_ = dummy variable for factor 1 (0= favipiravir placebo, 1= favipiravir)

Z_2_ = dummy variable for factor 2 (0= LPV/r placebo, 1= LPV/r)

and *ε*_i_ ~ N(0,$\sigma^{2}$)

We will use the above model to estimate the treatment and interaction effects at Day 5:

- The effect of Favipiravir alone given by *β* _1_
- The effect of LPV/r alone given by *β* _2_
- The interaction effect given by *β* _3_

The model assumes linear relationship between outcome and factors and of normality, homogeneity and independence of errors which will be investigated using plots of residuals.

**Sensitivity analysis of primary outcome**

We plan to carry out the following sensitivity analyses on the primary outcome to assess the robustness of results:

1. The primary outcome model will be refitted adjusting for the minimisation factors. Factors will be included in the model if >10% patients are in a subgroup of a factor.
2. The primary model will be adjusted for potential effect of the Delta variant strain of the SARS-CoV-2 virus. Randomisations will be categorised into three periods – no-delta variant (before 24 April 2021), some-delta variant (between 24 April 2021 and 12 June 2021) and predominantly delta variant period (post 12 June 2021) and the model will have a fixed effect for period. The cut-off dates are based on national data.
3. Patients may have negative viral count because of the following reasons:

- The test result was a false positive at baseline.
- The level of virus was so low that viral load could not be detected in saliva.
- The participant used mouthwash before collecting the samples.

We plan to carry out a further sensitivity analysis that will exclude patients who have consistently negative viral count during the trial period.

1. We will consider using a joint model to carry out supportive analysis if we observe imbalance between groups in provision of trial data, in a high proportion of patients (>10%).
2. We may use causal mediation analysis to evaluate if potential differences in treatment adherence may mediate the effect of treatment on viral load.

### Secondary outcome analysis

**Continuous Secondary Outcomes**

A repeated measures linear regression model will be used to estimate the average differences in rate of decrease in upper respiratory tract viral load during 7 days of therapy between the treatment arms. This analysis method will use viral load at Day 2 to Day 7 as outcome to estimate the differences in rate of decrease in viral load.

The model will include fixed effects for treatment effects of factors, interaction between factors, time (Day 2 to Day 7) and interaction between time and factors. The model will also include baseline viral load (Day 1) as fixed effect. A random patient effect will be included to take account of clustering within patients.

**Categorical Secondary Outcomes**

The proportion of participants defined by each of the secondary outcomes below will be summarised by treatment arm. Separate logistic regression models will be fitted to compare effect of treatment on the binary outcomes:

- Proportion of participants with undetectable upper respiratory tract viral load after 5 days of therapy.
- Proportion of participants with undetectable stool viral load after 7 days of therapy post-randomisation.
- Proportion of participants with hepatotoxicity after 7 days of therapy post-randomisation.
- Proportion of participants with other medication-related toxicity after 7 days of therapy and 14 days post-randomisation.
- Proportion of participants admitted to hospital with COVID-19 related illness.
- Proportion of participants admitted to ICU with COVID-19 related illness.
- Proportion of participants who have died with COVID-19 related illness.

**Time-to-event outcome**

Duration of fever will be visually displayed using Kaplan-Meier curves, and the medians for the duration of fever will be calculated by the [Kaplan-Meier method](https://www.sciencedirect.com/topics/medicine-and-dentistry/kaplan-meier-method)^5^. The first date when daily body temperature record is observed to be within normal ranges will be the date of event for this analysis.

The comparison of duration of fever following commencement of medication between trial arms will be by t-test, non-parametric equivalent or Cox regression depending on the distribution of the data.

**Pharmacokinetic/Pharmacodynamic Outcome**

Population PKPD modelling and dosing simulations will be undertaken with non-linear mixed-effects modelling.

Viral load with time will be modelled with a viral dynamic model, and the influence of favipiravir included to estimate an in vivo EC50.

Viral dynamic model: A simplified target cell limited model will be fitted to viral load with time since symptom onset. The target cell limited model consists of three ordinary differential equations relating to changes in uninfected target cells (T), infected target cells (I) and free virus (V) over time (t), as follows:

(dT(t))/dt= -βT(t)V(t)

(dI(t))/dt= βT(t)V(t)-δI(t)

(dV(t))/dt= ρI(t)-cV(t)

where β is the rate at which target cells become infected in the presence of virus, δ is the death rate of infected cells, ρ is the rate of viral production from infected cells and c is the rate of clearance of free virus. This model is structurally unidentifiable unless the initial condition for T, β, or ρ are known. Furthermore, the elimination rate of free virus (c) is likely to be much faster than the death rate of infected cells (δ). Hence, by assuming a quasi-steady-state between I and V, and normalising the total cell number by the number of infected cells when observations begin (t = 0), it is then possible to reduce the model to a structurally identifiable, two state ordinary differential equation model relating to the fraction (f) of infected cells with time and infected cells as a proxy for viral load as follows^6^

(df(t))/dt= -βf(t)V(t)

(dV(t))/dt= γf(t)V(t)-δV(t)

with γ, a new parameter equal to ρβT0/c and interpreted to be the maximum rate of viral replication. δ can now be interpreted as overall viral elimination rate. This population model will be then fitted to viral load data with time using the following form:

y_ij=f(φ_i,t_ij )+ε_ij

where yij was the viral load from subject i at time tij, f is the nonlinear model defined above with parameters φi, and εij the residual between the model prediction and the observed data.

Drug effect will be tested on δ since this composite parameter can be interpreted as viral production rate (i.e. slower than elimination rate) and it has been shown that γ is not readily identifiable when fitting viral dynamic data.^7^  A simple fold increase in delta will be estimated for each drug alone and in combination.

In addition to the simplified target cell limited model the full model and model with eclipse phase will be tested .^7^ If this model gives superior fit (see model selection criteria below) it will be adopted instead of the reduced model.

Favipiravir pharmacokinetics: Plasma concentrations will be sent from the bioanalytical laboratory on a spreadsheet, and dosing history and relevant covariate information extracted from the clinical database. One and two compartment disposition models with first order absorption will be tested. For the favipiravir-containing arms, drug concentration will be incorporated into the viral dynamic model using an Emax model.

Data preparation and model building: These data will be imported into R (version 3.4 or above) and merged for exploratory analysis and formatted for subsequent modelling using NONMEM version 7.4 or above (Globomax, USA) and or nlmixr version 2.0 or above.

Model selection criteria will include: (i) successful minimisation, (ii) standard error of estimates, (iii) number of significant digits, (iv) termination of the covariance step and (v) correlation between model parameters. Goodness of fit will be assessed by graphical methods, including population and individual predicted vs. observed concentrations, conditional weighted residual vs. observed concentrations and time, correlation matrix for fixed vs. random effects, correlation matrix between parameters and covariates and normalised predictive distribution error (NPDE). Comparison of hierarchical models will be based on the likelihood ratio test. A superior model will be also expected to reduce inter-subject variance terms and/or residual error terms. Standard error of the parameter estimates will be approximated using of the asymptotic covariance matrix.

### Subgroup analysis

The regression model for the primary outcome will be extended by adding interaction terms to explore whether a differential treatment effect is observed in pre-specified subgroups, if >10% patients are in a subgroup of a factor. We will include interactions between treatments and the following factors: sex (male, female), age (≤ 55 vs > 55), obesity (BMI <30 vs ≥30), symptomatic or asymptomatic, current smoking status (Yes/No), ethnicity (Caucasian, other), vaccinated (Yes/No), comorbidity (Yes/No), baseline antibody status (seropositive vs seronegative) and whether patients started treatment within 5 days of symptom onset (Yes/No). The main treatment effect within subgroups will be estimated and interaction p-values will be reported. We will also use forest plots to graphically display treatment effects across the subgroups.

### Exploratory outcome analysis

Absolute number of mutations and descriptive statistics will be presented for each group. Samples with sufficient viral load for sequencing will be analysed on Day 1 and Day 5 (+/-1 days) for each patient.  The proportion of participants with deleterious or resistance-conferring mutations in SARS-CoV-2 may be compared between treatment arms using logistic regression.

Further post-hoc supportive analysis will be carried out based on the research findings.

# References

1. <https://www.cdc.gov.tw/File/Get/ht8jUiB_MI-aKnlwstwzvw> (Favipiravir (Avigan) tablets 200mg insert)
2. Schulz KF, Altman DG, and Moher D, *CONSORT 2010 Statement: updated guidelines for reporting parallel group randomised trials.* BMJ, 2010. **340**.
3. Wölfel 2020: Wölfel R. et al. Virological assessment of hospitalized patients with COVID-2019 Nature (2020). <https://doi.org/10.1038/s41586-020-2196-x>
4. International Conference on Harmonisation of Technical Requirements for Registration of Pharmaceuticals for Human Use. Structure and content of clinical study reports (E3). 1995.
5. Ikematsu H, Kawai N, Iwaki N, et al. *Duration of fever and other symptoms after the inhalation of laninamivir octanoate hydrate in the 2016/17 Japanese influenza season; comparison with the 2011/12 to 2015/16 seasons*. J Infect Chemother. 2018;24(9):718-724. doi:10.1016/j.jiac.2018.04.013
6. Kim KS, Ejima K, Iwanami S, Fujita Y, Ohashi H, Koizumi Y, Asai Y, Nakaoka S, Watashi K, Aihara K, Thompson RN, Ke R, Perelson AS, Iwami S. *A quantitative model used to compare within-host SARS-CoV-2, MERS-CoV, and SARS-CoV dynamics provides insights into the pathogenesis and treatment of SARS-CoV-2*. PLoS Biol. 2021 Mar 22;19(3):e3001128. doi: 10.1371/journal.pbio.3001128. PMID: 33750978; PMCID: PMC7984623.
7. Gastine S, Pang J, Boshier FAT, Carter SJ, Lonsdale DO, Cortina-Borja M, Hung IFN, Breuer J, Kloprogge F, Standing JF. *Systematic Review and Patient-Level Meta-Analysis of SARS-CoV-2 Viral Dynamics to Model Response to Antiviral Therapies*. Clin Pharmacol Ther. 2021 Aug;110(2):321-333. doi: 10.1002/cpt.2223. Epub 2021 May 1. PMID: 33641159; PMCID: PMC8014833.

# revision history

| **Version** | **Date** | **Edited by** | **Comments/Justification** | **Timing in relation to first unblinded interim monitoring** | **Timing in relation to unblinding of Trial Statistician(s)** |
| --- | --- | --- | --- | --- | --- |
| 0.1 | 30 Jul 2020 | KC | First draft | Prior | Prior |
| 0.2 | 27 Aug 2020 | KC | Amendments following input from Chris Frost and Hakim-Moulay Dehbi. | Prior | Prior |
| 0.3 | 25 Sep 2020 | KC | Amendments following further input from Hakim-Moulay Dehbi and Nick Freemantle. | Prior | Prior |
| 0.4 | 01 Mar 2021 | KC | Amendments following inputs from David Lowe | Post | Prior |
| 0.5 | 16 Jun 2021 | KC/ JS | Amendment following protocol changes. Clarification on wording of primary model following discussion with Chris Frost.  Expanded on PK/PD analysis. Added new sensitivity analyses plan. | Post | Post |
|  |  |  |  |  |  |
|  |  |  |  |  |  |
|  |  |  |  |  |  |
|  |  |  |  |  |  |
|  |  |  |  |  |  |
|  |  |  |  |  |  |

# APPENDICES

## Table 1: Baseline Characteristics

| **Minimisation factors** |  | **Favipiravir + LPV/r**  **(n = )** | **Favipiravir**  **(n = )** | **LPV/r**  **(n = )** | **Placebo**  **(n = )** | **Total**  **(N = )** |
| --- | --- | --- | --- | --- | --- | --- |
| Site | n (%) |  |  |  |  |  |
| Age (years) | n (%) |  |  |  |  |  |
| ≤ 55 |  |  |  |  |  |  |
| > 55 |  |  |  |  |  |  |
| Gender | n(%) |  |  |  |  |  |
| Female |  |  |  |  |  |  |
| Male |  |  |  |  |  |  |
| BMI (kg/m^2^) | n (%) |  |  |  |  |  |
| <30 |  |  |  |  |  |  |
| ≥30 |  |  |  |  |  |  |
| Symptomatic disease | n (%) |  |  |  |  |  |
| No |  |  |  |  |  |  |
| Yes |  |  |  |  |  |  |
| Current smoker | n (%) |  |  |  |  |  |
| No |  |  |  |  |  |  |
| Yes |  |  |  |  |  |  |
| Ethnicity (reclassified) | n (%) |  |  |  |  |  |
| Caucasian |  |  |  |  |  |  |
| Other |  |  |  |  |  |  |
| Comorbidity | n (%) |  |  |  |  |  |
| No |  |  |  |  |  |  |
| Yes |  |  |  |  |  |  |
| Vaccinated |  |  |  |  |  |  |
| No |  |  |  |  |  |  |
| Yes |  |  |  |  |  |  |
| **Characteristics at screening** |  |  |  |  |  |  |
| Age (years) | mean(sd) |  |  |  |  |  |
| Height (cm) | mean(sd) |  |  |  |  |  |
| Weight (kg) | mean(sd) |  |  |  |  |  |
| Ethnicity | n(%) |  |  |  |  |  |
| White |  |  |  |  |  |  |
| Mixed |  |  |  |  |  |  |
| Black or Black British |  |  |  |  |  |  |
| Asian or Asian British |  |  |  |  |  |  |
| Other Ethnic groups |  |  |  |  |  |  |
| Smoking status | n(%) |  |  |  |  |  |
| No |  |  |  |  |  |  |
| Yes |  |  |  |  |  |  |
| Ex-smoker | n(%) |  |  |  |  |  |
| No |  |  |  |  |  |  |
| Yes |  |  |  |  |  |  |
| Time since cessation | mean(sd) |  |  |  |  |  |
| Comorbidity history | n(%) |  |  |  |  |  |
| Diabetes Type I |  |  |  |  |  |  |
| Diabetes Type II |  |  |  |  |  |  |
| Ischaemic Heart Disease (Myocardial Infarction) |  |  |  |  |  |  |
| Other Heart Disease (Arrhythmia/Valvular Heart Disease) |  |  |  |  |  |  |
| Asthma |  |  |  |  |  |  |
| Chronic Obstructive Pulmonary Disease (COPD) |  |  |  |  |  |  |
| Other Chronic Respiratory Disease |  |  |  |  |  |  |
| HIV status | n(%) |  |  |  |  |  |
| Positive |  |  |  |  |  |  |
| Negative |  |  |  |  |  |  |
| Unknown |  |  |  |  |  |  |
| **Clinical factors at screening** |  |  |  |  |  |  |
| Pulse Rate (bpm) | mean(sd) |  |  |  |  |  |
| Respiratory Rate (bpm) | mean(sd) |  |  |  |  |  |
| Body Temperature (°C) | mean(sd) |  |  |  |  |  |
| Viral load (log_10_) | mean(sd) |  |  |  |  |  |

**Table 2: Primary outcome**

| **Outcome** | **Placebo** | | **Favipiravir** | | | | **LPV/r** | | | | **Favipiravir+ LPV/r** | | | |
| --- | --- | --- | --- | --- | --- | --- | --- | --- | --- | --- | --- | --- | --- | --- |
|  | mean(sd) | | mean(sd) | | Coefficient  (95% CI) | p-value | mean(sd) | | Coefficient  (95% CI) | p-value | mean(sd) | | Coefficient  (95% CI) | p-value |
|  | Baseline | Day 5 | Baseline | Day 5 |  |  | Baseline | Day 5 |  |  | Baseline | Day 5 |  |  |
| **Primary outcome** | | | | | | | | | | | | | | |
| Viral load (log_10_) - ITT |  |  |  |  |  |  |  |  |  |  |  |  |  |  |
| **Sensitivity Analysis of Primary outcome** | | | | | | | | | | | | | | |
| Viral load (log_10_) – modified ITT |  |  |  |  |  |  |  |  |  |  |  |  |  |  |

ITT = Intention-to-treat

**Table 3: Secondary outcomes**

| **Outcome** | **Placebo** | **Favipiravir** | | | **LPV/r** | | | **Favipiravir+ LPV/r** | | |
| --- | --- | --- | --- | --- | --- | --- | --- | --- | --- | --- |
|  | mean(sd) | mean(sd) | Coefficient (95% CI) | p-value | mean(sd) | Coefficient (95% CI) | p-value | mean(sd) | Coefficient (95% CI) | p-value |
| Rate of decrease viral load (log_10_) during 7 days |  |  |  |  |  |  |  |  |  |  |
|  | Med  (IQR) | Med  (IQR) | Adjusted hazard ratio (95% CI) | p-value | Med (IQR) | Adjusted hazard ratio (95% CI) | p-value | Med (IQR) | Adjusted hazard ratio (95% CI) | p-value |
| Duration of fever |  |  |  |  |  |  |  |  |  |  |
|  | n (%) | n (%) | Adjusted odds ratio (95% CI) | p-value | n (%) | Adjusted odds ratio (95% CI) | p-value | n (%) | Adjusted odds ratio (95% CI) | p-value |
| Undetectable viral load (saliva) at Day5 |  |  |  |  |  |  |  |  |  |  |
| Undetectable viral load (stool) at Day7 |  |  |  |  |  |  |  |  |  |  |
| Undetectable viral load (stool) at Day14 |  |  |  |  |  |  |  |  |  |  |
| Hepatotoxicity at Day7 |  |  |  |  |  |  |  |  |  |  |
| Medication-related toxicity at Day7 |  |  |  |  |  |  |  |  |  |  |
| Medication-related toxicity at Day14 |  |  |  |  |  |  |  |  |  |  |
| Admitted to hospital with COVID-19 related illness |  |  |  |  |  |  |  |  |  |  |
| Admitted to ICU with COVID-19 related illness |  |  |  |  |  |  |  |  |  |  |
| Died with COVID-19 related illness |  |  |  |  |  |  |  |  |  |  |

**Table 4: Adverse events and Serious Adverse events**

|  | **Favipiravir + LPV/r** | **Favipiravir** | **LPV/r** | **Placebo** | **Total** |
| --- | --- | --- | --- | --- | --- |
| Number of Patients reporting at least 1 SAE/ SAR/ SUSAR, n(%) |  |  |  |  |  |
| *Number of Events*  SAE |  |  |  |  |  |
| SAR |  |  |  |  |  |
| SUSAR |  |  |  |  |  |
| Total |  |  |  |  |  |
| Number of Patients reporting at least 1 AE, n(%) |  |  |  |  |  |
| Total number of AEs, n |  |  |  |  |  |

**Table 5: Subgroup analyses**

| **Outcomes** | | **Placebo** | **Favipiravir** | | | **LPV/r** | | | **Favipiravir+ LPV/r** | | |
| --- | --- | --- | --- | --- | --- | --- | --- | --- | --- | --- | --- |
|  |  | Mean  (sd) | Mean  (sd) | Coefficient (95% CI) | Interaction  p-value | Mean  (sd) | Coefficient (95% CI) | Interaction  p-value | Mean  (sd) | Coefficient (95% CI) | Interaction  p-value |
| Age(years) | ≤55 |  |  |  |  |  |  |  |  |  |  |
|  | >55 |  |  |  |  |  |  |  |  |  |  |
| Gender | Female |  |  |  |  |  |  |  |  |  |  |
|  | Male |  |  |  |  |  |  |  |  |  |  |
| BMI(kg/m^2^) | <30 |  |  |  |  |  |  |  |  |  |  |
|  | ≥30 |  |  |  |  |  |  |  |  |  |  |
| Symptomatic disease | No |  |  |  |  |  |  |  |  |  |  |
|  | Yes |  |  |  |  |  |  |  |  |  |  |
| Current  smoker | No |  |  |  |  |  |  |  |  |  |  |
|  | Yes |  |  |  |  |  |  |  |  |  |  |
| Ethnicity | Caucasian |  |  |  |  |  |  |  |  |  |  |
|  | Other^1^ |  |  |  |  |  |  |  |  |  |  |
| Comorbidity | No |  |  |  |  |  |  |  |  |  |  |
|  | Yes |  |  |  |  |  |  |  |  |  |  |
| Vaccinated | No |  |  |  |  |  |  |  |  |  |  |
|  | Yes |  |  |  |  |  |  |  |  |  |  |

1. Includes patients who identify themselves as Black/ Black British, Asian/ Asian British, Mixed or Other ethnic group.
