# Supplementary material for: Favipiravir, lopinavir-ritonavir, or combination therapy (FLARE): A randomised, double-blind, 2 × 2 factorial placebo-controlled trial of early antiviral therapy in COVID-19
Source: PLoS Med. 2022 Oct 19;19(10):e1004120. doi: 10.1371/journal.pmed.1004120 (PMC9629589; doi:10.1371/journal.pmed.1004120)
Supplement: S1 Trial Protocol — (DOCX) [file pmed.1004120.s017.docx]

**FLARE**

**Favipiravir +/- Lopinavir: A RCT of Early antivirals**

Favipiravir, lopinavir/ritonavir or combination therapy: a randomised, double blind, 2x2 factorial placebo-controlled trial of early antiviral therapy in COVID-19

| Version | V6.0 |
| --- | --- |
| Date | 8^th^ June 2021 |
| Sponsor | University College London (UCL) |
| Comprehensive Clinical Trials Unit Trial Adoption Group # | CTU/2020/354 |
| Trial registration | NCT04499677 |
| EudraCT # | 2020-002106-68 |
| REC # | 20/WA/0210 |

Authorisation: Chief Investigator

| Name | Dr David Lowe |
| --- | --- |
| Role | Chief Investigator |
| Signature |  |
| Date |  |

Authorisation: Sponsor/CCTU Director Representative

| Name | Professor Nick Freemantle |  |
| --- | --- | --- |
| Role | Director, CCTU |  |
| Signature |  |  |
| Date |  |  |

Authorisation: Senior Operations Staff

| Name | Ms Gemma Jones |  |
| --- | --- | --- |
| Role | Head of Clinical Trial Operations, CCTU |  |
| Signature |  |  |
| Date |  |  |

Authorisation: Senior Statistician

| Name | Dr Hakim-Moulay Dehbi |  |
| --- | --- | --- |
| Role | Head of Statistics |  |
| Signature |  |  |
| Date |  |  |

Table of Contents

[1 Administrative information 9](#_Toc74149606)

[1.1 Compliance 9](#_Toc74149607)

[1.2 Sponsor 10](#_Toc74149608)

[1.3 Structured trial summary 11](#_Toc74149609)

[1.4 Roles and responsibilities 15](#_Toc74149610)

[1.4.1 Protocol contributors 15](#_Toc74149611)

[1.4.2 Role of trial sponsor and funders 15](#_Toc74149612)

[1.4.3 Trial Team 16](#_Toc74149613)

[1.4.4 Trial Management Group 16](#_Toc74149614)

[1.4.5 Trial Steering Committee 17](#_Toc74149615)

[1.4.6 Independent Data Monitoring Committee 17](#_Toc74149616)

2 Trial Diagram 18

[3 Abbreviations 20](#_Toc74149619)

[4 Glossary 21](#_Toc74149620)

[5 Introduction 22](#_Toc74149621)

[5.1 Background and Rationale 22](#_Toc74149622)

[5.1.1 Summary 22](#_Toc74149623)

[5.1.2 Detailed scientific background and rationale 23](#_Toc74149624)

[5.2 Objectives 27](#_Toc74149625)

[5.3 Trial Design 27](#_Toc74149626)

[6 Methods 29](#_Toc74149627)

[6.1 Site Selection 29](#_Toc74149628)

[6.1.1 Study Setting 29](#_Toc74149629)

[*6.1.2 Site/Investigator Eligibility Criteria 29*](#_Toc74149630)

[6.1.2.1 Principal Investigator’s (PI) Qualifications and Agreements 29](#_Toc74149631)

[6.1.2.2 Resourcing at site 30](#_Toc74149632)

[6.2 Site approval and activation 30](#_Toc74149633)

[6.3 Participants 30](#_Toc74149634)

[6.3.1 Eligibility Criteria 30](#_Toc74149635)

[6.3.1.1 Participant selection 30](#_Toc74149636)

[6.3.1.2 Participant Inclusion Criteria 31](#_Toc74149637)

[6.3.1.3 Participant Exclusion Criteria 32](#_Toc74149638)

[6.3.1.4 Women of Child Bearing Potential (WOCBP) 32](#_Toc74149639)

[6.3.1.5 Male Participants 33](#_Toc74149640)

[6.3.1.6 Eligibility Criteria for Individuals Performing the Interventions 34](#_Toc74149641)

[6.3.1.7 Co-enrolment Guidance 34](#_Toc74149642)

[6.3.1.8 Screening Procedures and Pre-randomisation Investigations 34](#_Toc74149643)

[6.4 Interventions 36](#_Toc74149644)

[6.4.1. Products and Treatment Schedule 36](#_Toc74149645)

[6.4.1.1 Packaging, labelling and QP release 36](#_Toc74149646)

[6.4.1.2 Favipiravir and placebo 37](#_Toc74149647)

[6.4.1.3 Lopinavir/ritonavir and placebo 37](#_Toc74149648)

[6.4.1.4 Dose modifications, interruptions and discontinuations 39](#_Toc74149649)

[6.4.2 Dispensing 40](#_Toc74149650)

[6.4.3 Accountability 40](#_Toc74149651)

[6.4.4 Concomitant Care 41](#_Toc74149652)

[6.4.5 Overdose of Trial Medication 41](#_Toc74149653)

[6.4.6 Protocol Treatment Discontinuation 41](#_Toc74149654)

[6.5 Sample Management 42](#_Toc74149655)

[6.5.1 Schedule of samples 42](#_Toc74149656)

[6.5.1.1 Pharmacokinetic sampling 43](#_Toc74149657)

[6.5.2 Processing of samples 43](#_Toc74149658)

[6.6 Outcomes 44](#_Toc74149659)

[6.6.1 Primary Outcomes 44](#_Toc74149660)

[6.6.2 Secondary Outcomes 44](#_Toc74149661)

[6.7 Participant Timeline 43](#_Toc74149662)

[6.7.1 Early Stopping of Follow-up 45](#_Toc74149663)

[6.7.2 Participant Transfers 45](#_Toc74149664)

[6.7.3 Loss to Follow-up 45](#_Toc74149665)

[6.7.4 Trial Closure 45](#_Toc74149666)

[6.8 Sample Size 45](#_Toc74149667)

[6.9 Recruitment and Retention 46](#_Toc74149668)

[6.9.1 Recruitment 46](#_Toc74149669)

[6.9.2 Retention 46](#_Toc74149670)

[6.10 Assignment of Intervention 46](#_Toc74149671)

[6.10.1 Allocation 46](#_Toc74149672)

[6.10.1.1 Sequence generation 46](#_Toc74149673)

[6.10.1.2 Randomisation concealment mechanism 47](#_Toc74149674)

[6.10.1.3 Randomisation Implementation 47](#_Toc74149675)

[6.10.2 Blinding 47](#_Toc74149676)

[6.10.3 Emergency Unblinding 47](#_Toc74149677)

[6.10.4 Unblinding for the submission of SUSAR reports 48](#_Toc74149678)

[6.11 Data Collection, Management and Analysis 48](#_Toc74149679)

[6.11.1 Data Collection Methods 48](#_Toc74149680)

[6.11.2 Data Management 48](#_Toc74149681)

[6.11.3 Non-Adherence and Non-Retention 49](#_Toc74149682)

[6.11.4 Statistical Methods 50](#_Toc74149683)

[6.11.4.1 Statistical Analysis Plan 50](#_Toc74149684)

[6.11.4.2 Statistical Methods – Outcomes 50](#_Toc74149685)

[6.11.4.3 Additional Analyses – Subgroup 50](#_Toc74149686)

[6.11.4.4 Additional Analyses – Adjusted 50](#_Toc74149687)

[6.11.5 Analysis Population and Missing Data 50](#_Toc74149688)

[6.11.6 Pharmacometric evaluations 51](#_Toc74149689)

[6.11.6.1 Pharmacometric Analysis Plan 51](#_Toc74149690)

[6.11.6.2 Additional analyses 51](#_Toc74149691)

[6.12 Data Monitoring 51](#_Toc74149692)

[6.12.1 Data Monitoring Committee 51](#_Toc74149693)

[6.12.2 Interim Analyses 52](#_Toc74149694)

[6.12.3 Data Monitoring for Harm 52](#_Toc74149695)

[6.12.3.1 Safety reporting 52](#_Toc74149696)

[*6.12.3.1.1 Adverse Reactions* 53](#_Toc74149697)

[*6.12.3.1.2 Adverse Events* 53](#_Toc74149698)

[6.12.3.4 Procedures to follow in the event of female participants or female partners of male participants becoming pregnant 54](#_Toc74149699)

[6.12.3.5 Investigator responsibilities relating to safety reporting 54](#_Toc74149700)

[6.12.3.5.1 Seriousness assessment 54](#_Toc74149701)

[6.12.3.5.2 Severity or grading of Adverse Events 54](#_Toc74149702)

[6.12.3.5.3 Causality 55](#_Toc74149703)

[6.12.3.5.4 Expectedness 55](#_Toc74149704)

[6.12.3.6 Notifications 56](#_Toc74149705)

[6.12.3.6.1 Notifications by the Investigator to CCTU 56](#_Toc74149706)

[6.12.3.6.2 CCTU responsibilities 56](#_Toc74149707)

[6.12.4 Quality Assurance and Control 57](#_Toc74149708)

[6.12.4.1 Definitions 57](#_Toc74149709)

[6.12.4.2 Quality and Risk Management 57](#_Toc74149710)

[6.12.4.3 Central Monitoring at CCTU 57](#_Toc74149711)

[6.12.4.3 On-site Monitoring 58](#_Toc74149712)

[6.12.4.3.1 Direct access to participant records 58](#_Toc74149713)

[6.12.4.4 Trial Oversight 58](#_Toc74149714)

[6.12.4.4.1 Trial Team 58](#_Toc74149715)

[6.12.4.4.2 Trial Management Group 58](#_Toc74149716)

[6.12.4.4.3 Independent Trial Steering Committee 58](#_Toc74149717)

[6.12.4.4.4 Independent Data Monitoring Committee 58](#_Toc74149718)

[6.12.4.4.5 Trial Sponsor 59](#_Toc74149719)

[7 Ethics and Dissemination 59](#_Toc74149720)

[7.1 Ethics Committee Approval 59](#_Toc74149721)

[7.2 Competent Authority Approvals 59](#_Toc74149722)

[7.3 Other Approvals 60](#_Toc74149723)

[7.4 Protocol Amendments 60](#_Toc74149724)

[7.5 Consent or Assent 60](#_Toc74149725)

[7.6 Confidentiality 61](#_Toc74149726)

[7.7 Declaration of Interests 61](#_Toc74149727)

[7.8 Indemnity 61](#_Toc74149728)

[7.9 Finance 62](#_Toc74149729)

[7.10 Archiving 62](#_Toc74149730)

[7.11 Access to Data 62](#_Toc74149731)

[7.12 Ancillary and Post-trial Care 62](#_Toc74149732)

[7.13 Publication Policy 62](#_Toc74149733)

[7.13.1 Trial Results 62](#_Toc74149734)

[7.13.2 Authorship 62](#_Toc74149735)

[7.13.3 Reproducible Research 63](#_Toc74149736)

[8 Ancillary Studies 64](#_Toc74149737)

[9 Protocol Amendments 65](#_Toc74149738)

[10 References 70](#_Toc74149739)

[11 Appendices 72](#_Toc74149740)

[11.1 Appendix 1 Figures referred to in the text 72](#_Toc74149741)

[11.2 Appendix 2 Drug ingredients and excipients 75](#_Toc74149742)

[11.3 Appendix 3 Drug interactions and contraindications 76](#_Toc74149743)

[11.4 Appendix 4 Tabulated list of adverse reactions associated with the trial IMPs 80](#_Toc74149744)

[11.5 Appendix 5 PPE Guidance (Applicable for the UK sites only) 83](#_Toc74149745)

[11.6 Appendix 6 COUNTRY-SPECIFIC ADDENDUM 84](#_Toc74149746)

# 1 Administrative information

This document was constructed using the Comprehensive Clinical Trials Unit (CCTU) at UCL Protocol template Version 5. It describes the FLARE trial, sponsored by UCL and co-ordinated by CCTU.

It provides information about procedures for entering participants into the trial, and provides sufficient detail to enable: an understanding of the background, rationale, objectives, trial population, intervention, methods, statistical analyses, ethical considerations, dissemination plans and administration of the trial; replication of key aspects of trial methods and conduct; and appraisal of the trial’s scientific and ethical rigour from the time of ethics approval through to dissemination of the results. The protocol should not be used as an aide-memoire or guide for the treatment of other patients. Every care has been taken in drafting this protocol, but corrections or amendments may be necessary. These will be circulated to registered investigators in the trial. Sites entering participants for the first time should confirm they have the correct version through a member of the trial team at CCTU.

CCTU supports the commitment that its trials adhere to the SPIRIT guidelines. As such, the protocol template is based on an adaptation of the Medical Research Council CTU protocol template (2013) and the Standard Protocol Items: Recommendations for Interventional Trials (SPIRIT) 2013 Statement for protocols of clinical trials ^1^. The SPIRIT Statement Explanation and Elaboration document ^2^ can be referred to, or a member of CCTU Protocol Review Committee can be contacted for further detail about specific items.

## 1.1 Compliance

The trial will be conducted in compliance with the approved protocol, the Declaration of Helsinki (2008), the principles of Good Clinical Practice (GCP) as laid down by the Commission Directive 2005/28/EC with implementation in national legislation in the UK by Statutory Instrument 2004/1031 and subsequent amendments, the Human Tissue (Quality and Safety for Human Application) Regulations 2007, the UK Data Protection Act (2018), and the National Health Service (NHS) UK Policy Framework for Health and Social Care (2017). International sites will comply with the principles of GCP as laid down by ICH topic E6 (Note for Guidance on GCP), Commission Directive 2005/28/EC, the European Directive 2001/20/EC (where applicable), the EU Tissue and Cells Directives 2004/23/EC, 2006/17/EC and 2006/86/EC, and other national and local applicable regulations. Agreements that include detailed roles and responsibilities will be in place between participating sites and CCTU.

Participating sites will inform CCTU as soon as they are aware of a possible serious breach of this protocol or GCP, so that CCTU can fulfil its requirement to report the breach if necessary, within the timelines specified in the UK Clinical Trials Regulations (currently 7 days). For the purposes of this regulation a ‘serious breach’ is one that is likely to affect to a significant degree:

- The safety or physical or mental integrity of the subjects in the trial, or
- The scientific value of the trial.

## 1.2 Sponsor

UCL is the trial sponsor and has delegated responsibility for the overall management of the FLARE trial to CCTU. Queries relating to UCL sponsorship of this trial should be addressed to the CCTU Director or via the Trial Team.

## 1.3 Structured trial summary

| Primary Registry and Trial Identifying Number | Clinicaltrials.gov: NCT04499677 |
| --- | --- |
| Date of Registration in Primary Registry | 04^th^ August 2020 |
| Secondary Identifying Numbers | EudraCT #: 2020-002106-68  CCTU Trial Adoption Group #: CTU/2020/354  IRAS #: 284664  UCL R&D ID: 132084 |
| Source of Monetary or Material Support | LifeArc Covid-19 funding award grant reference [COVID0005] |
| Sponsor | University College London with sponsor responsibilities delegated to CCTU. |
| Contact for Public Queries | UCL Comprehensive Clinical Trials Unit,  90 High Holborn, 2nd Floor, London, WC1V 6LJ  cctu.flare@ucl.ac.uk  cctu.enquiries@ucl.ac.uk |
| Contact for Scientific Queries | Dr David Lowe  d.lowe@ucl.ac.uk  Institute of Immunity and Transplantation  University College London, Royal Free Campus  Pond Street  London NW3 2QG  United Kingdom |
| Public Title | **FLARE: Favipiravir +/- Lopinavir: A RCT of Early antivirals**  A Trial to compare Favipiravir, lopinavir/ritonavir, the combination of both drugs or dummy treatment only for early COVID-19 (Coronavirus) disease. |
| Scientific Title | Favipiravir, lopinavir/ritonavir or combination therapy: a randomised, double blind, 2x2 factorial placebo-controlled trial of early antiviral therapy in COVID-19 |
| Countries of Recruitment | United Kingdom and up to four sites internationally including sites in Malaysia and Kenya in the first instance. |
| Health Condition(s) or Problem(s) Studied | Any adults with highly suspected infection within the first 5 days of symptom onset OR who have tested positive with SARS-CoV-2 causing COVID-19 within the first 7 days of symptom onset OR who are asymptomatic but tested positive for SARS-CoV-2 for the first time within the last 48 hours. |
| Intervention(s) | **Arm 1**: **Favipiravir + Lopinavir/ritonavir (LPV/r)**  Oral favipiravir 1800 mg twice daily on Day 1, followed by 400 mg four (4) times daily from Day 2 to Day 7 **PLUS** Lopinavir/ritonavir (LPV/r) at 400mg/100 mg twice daily on Day 1 followed by 200mg/50mg four (4) times daily from Day 2 to Day 7  **Arm 2**: **Favipiravir + Lopinavir/ritonavir (LPV/r) placebo**  Oral favipiravir, 1800 mg twice daily on Day 1, followed by 400 mg four (4) times daily from Day 2 to Day 7 **PLUS** Lopinavir/ritonavir (LPV/r) matched placebo at 400mg/100 mg twice daily on Day 1, followed by 200mg/50mg four (4) times daily from Day 2 to Day 7.  **Arm 3**: **Favipiravir placebo + Lopinavir/ritonavir (LPV/r)**  Oral favipiravir matched placebo 1800 mg twice daily on Day 1, followed by 400 mg four (4) times daily from Day 2 to Day 7 **PLUS** Lopinavir/ritonavir (LPV/r) at 400mg/100 mg twice daily on Day 1, followed by 200mg/50mg four (4) times daily from Day 2 to Day 7.  **Arm 4**: **Favipiravir placebo + Lopinavir/ritonavir (LPV/r) placebo**  Oral favipiravir matched placebo 1800 mg twice daily on Day 1, followed by 400 mg four (4) times daily from Day 2 to Day 7 **PLUS** Lopinavir/ritonavir (LPV/r) matched placebo at 400mg/100 mg twice daily on Day 1 followed by 200mg/50mg four (4) times daily from Day 2 to Day 7. |
| Key Inclusion and Exclusion Criteria | Inclusion criteria are as follows:   1. Any adult with the following:  - Symptoms compatible with COVID-19 disease (Fever >37.8^o^C on at least one occasion AND either cough and/ or anosmia) within the first 5 days of symptom onset (date/time of enrolment must be within the first 5 days of symptom onset) - **OR** ANY symptoms compatible with COVID-19 disease (may include, but are not limited to fever, cough, shortness of breath, malaise, myalgia, headache, coryza) and tested positive for SARS-CoV-2 within the first 7 days of symptom onset) (date/time of enrolment must be within the first 7 days of symptom onset) - **OR** no symptoms but tested positive for SARS-CoV-2 within the last 48 hours (date/time of test must be within 48 hours of enrolment)  1. Male or female aged 18 years to 70 years old inclusive at screening 2. Willing and able to take daily saliva samples 3. Able to provide full informed consent and willing to comply with trial-related procedures   Exclusion criteria are as follows:   1. Known hypersensitivity to any of the active ingredients or excipients in favipiravir and matched placebo, and in lopinavir/ritonavir and matched placebo (**See Appendix 2**) 2. Chronic liver disease at screening (known cirrhosis of any aetiology, chronic hepatitis (e.g. autoimmune, viral, steatohepatitis), cholangitis or any known elevation of liver aminotransferases with AST or ALT > 3 X ULN)***** 3. Chronic kidney disease (stage 3 or beyond) at screening: eGFR < 60 ml/min/1.73m^2^ ***** 4. HIV infection, if untreated, detectable viral load or on protease inhibitor therapy 5. Any clinical condition which the investigator considers would make the participant unsuitable for the trial 6. Concomitant medications known to interact with favipiravir and matched placebo, and with lopinavir/ritonavir and matched placebo, and carry risk of toxicity for the participant (**See Appendix 3**) 7. Current severe illness requiring hospitalisation^**^ 8. Pregnancy and/ or breastfeeding 9. Eligible female participants of childbearing potential and male participants with a partner of childbearing potential not willing to use highly effective contraceptive measures during the trial and within the time point specified following last trial treatment dose. 10. Participants enrolled in any other interventional drug or vaccine trial (co-enrolment in observational studies is acceptable)***   *****Considering the importance of early treatment of COVID-19 to impact viral load, the absence of chronic liver/ kidney disease will be confirmed verbally by the participant during pre-screening and Screening/Baseline visit. Safety blood samples will be collected at Screening/Baseline visit (Day 1) and test results will be examined as soon as they become available within 24 hours.  **Hospitalisation for isolation or infection control reasons will not be considered an exclusion for this trial for international sites.  ***Participants that have received the COVID-19 vaccine can be included in this trial. |
| Study Type | **Trial design**: Multi-site, UK and international, Phase IIA randomised, double-blind, 2x2 factorial placebo-controlled, interventional trial.  **Randomisation**: Participants will be randomised 1:1:1:1 using a concealed online minimisation process, with the following factors: trial site, age (≤ 55 vs > 55 years old), gender, obesity (BMI <30 vs ≥30), symptomatic or asymptomatic, current smoking status (Yes = current smoker, No = ex-smoker, never smoker), ethnicity (Caucasian, other), previous COVID-19 specific vaccination (Yes = ≥ 1 dose, No = no previous covid-19 specific vaccination) and presence or absence of comorbidity (defined as diabetes, hypertension, ischaemic heart disease (including previous myocardial infarction), other heart disease (arrhythmia and valvular heart disease), asthma, COPD, other chronic respiratory disease).    **Blinding**: Participants and investigators will both be blinded to treatment allocation (double-blind). |
| Date of First Enrolment | Actual date: October 2020 |
| Target Sample Size | 240 participants, 60 in each arm |
| Primary Outcome(s) | Upper respiratory tract viral load at Day 5. |
| Key Secondary Outcomes | - Percentage of participants with undetectable upper respiratory tract viral load after 5 days of therapy - Proportion of participants with undetectable stool viral load after 7 days of therapy^*^ - Rate of decrease in upper respiratory tract viral load during 7 days of therapy - Duration of fever following commencement of trial medications - Proportion of participants with hepatotoxicity after 7 days of therapy - Proportion of participants with other medication-related toxicity after 7 days of therapy and 14 days post-randomisation - Proportion of participants admitted to hospital with COVID-19 related illness - Proportion of participants admitted to ICU with COVID-19 related illness - Proportion of participants who have died with COVID-19 related illness - Pharmacokinetic and pharmacodynamic analysis of favipiravir - Exploratory: Proportion of participants with deleterious or resistance-conferring mutations in SARS-CoV-2   *Secondary outcome not applicable for international sites as stool samples will not be collected internationally. |

## 1.4 Roles and responsibilities

These membership lists are correct at the time of writing; please see terms of reference documentation in the TMF for current lists.

### 1.4.1 Protocol contributors

| Name | Affiliation | Role |
| --- | --- | --- |
| David Lowe | UCL Institute of Immunity and Transplantation | Chief Investigator |
| Joe Standing | ICH-UCL | Co-Investigator |
| Judy Breuer | UCL | Co-Investigator |
| Li-An Brown | UCL Institute of Immunity and Transplantation | Co-Investigator |
| Nick Freemantle | UCL CCTU | Co-Investigator |
| Hakim-Moulay Dehbi | UCL CCTU | Co-Investigator |
| Gemma Jones | UCL CCTU | Head of Clinical Trial Operations |
| Felicia Ikeji | UCL CCTU | Clinical Project Manager |
| Amalia Ndoutoumou | UCL CCTU | Trial Manager |

### 1.4.2 Role of trial sponsor and funders

| Name | Affiliation | Role |
| --- | --- | --- |
| UCL | UCL | Trial Sponsor |
| UCL CCTU | UCL | UCL is the trial sponsor and has delegated the sponsor’s duties to UCL CCTU. The role of the sponsor is to take on responsibility for securing the arrangements to initiate, manage and finance the trial. A Clinical Project Manager at the UCL CCTU will oversee the Trial Manager who will be responsible for the day-to-day management of the trial and providing support to the site staff. The CCTU staff will be involved in approaching sites, case report form development, database construction, protocol and patient information in collaboration with the Trial Management Team. |
| LifeArc | N/A | Trial Funders |

###

### 1.4.3 Trial Team

| Name | Affiliation | Role and responsibilities |
| --- | --- | --- |
| David Lowe | UCL Institute of Immunity and Transplantation, Royal Free Campus | Chief Investigator |
| Joe Standing | Great Ormond Street Institute of Child Health, UCL | Co-Investigator |
| Judy Breuer | Great Ormond Street Institute of Child Health, UCL | Co-Investigator |
| Li-An Brown | UCL Institute of Immunity and Transplantation, Royal Free Campus | Co-Investigator |
| Nick Freemantle | UCL CCTU | Co-Investigator |
| Felicia Ikeji | UCL CCTU | Clinical Project Manager |
| Trial Manager | UCL CCTU | Trial Manager |
| Hakim-Moulay Dehbi | UCL CCTU | Co-Investigator and oversight statistician |
| Kashfia Chowdhury | UCL CCTU | Trial Statistician |
| Krishneya Santhirakumar | UCL CCTU | Data Manager |

### 1.4.4 Trial Management Group

| Name | Affiliation | Role and responsibilities |
| --- | --- | --- |
| David Lowe | UCL | Chief Investigator and Chair |
| Joe Standing | ICH-UCL | Member |
| Judy Breuer | UCL | Member |
| Nick Freemantle | UCL CCTU | Member |
| Gemma Jones | UCL CCTU | Member |
| Nicky Longley | UCLH | Member |
| Anna Checkley | UCLH | Member |
| Felicia Ikeji | UCL CCTU | Member |
| Trial Manager | UCL CCTU | Member and facilitator |
| Hakim-Moulay Dehbi | UCL CCTU | Member |
| Kashfia Chowdhury | UCL CCTU | Member |
| Krishneya Santhirakumar | UCL CCTU | Member and facilitator |
| Li-An Brown | UCL | Member |

### 1.4.5 Trial Steering Committee

| Name | Affiliation | Role and responsibilities |
| --- | --- | --- |
| Dr Kristina Nadrah | ID Specialist and Assistant Professor in Infectious Diseases and Epidemiology - University Medical Centre Ljubljana | TSC Chair |
| Prof Rob Read | Head of Clinical and Experimental Sciences & Professor of Infectious Diseases - University of Southampton | TSC Member |
| Prof Liz Allen | Professor of Medical Statistics - London School of Hygiene & Tropical Medicine | TSC Member |
| Prof Chris Frost | Professor of Medical Statistics – London School of Hygiene & Tropical Medicine | TSC Member (non-independent) |
| Ms Mahdia Sait | Patient representative | TSC Member |

###

### 1.4.6 Independent Data Monitoring Committee

| Name | Affiliation | Role and responsibilities |
| --- | --- | --- |
| Dr Stephen Owens | Newcastle Upon Tyne Hospitals NHS Foundation Trust | Chair |
| Ms April Slee | New Arch Consulting, Seattle USA | Independent Statistician (Member) |
| Professor Andrew Ustianowski | Principal Clinical Research Lead, North Manchester General Hospital | Independent Clinician (Member) |
| Dr David Chadwick | South Tees Hospital NHS Foundation Trust | Independent Clinician (Member) |

#

# 2 Trial Diagram

**Recruitment pathway**

#

- **Targeted advertising (posters, flyers) to the adult population**
- **Employer referral (e.g. after contact with Occupational Health Services at their place of work)**
- **COVID-19 National Testing Programme; NHS registries and databases (e.g. NHS Digital)**

**Adults with either:**

- **Symptoms (within 5 days) but yet to be tested**
- **Symptoms and tested positive for SARS-CoV-2 (within 7 days)**
- **No symptoms and tested positive for SARS-CoV-2 (within 48 hours)**

**Participating Site**

**(Site with capacity for on-site and/or home Screening/Baseline visits)**

**Lead Site**

**Participant Identification Centre (PIC)**

**Participant referred to Participating Sites**

**Pre-Screening Telephone contact**

**Research team to:**

**Explain the trial and provide copy PIS/ICF**

**Ask for verbal consent to collect personal and medical information**

**Collect participant details**

**Check and/or confirm provisional eligibility**

**Check if test done or test results available**

**Arrange date, time and location for Screening/ Baseline visit**

**Day 1 - Screening/Baseline visit (Home or on-site)**

**Randomisation and start IMP**

**Day 2 to Day 6 (including Day 5 Telephone contact)**

**Day 7 Visit (Home or on-site)**

**Day 14 Visit (Home or on-site)**

**Day 28 Telephone Follow-up**

**Telephone Pre-Screening**

**Day 1 – Screening/ Baseline**

- **Research staff sees participant in self-isolation at their home or designated hospital area**
- **Check and confirm eligibility**
- **Check data collected at pre-screening**
- **Participant signs consent and recruited.**
- **Diagnostic swab *(if test yet to be done)***
- **Baseline Saliva and Blood samples collected**
- **Urine pregnancy test for women of childbearing potential**

**Day 1 to Day 6**

- **Day 1: Participant starts trial medication (witnessed 1^st^ dose)**
- **Day 1 to Day 6:**
- **Body Temperature recorded 4 times daily (twice daily on Day 1)**
- **Saliva sample collected**
- **Symptoms diary completion**
- **Day 5: Telephone contact**
- **Primary endpoint saliva sample reminder**
- **Arrange time visit for Day 7 visit and instructions for IMP intake time on Day 7**
- **AEs and SAEs check**

**Day 7 (Last dose) - Home or designated hospital area**

- **Blood samples, saliva and stool samples collected.**
- **PK samples (Pre-dose and 30-60 minutes post-dose samples)**
- **Clinical assessment**
- **AEs and SAEs check**

**Day 14 - Home or designated hospital area**

- **Blood samples collected where applicable**
- **Clinical assessment**
- **AEs and SAEs check**

**Day 28 – Telephone Follow-up**

- **Clinical assessment (AEs and SAEs check only)**
- **Questionnaire**


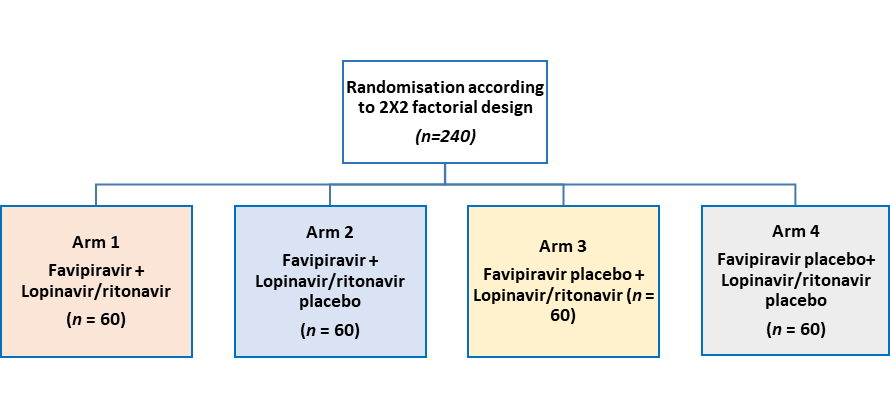


**Participants contact Research team**

**Research team contact participants**


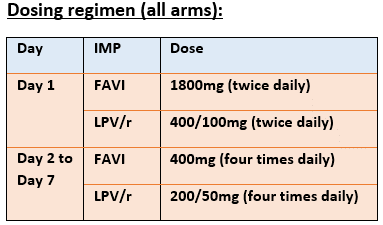


# 3 Abbreviations

| AE | Adverse Event |
| --- | --- |
| ALT | Alanine aminotransferase |
| AR | Adverse Reaction |
| AST | Aspartate aminotransferase |
| CA | Competent Authority |
| CI | Chief Investigator |
| CRF | Case Report Form |
| CTA | Clinical Trial Authorisation |
| CCTU | Comprehensive Clinical Trials Unit at UCL |
| DSUR | Development Safety Update Report |
| EC | Ethics Committee |
| EC50 | Half maximal effective concentration |
| eGFR | Estimated glomerular filtration rate |
| EU | European Union |
| GCP | Good Clinical Practice |
| IB | Investigator’s Brochure |
| ICH | International Conference on Harmonisation |
| ICH-UCL | UCL Great Ormond Street Institute of Child Health |
| ICU | Intensive Care Unit |
| IDMC | Independent Data Monitoring Committee |
| IEC | Independent Ethics Committee |
| IMP | Investigational Medicinal Product |
| IRB | Institutional Review Board |
| ITT | Intention to Treat |
| LPV/r | Lopinavir plus ritonavir |
| MHRA | Medicines and Healthcare products Regulatory Agency |
| PD | Pharmacodynamics |
| PI | Principal Investigator |
| PIS | Participant Information Sheet |
| PK | Pharmacokinetics |
| PPE | Personal Protective Equipment |
| QA | Quality Assurance |
| QC | Quality Control |
| QMMP | Quality Management and Monitoring Plan |
| R&D | Research and Development |
| SAE | Serious Adverse Event |
| SAP | Statistical Analysis Plan |
| SAR | Serious Adverse Reaction |
| SPC | Summary of Product Characteristics |
| SUSAR | Suspected Unexpected Serious Adverse Reaction |
| TMF | Trial Master File |
| TMG | Trial Management Group |
| TMT | Trial Management Team |
| ToR | Terms of Reference |
| TSC | Trial Steering Committee |
| UCL | University College London |
| ULN | Upper Limit of Normal |
| WOCBP | Women of Childbearing Potential |

# 4 Glossary

| Anosmia | Loss of the sense of smell, either total or partial |
| --- | --- |
| Cholangitis | Inflammation of the bile duct system |
| Coryza | Inflammation of the mucous membranes lining the nasal cavity, usually causing a running nose, nasal congestion and loss of smell |
| COVID-19 | Coronavirus disease caused by severe acute respiratory syndrome coronavirus 2 (SARS-CoV-2) |
| Highly effective contraception | For this trial, contraceptive methods that result in a failure rate of < 1% per year, supplemented with a barrier method (preferably male condom). This applies from the time of signing the informed consent form until 7 days after the last trial medication dose intake. |
| MERS-CoV | Middle East respiratory syndrome coronavirus (MERS-CoV) coronavirus causing previous outbreak in 2012 |
| SARS-CoV-1 | Severe acute respiratory syndrome-1 coronavirus causing previous outbreak in 2003 |
| SARS-CoV-2 | Severe acute respiratory syndrome coronavirus 2, causing COVID-19 pandemic in 2020 |
| Steatohepatitis | Type of fatty liver disease, characterised by inflammation of the liver with concurrent fat accumulation in liver |

# 5 Introduction

## 5.1 Background and Rationale

### 5.1.1 Summary

The 2020 pandemic of SARS-CoV-2 causing COVID-19 disease is an unprecedented global emergency. COVID-19 appears to be a disease with an early phase where the virus replicates, coinciding with first presentation of symptoms, followed by a later ‘inflammatory’ phase which results in severe disease in some individuals. It is known from other rapidly progressive infections such as sepsis and influenza that early treatment with antimicrobials is associated with better outcome. The hypothesis is that this holds for COVID-19 and that early antiviral treatment may prevent progression to the later phase of the disease.

The antiviral drug favipiravir is active against a broad range of viruses and has shown promising results in COVID-19 disease in two smalls Chinese studies^3^. The drug works similarly to remdesivir (another promising treatment for COVID-19), but unlike remdesivir (which is intravenous) it has a well-established and currently available oral formulation with a good safety profile. Furthermore, both Royal Free Hospital and Great Ormond Street Hospital (London) involved in this trial are amongst the only UK groups with direct clinical experience of this drug, having successfully used favipiravir to treat other viral infections.

The results of the early studies^3^ on favipiravir in COVID-19 urgently need to be confirmed or refuted in a high-quality placebo-controlled trial. However, it should also be noted that single agent oral favipiravir may not be sufficient for rapid viral eradication.

Another antiviral with potential activity in COVID-19 is the combined tablet lopinavir/ritonavir (LPV/r). This is a well-established therapy for HIV infection and lopinavir is known to have some antiviral activity against coronaviruses. Although LPV/r did not demonstrate a significant impact in COVID-19 as a single therapy in a recent study^4^ in severely unwell patients, a numerical reduction in mortality (8% versus 13%) and reduced intensive care stay was observed in patients who received the drug early. Favipiravir and LPV/r have different mechanisms of action so given together are likely to act at least additively, and possibly synergistically, providing strong rationale for a trial which examines combination therapy.

Therefore, the plan is to conduct a proof-of-principle placebo-controlled clinical trial of favipiravir plus or minus LPV/r in adults. Participants with or without symptomatic COVID-19 or tested positive will be assigned to receive favipiravir plus LPV/r or favipiravir plus LPV/r placebo or favipiravir placebo plus LPV/r or favipiravir placebo plus LPV/r placebo. The primary outcome will be the difference in the amount of virus (‘viral load’) in the upper respiratory tract after 5 days of therapy. Secondary outcomes will include hospitalisation, major morbidity and mortality, pharmacokinetics, and impact of antiviral therapy on viral genetic mutation rate.

If favipiravir, LPV/r, or favipiravir with LPV/r demonstrate important antiviral effects without significant toxicity, there will be a strong case for a larger trial in people at high risk of hospitalisation or intensive care admission, for example older patients and/or those with comorbidities and with early disease.

### 5.1.2 Detailed scientific background and rationale

**Rationale for early treatment:**

In viral infections such as influenza, it is well recognised that early antiviral therapy is associated with improved clinical outcome. During the outbreak of Severe Acute Respiratory Syndrome Coronavirus-1 (SARS-CoV-1), treatment of patients with lopinavir/ritonavir (LPV/r) plus ribavirin initiated immediately upon diagnosis led to significant mortality decreases compared to historical controls^4^. Although a rather poor-quality study, this does provide an indication that early treatment may be beneficial. Another study on LPV/r plus ribavirin showed reduced mortality and need for intubation only when therapy was given early, with late rescue treatment having no effect^5^**.** Early post-exposure prophylaxis against Middle East Respiratory Syndrome (MERS-CoV) in healthcare workers showed that LPV/r pus ribavirin reduced the incidence of infection from 28% to 0%^6^.

Two very recent pre-print papers^7,8^ on the viral dynamics of SARS-CoV-2, the cause of COVID-19, provide simulations to show that viral load peaks around 3 days after symptom onset and that antiviral therapy started later than day 4 may have limited benefit**.** Furthermore, late antiviral therapy, once viral loads are high, risks selection for drug resistance**.** Therefore, there are strong grounds to believe that if antivirals are to have a place in the treatment of SARS-CoV-2, they should be given early and, if possible, within the first 7 days of symptom onset.

**Rationale for assessment time points:**

Viral load falls rapidly and hence placebo group participants may have negative viral loads by day 7 of treatment^9^ (day 14 post symptom onset as subjects up to Day 7 post onset could be recruited in this trial). Therefore, Day 5 is the current best estimate of the time point at which drug-induced differences in viral loads are expected to be seen, and hence is the primary endpoint and time at which a formal assessment will be conducted. Given the uncertainty over viral trajectories in SARS-CoV-2 at present, daily viral loads will be taken, and viral trajectory assessed as a secondary endpoint.

**Rationale for combination therapy:**

Both recent SARS-CoV-2 modelling papers suggest that, to be effective, drugs need to provide a minimum of 65% inhibition of viral replication, with this percentage increasing to 90% or more if therapy is initiated late. Based on the in vitro EC50s of re-purposed drugs currently being trialled such as hydroxychloroquine, lopinavir and interferon, percentage inhibition using monotherapies are expected to be 33%, 66% and 18% respectively, meaning monotherapy with the currently proposed re-purposed drugs may not be effective. Whilst caution needs to be exercised in extrapolating in vitro EC50 to clinical antiviral activity, combination therapy may improve the chances of achieving sufficient viral inhibition.

Using drugs with differing modes of action is a fundamental principle of antimicrobial chemotherapy and is used across the spectrum of infectious diseases (for example in HIV, tuberculosis, multi-drug resistant bacteria and invasive fungal disease). Furthermore, the most promising efficacy of antiviral therapy in SARS-CoV-1 and MERS-CoV was with two drugs with complimentary modes of action: LPV/r plus ribavirin^4, 5, 6^**.** Therefore, there is strong reason to believe that combination therapy may be necessary, but the correct combination, with limited potential for iatrogenic harm and with a strong pharmacological rationale for their choice, must be selected.

**Pharmacological and clinical rationale for choice of interventions:**

Favipiravir

Favipiravir, manufactured by Toyama Chemical Co. (part of Fujifilm, Japan) is a novel antiviral initially developed for influenza. The drug is metabolised to a ribosyl triphosphate form which specifically inhibits the RNA dependent RNA polymerase without affecting host RNA and DNA polymerases, thereby rendering it a specific inhibitor of viral RNA synthesis. A graphical representation of the mechanism of action, as provided by Fujifilm with specific reference to influenza, is provided in Figure 1 in Appendix 1. Favipiravir has been demonstrated to have a wide spectrum of activity against RNA viruses in vitro, as summarised in Figure 2 in Appendix 1. It has been used clinically in several viral infections and in some cases, the drug has been observed to induce viral mutagenesis^10^**.** Phase I studies and pharmacokinetic studies of favipiravir have indicated increased blood levels of uric acid and elevation of liver aminotransferases in some individuals (Figure 4 in Appendix 1). Importantly, animal studies have indicated potential teratogenicity and the drug is distributed in sperm: therefore, a negative pregnancy test for all women of child-bearing potential will be required and participants instructed to use adequate contraception for the duration of the treatment and 7 days afterwards.

Favipiravir has predominantly been used in clinical trials for influenza. Trials^3^ for this indication include:

- A Phase I/II study in type A or B influenza using favipiravir doses 1800mg bd for 1 day followed by 800mg bd for 4 days (101 patients) or 2400mg/600mg/600mg for 1 day followed by 600mg tds for 4 days (82 patients) or placebo (88 patients).
- Two Phase III studies in type A or B influenza using favipiravir doses 1800mg bd for 1 day followed by 800mg bd for 4 days (301 and 526 patients respectively) or placebo (322 and 169 patients respectively).
- A global phase III study in type A or B influenza using favipiravir 1200mg/400mg for 1 day followed by 400mg bd for 4 days or oseltamivir 75mg bd for 5 days (377 and 380 patients respectively)
- A phase II study in type A or B influenza using favipiravir doses 1000mg bd for 1 day followed by 400mg bd for 4 days (88 patients) or 1200mg bd for 1 day followed by 800mg bd for 4 days (121 patients) or placebo (124 patients).

Safety data have been encouraging in these studies. Overall, the frequency of adverse events was 386/1472 (26.2%) with favipiravir versus 227/894 (25.4%) with placebo and there were no differences in the incidence of any specific adverse event between groups.

Favipiravir was also used for Ebola virus disease, especially in the JIKI trial in Guinea^11^. Doses (2400mg/2400mg/1200mg on Day 1 followed by 1200mg twice daily) were significantly higher than proposed for the current study. No drug related grade 3 or 4 clinical events were observed. In 41 of the 48 patients who survived, biochemical abnormalities of renal and liver function rapidly improved on treatment; 7 of 48 patients saw transient rises in one marker but all subsequently normalised despite continuing favipiravir. Biochemical abnormalities in patients who died were attributed to severe viral infection.

In London institutions such as Royal Free Hospital and Great Ormond Street Hospital (GOSH), favipiravir has been used as post-exposure prophylaxis for Ebola virus, for chronic norovirus infection, refractory influenza infection, astrovirus, respiratory syncytial virus and seasonal coronavirus. Occasional asymptomatic elevation of liver aminotransferase levels was observed, but no serious adverse events related to the drug. This includes in patients with immunodeficiencies who have received several months-worth of treatment.

Favipiravir is orally administered and well absorbed with close to 100% bioavailability^12^. Although higher doses have been used in Ebola, 1800 mg twice daily on day 1 followed by 800 mg twice daily is the highest dose to have been used in Phase II trials and for which safety is established, and hence is proposed in this trial. Whilst the dose in this protocol is higher than that proposed in most ongoing trials^13^, its safety has been established in previous studies and is lower than that used in Ebola. This dose should ensure plasma levels over 60 mcg/mL are achieved by the second dose and maintained in that range (**Appendix 1, Figure 3**) and the total daily dose will be split four times per day rather than twice per day (i.e., 400 mg four times per day vs 800 mg twice per day) with the aim of keeping trough levels as high as possible. Since participants will be recruited during the day, they will be asked to take both loading doses on the same day, separated by 6 hours (i.e., if the first 1800 mg dose is taken at 5pm, the next dose should be taken at 11pm). The importance of quickly achieving high antiviral levels outweighs the risk of potential toxicity and it should be noted Ebola dosing of 2400 mg separated by 8 hours has been previously used (see above).

Due to the possibility of nonlinear time dependent PK, an important secondary endpoint of this trial is to measure PK at Day 7. Assuming 60 mcg/ml can be achieved, this level is over 6-fold higher than the reported EC50 of favipiravir for SARS-CoV-2. However, since favipiravir is around 54% bound to plasma proteins, it still may only be predicted to yield 77% inhibition of viral replication.

Favipiravir has been used to treat patients with COVID-19 in two Chinese studies^14, 15^ giving some indication about the likely clinical effect. The first study^14^ was published but subsequently withdrawn for unspecified reasons. A new corrected version of this study has since been published. With this caveat, the authors had reported that favipiravir was administered (open-label) to 35 patients with COVID-19 and outcome was compared to 45 patients treated with lopinavir/ritonavir in the preceding week; all patients also received inhaled interferon and supportive care. The median time to viral clearance in the favipiravir-treated group was 4 days compared to 11 days in those who did not receive favipiravir (p<0.001). At Day 14 from the start of treatment, chest CT scans had improved in 91.4% of favipiravir-treated patients compared to 62.2% of those who did not receive favipiravir (p=0.004).

In the second study^15^, patients were consecutively recruited and randomly assigned to receive favipiravir or umifenovir (arbidol), a drug used for influenza in China; 120 participants were enrolled into each arm. In participants without severe disease, clinical recovery at 7 days was higher in the patients who received favipiravir (71.4% vs 55.9%, p=0.02). Time to resolution of fever and cough was also quicker in the favipiravir-treated group without severe illness. However, there was no significant difference in time to recovery among patients with severe disease, supporting the hypothesis that antiviral agents are best administered early in the disease course and certainly before the onset of respiratory failure.

Lopinavir/Ritonavir (LPV/r)

LPV/r is a well-established, licensed protease inhibitor therapy in routine clinical usage for the treatment and prevention of HIV/AIDS. It combines [lopinavir](https://en.wikipedia.org/wiki/Lopinavir) with a low dose of [ritonavir](https://en.wikipedia.org/wiki/Ritonavir). It is generally recommended for use in combination with other antiretrovirals. As such, it is difficult to discern side effects of an individual drug. Gastrointestinal complaints such as diarrhoea are the most common, reported by between 5% and 28% of patients depending on the study and concomitant antiretroviral medications. More serious side effects, including pancreatitis and disturbance of serum lipids, are reported but only appear to be a risk with longer term treatment.

The ritonavir component is a potent inhibitor of CYP450 liver enzymes (CYP3A4), and drug-drug interactions can be significant. Therefore, LPV/r should not be co-administered with medications that are highly dependent on CYP3A for clearance and for which elevated plasma concentrations are associated with serious and/or life-threatening events. Participants on any of these concomitant medications will be excluded from the trial. Participants with untreated or poorly controlled HIV infection will also be excluded, to prevent the risk of developing resistance, and those already receiving protease inhibitor therapy.

LPV/r has putative activity against SARS-CoV-2^16^ and of the currently tested re-purposed therapies has the highest potential benefit based on in vitro data. Furthermore, unlike (hydroxy)chloroquine, LPV/r does not cause cardiac toxicity and does not risk harmful drug-drug interactions with favipiravir.

LPV/r has been trialled as monotherapy in a recent study^4^ in severely unwell patients. There was no difference in time to clinical improvement or in 28-day mortality between treated and non-treated patients, but a numerical reduction in mortality was seen in those who initiated the medication less than 12 days from the onset of symptoms (8% versus 13%). Furthermore, reduced intensive care stay was observed in the treated group. In the absence of strong clinical pharmacology evidence to suggest otherwise, the proposal is to use the licensed adult dose of LPV/r (although the total daily dose will be divided into four times per day rather than twice per day).

Combination of favipiravir and lopinavir/Ritonavir

Favipiravir and LPV/r have different mechanisms of action so given together are likely to act at least additively. There is a possibility that the combination is synergistic since for lopinavir and ribavirin (another drug acting on RNA polymerase) *in vitro* synergy has been demonstrated with SARS-CoV-1^4^.

The preliminary results seen for favipiravir urgently need to be confirmed (or refuted) in a well conducted, placebo-controlled trial. Therefore, there is a strong rationale for choosing to trial early oral antiviral therapy with favipiravir and to investigate its combination with LPV/r.

**Rationale for study population:**

Antiviral medications are most likely to be effective when administered soon after infection. There is therefore an urgent need to study subjects who have recently developed symptoms or have recently been tested positive with or without symptoms, and who can be sampled frequently to understand changes in viral load. This cohort will allow us to collect detailed trajectory data on early disease and understand how pharmacological interventions may affect this.

## 5.2 Objectives

The objective of this trial is to assess whether early antiviral therapy with either favipiravir + LPV/r, LPV/r or favipiravir is associated with a decrease in viral load compared with placebo.

## 5.3 Trial Design

FLARE is a multi-site, UK and international, phase IIA randomised, double-blind, 2x2 factorial placebo-controlled, interventional trial.

Randomisation will be by minimisation, with the following factors: trial site, age (≤ 55 vs > 55 years old), gender, obesity (BMI <30 vs ≥30), symptomatic or asymptomatic, current smoking status (Yes = current smoker, No = ex-smoker, never smoker), ethnicity (Caucasian, other), previous COVID-19 specific vaccination (Yes = ≥ 1 dose, No = no previous covid-19 specific vaccination) and presence or absence of comorbidity (defined as diabetes, hypertension, ischaemic heart disease (including previous myocardial infarction), other heart disease (arrhythmia and valvular heart disease), asthma, COPD, other chronic respiratory disease).

Participants will be adults who have developed the early symptoms of COVID-19 within the first 5 days or tested positive for SARS-CoV-2 within the first 7 days of symptom onset, or not presenting symptoms but tested positive within the last 48 hours (date/time of test must be within 48 hours of enrolment).

Participants will be randomised 1:1:1:1 using a concealed online minimisation process into one of the following four arms:

**Arm 1**:  **Favipiravir + Lopinavir/ritonavir (LPV/r)**

Oral favipiravir at 1800 mg twice daily on Day 1, followed by 400 mg four (4) times daily from Day 2 to Day 7

**PLUS** Lopinavir/ritonavir (LPV/r) at 400mg/100 mg twice daily on Day 1, followed by 200mg/50mg four (4) times daily from Day 2 to Day 7

**Arm 2**: **Favipiravir + Lopinavir/ritonavir (LPV/r) placebo**

Oral favipiravir at 1800 mg twice daily on Day 1, followed by 400 mg four (4) times daily from Day 2 to Day 7

**PLUS** Lopinavir/ritonavir (LPV/r) matched placebo at 400mg/100mg twice daily on Day 1, followed by 200mg/50mg four (4) times daily from Day 2 to Day 7.

**Arm 3**: **Favipiravir placebo + Lopinavir/ritonavir (LPV/r)**

Oral favipiravir matched placebo at 1800 mg twice daily on Day 1, by 400 mg four (4) times daily from Day 2 to Day 7

**PLUS** Lopinavir/ritonavir (LPV/r) at 400mg/100mg twice daily on Day 1, followed by 200mg/50mg four (4) times daily from Day 2 to Day 7.

**Arm 4**: **Favipiravir placebo + Lopinavir/ritonavir (LPV/r) placebo**

Oral favipiravir matched placebo at 1800 mg twice daily on Day 1, by 400 mg four (4) times daily from Day 2 to Day 7

**PLUS** Lopinavir/ritonavir (LPV/r) matched placebo at 400mg/100mg twice daily on Day 1, followed by 200mg/50mg four (4) times daily from Day 2 to Day 7.

# 6 Methods

## 6.1 Site Selection

The trial sponsor has overall responsibility for site and investigator selection and has delegated this role to CCTU.

### 6.1.1 Study Setting

It is anticipated that the trial will take place at the Royal Free Hospital London (Royal Free London NHS Foundation Trust) - Lead site in the first instance.

Additional sites and participant identification centres (PIC) will be added if necessary, and up to four sites internationally including sites in Malaysia and Kenya in the first instance.

Screening/Baseline trial assessments will be conducted on-site (in designated hospital area) or at participants’ homes. Once participants have completed their self-isolation period, trial assessments will be conducted at sites (in a designated hospital area).

### *6.1.2 Site/Investigator Eligibility Criteria*

Once a site has been assessed as being suitable to participate in the trial, the trial team will provide them with a copy of this protocol and relevant trial documents.

To participate in the FLARE trial, Investigators and trial sites must fulfil a set of criteria that have been agreed by the FLARE Trial Management Group (TMG) and that are defined below.

Eligibility criteria:

- A named clinician is willing and appropriate to take Principal Investigator responsibility
- Suitably trained staff are available to recruit participants, collect data and biospecimen samples
- The site should be able to store, prepare and dispense IMP appropriately.

#### 6.1.2.1 Principal Investigator’s (PI) Qualifications and Agreements

The investigator(s) must be willing to comply with the trial protocol (confirming their specific roles and responsibilities relating to the trial, and that their site is willing and able to comply with the requirements of the trial) as agreed in the site agreement. This includes confirmation of appropriate qualifications, by provision of a CV, familiarity with the appropriate use of any investigational products, agreement to comply with the principles of GCP, to permit monitoring and audit as necessary at the site, and to maintain documented evidence of all staff at the site who have been delegated significant trial related duties.

**The investigator(s) at International sites must be willing to comply with their protocol country- specific addendum and their country specific Good Clinical Practice.**

**For international sites, country-specific procedures will be implemented in accordance with local IRB/IEC and national regulatory authority requirements.**

**See country-specific addendum for further details on site-specific procedures and documents.**

#### 6.1.2.2 Resourcing at site

The investigator(s) should be able to demonstrate a potential for recruiting the required number of suitable participants within the agreed recruitment period (i.e., the investigator(s) regularly treat(s) the target population). They should also have an adequate number of qualified staff and facilities available for the foreseen duration of the trial to enable them to conduct the trial properly and safely.

Sites will be expected to complete a delegation of responsibilities log and provide staff contact details.

Sites should have sufficient data management resources to allow prompt data return to CCTU.

## 6.2 Site approval and activation

On receipt of confirmation of capacity and capability, a signed site agreement, approved delegation of responsibilities log and staff contact details, the Trial Manager or delegate will notify the Principal Investigator (PI) in writing of the plans for site activation. Sites will not be permitted to recruit any participants until a letter for activation has been issued. The Trial Manager or delegate will be responsible for issuing this after a green light to recruit process has been completed.

The site must conduct the trial in compliance with the protocol as agreed by the Sponsor, by the MHRA and which was given favourable opinion by a Research Ethics Committee (REC) and the Health Research Authority (HRA). The PI or delegate must document and explain any deviation from the approved protocol and communicate this to the trial team at the CCTU.

**International sites must conduct the trial in compliance with country-specific procedures and in accordance with local IRB/IEC and national regulatory authority requirements.**

**See country-specific addendum for further details on site-specific procedures and documents.**

A list of activated sites may be obtained from the Trial Manager.

## 6.3 Participants

### 6.3.1 Eligibility Criteria

Any adults who have developed the early symptoms of COVID-19 within the first 5 days or tested positive for SARS-CoV-2 within the first 7 days of symptom onset, or not presenting with symptoms but tested positive within the last 48 hours (date/time of test must be within 48 hours of enrolment).

#### 6.3.1.1 Participant selection

In the UK, recruitment will be done:

1. Via targeted advertising to the adult population
2. Via participant self-referral
3. Via employer referral (e.g., Occupational Health Services)
4. Via COVID-19 National Testing Programme
5. Via NHS registries and databases (e.g., NHS Digital)

For international sites, each country will adopt an approach that is appropriate for enrolment according to local conditions, and one that is in compliance with its IRB/IEC and national regulatory authorities’ guidelines.

See country-specific addendum for further details on site-specific procedures for participant selection.

There will be **no exceptions** (waivers) to eligibility requirements at the time of randomisation. Questions about eligibility criteria should be addressed **prior** to attempting to randomise the participant.

The eligibility criteria for this trial have been carefully considered and are the standards used to ensure that only medically appropriate participants are enrolled. Participants not meeting the criteria should not be enrolled into the trial for their safety and to ensure that the trial results can be appropriately used to make future treatment decisions for other people with similar disease or conditions. It is therefore vital that exceptions are not made to these eligibility criteria.

Participants will be considered eligible for enrolment in this trial if they fulfil all the inclusion criteria and none of the exclusion criteria as defined below.

#### 6.3.1.2 Participant Inclusion Criteria

Inclusion criteria are as follows:

1. Any adult with the following:

- Symptoms compatible with COVID-19 disease (Fever >37.8^o^C on at least one occasion AND either cough and/ or anosmia) within the first 5 days of symptom onset (date/time of enrolment must be within the first 5 days of symptom onset)
- **OR** ANY symptoms compatible with COVID-19 disease (may include, but are not limited to fever, cough, shortness of breath, malaise, myalgia, headache, coryza) and tested positive for SARS-CoV-2 within the first 7 days of symptom onset (date/time of enrolment must be within the first 7 days of symptom onset)
- **OR** no symptoms but tested positive for SARS-CoV-2 within the last 48 hours (date/time of test must be within 48 hours of enrolment)

1. Male or female aged 18 years to 70 years old inclusive at screening
2. Willing and able to take daily saliva samples
3. Able to provide full informed consent and willing to comply with trial-related procedures

#### 6.3.1.3 Participant Exclusion Criteria

Exclusion criteria are as follows:

1. Known hypersensitivity to any of the active ingredients or excipients in favipiravir and matched placebo, and in lopinavir/ritonavir and matched placebo (**See Appendix 2**)
2. Chronic liver disease at screening (known cirrhosis of any aetiology, chronic hepatitis (e.g. autoimmune, viral, steatohepatitis), cholangitis or any known elevation of liver aminotransferases with AST or ALT > 3 X ULN)*
3. Chronic kidney disease (stage 3 or beyond) at screening: eGFR < 60 ml/min/1.73m^2^*
4. HIV infection, if untreated, detectable viral load or on protease inhibitor therapy
5. Any clinical condition which the investigator considers would make the participant unsuitable for the trial
6. Concomitant medications known to interact with favipiravir and matched placebo, and with lopinavir/ritonavir and matched placebo, and carry risk of toxicity for the participant (**See Appendix 3**)
7. Current severe illness requiring hospitalisation^**^
8. Pregnancy and/ or breastfeeding
9. Eligible female participants of childbearing potential and male participants with a partner of childbearing potential not willing to use highly effective contraceptive measures during the trial and within the time point specified following last trial treatment dose.
10. Participants enrolled in any other interventional drug or vaccine trial (co-enrolment in observational studies is acceptable).***

* Considering the importance of early treatment of COVID-19 to impact viral load, the absence of chronic liver/ kidney disease will be confirmed verbally by the participant during pre-screening and Screening/Baseline visit. Safety blood samples will be collected at Screening/Baseline visit (Day 1) and test results will be examined as soon as they become available within 24 hours.

**Hospitalisation for isolation or infection control reasons will not considered an exclusion for this trial for international sites.

***Participants that have received the COVID-19 vaccine can be included in this trial.

#### 6.3.1.4 Women of Child Bearing Potential (WOCBP)

Female participants of childbearing potential must not become pregnant and must use contraceptive methods with a failure rate of < 1% (highly effective contraceptive methods), supplemented with a barrier method (preferably male condom):

- during therapy
- **and for up to 7 days after stopping the therapy**, unless abstinence is the chosen method of contraception. Abstinence is acceptable only as total abstinence from intercourse with male partners: when this is in line with the preferred and usual lifestyle of the participant.

WOCBP excludes women who are postmenopausal or permanently sterilised, e.g., tubal ligation, hysterectomy, bilateral salpingectomy or bilateral oophorectomy.

WOCBP participants should agree to remain consistent and to correctly use highly effective contraceptive methods that result in a failure rate of < 1% per year, supplemented with a barrier method (preferably male condom) during their participation in the trial. Contraception methods with low user dependency should preferably be used^17^.

This must be clearly documented in the CRF prior to their randomisation.

Highly effective contraceptive methods include:

- Combined (oestrogen and progestogen containing) hormonal contraception associated with inhibition of ovulation ***(only if hormonal contraception is established at least 1 month prior to randomisation. WOCBP participants should be warned of the possibility of an additional prothrombotic risk if combined hormonal contraception is taken contemporary with the trial treatment)***
  - Oral, intravaginal, transdermal progestogen-only hormonal contraception associated with inhibition of ovulation
  - Oral or injectable
  - Implantable- (*low user dependency)*
- Intrauterine device (IUD) – (*low user dependency)*
- Intrauterine hormone-releasing system (IUS) – (*low user dependency)*
- Bilateral tubal occlusion – (*low user dependency)*
- Male partner sterilisation (vasectomy with documentation of azoospermia) prior to the female participants’ entry into the study, and this male is the sole partner for that participant (*low user dependency)*
- Total sexual abstinence

Periodic abstinence (for example, calendar, ovulation, sympto-thermal and post ovulation methods) and the withdrawal method are not considered acceptable methods of contraception.

**Pregnancy testing:**

**Screening/Baseline visit:** Following written consent, women of childbearing potential will undergo a urine pregnancy test **prior** to randomisation at Screening/Baseline visit (Day 1). The results of this pregnancy test must be available prior to dispensing the IMPs and should be clearly documented on the CRF.

**After last day of treatment**: a urine pregnancy test must be obtained for WOCBP at least 7 days after their last dose of IMPs (last day of treatment).

#### 6.3.1.5 Male Participants

Due to probable teratogenicity of favipiravir, sexually active male participants should be required to use a condom as precautionary measure **and** advised that their female partners of child bearing potential use highly effective contraception methods during treatment and for at least 7 days after cessation of IMPs.

Investigators should inform all male participants of reproductive potential, including those planning to have children, of the potential risks and document this in the CRF.

#### 6.3.1.6 Eligibility Criteria for Individuals Performing the Interventions

In the UK, registered research nurse or research assistant, any other delegated individual or doctor trained in the FLARE trial protocol, GCP and use of appropriate PPE as per Public Health England Guidance (**See** **Appendix 5**). Research staff attending the participant’s home must wear appropriate PPE in advance of entering the house, during the visits and until after they leave the premises. As in all situations during the pandemic, guidance on [infection control](https://www.gov.uk/government/publications/wuhan-novel-coronavirus-infection-prevention-and-control) should be followed, including handwashing. As a minimum, single use disposable plastic aprons, surgical mask and gloves must be worn at all times.

Home-visiting research staff should ensure that ‘home visit’ bags contain necessary additional PPE and clinical waste bags. All equipment used at visits should then be appropriately decontaminated when returning to the practice.

**International sites must conduct the trial in compliance with country-specific procedures and in accordance with local IRB/IEC and national regulatory authority requirements.**

See country-specific addend for further details on site-specific procedures.

Each member of the Trial Team at each site will have their roles within the trial, as delegated by the PI and documented in the FLARE trial site delegation log. The FLARE trial team at UCL CCTU will collect CVs and current GCP training certificates for all staff working on the trial to document their qualifications and relevant experience. Protocol-specific training will be provided to participating sites prior to site activation. New site staff who start working on the trial after site activation occurs, will be provided with protocol specific training prior to performing trial related procedures.

#### 6.3.1.7 Co-enrolment Guidance

The FLARE trial team at sites will be responsible for ascertaining whether the patient is currently taking part in a clinical trial. Participants may be enrolled in other observational studies but not in interventional drugs or vaccine trials.

#### 6.3.1.8 Screening Procedures and Pre-randomisation Investigations

Potential participants will be identified from several sources as it is essential that participants are treated as soon as possible. Potential participants will be provided with trial information and a full participant information sheet (PIS) by email. When a participant has had time to review the PIS a telephone screening will be undertaken. This will allow rapid, efficient selection of participants most likely to pass the Screening/Baseline visit and represents the best available strategy for practical screening in this trial. The telephone conversation will be conducted by delegated research staff. Participants will give verbal consent to disclose basic medical information to check for contraindications to treatment (e.g., Chronic liver and kidney disease, concomitant medications known to interact with trial medications) and to check provisional eligibility.

The following data collected during the pre-screening telephone contact: study site, age (≤ 55 vs > 55 years old), gender, obesity (BMI <30 vs ≥30), symptomatic or asymptomatic, current smoking status (Yes = current smoker, No = ex-smoker, never smoker), ethnicity (Caucasian, other), previous COVID-19 specific vaccination (Yes/No; vaccine type; number of doses; and date(s) of dose(s)) and presence or absence of comorbidity (defined as diabetes, hypertension, ischaemic heart disease (including previous myocardial infarction), other heart disease (arrhythmia and valvular heart disease), asthma, COPD, other chronic respiratory disease, will be used for the purposes of registration and IMP kit pre-allocation. Considering the importance of starting therapy immediately upon entering this trial, IMP kit codes will be pre-allocated to the participants ahead of the Screening/Baseline visit on Day 1, when they will be fully consented before randomisation can be completed. No IMP kit will be given to the participants until their eligibility has been confirmed. Participants will be considered fully randomised when they ingest their first dose of IMP.

If they decide to participate, an on-site (in designated hospital area) or a home Screening/Baseline visit will be arranged where participants will be able to ask any further questions about the trial and full informed consent will be taken after eligibility by all the criteria confirmed.

Where a participant requests to speak with a physician from the trial team the consent process will not be completed until the participant had spoken to the physician and had all their questions answered to their satisfaction.

Written informed consent to enter and be randomised into the trial must be obtained from participants, after explanation of the aims, methods, benefits and potential hazards of the trial and **before** any trial-specific procedures are performed or any samples are taken for the trial. If the delegated site staff taking informed consent is not a medically qualified investigator, they will have a conversation (via teleconference) with the site principal investigator or delegate while they are still at the participant’s home: the principal investigator or delegate will confirm eligibility and consent, and approve each participant’s enrolment. The conversation, name of the principal investigator or delegate and actions taken will be documented on the Screening/Baseline CRF. Where Screening/Baseline visit is taking place at site (in designated hospital area), delegated site staff taking informed consent will follow the same procedure described above and eligibility and consent will be confirmed by the site principal investigator or delegate in clinic.

The only procedures that may be performed in advance of written informed consent being obtained is the collection of data during the pre-screening telephone contact when participant’s personal contact details (including home address) and verbal confirmation of interest will be obtained and documented, and IMP kit pre-allocation. Trial staff will record all telephone conversations in the source documents.

**For International sites, screening procedures and pre-randomisation investigations must be conducted in compliance with country-specific procedures and in accordance with local IRB/IEC and national regulatory authority requirements.**

See country-specific addendum for further details on site-specific procedures.

**Screening/Baseline Visit (Day 1)**

***On-site (in designated hospital area) or at participants’ home***

Delegated site staff will visit participants at their home or on-site (in a designated hospital area). After they have been given sufficient time to ask questions and provided written consent, participants will undergo final assessment for eligibility (including a urine pregnancy test for women of childbearing potential) and will be recruited to the trial. A baseline saliva sample for virological analysis and baseline blood samples will be taken. A baseline diagnostic nose and throat swab will be taken if the participant hasn’t been tested for COVID-19 yet.

If they are eligible, participants will be randomised and provided with a trial medication kit, symptoms diary, containers for daily saliva samples collection, one stool collection container, a thermometer and instructions for taking their medications, collecting samples and recording daily body temperature readings.

Participants will be considered fully randomised once they take their first trial medication dose, witnessed by the delegated site staff and they meet all inclusion criteria following results from safety bloods collected at Screening/Baseline visit. Arrangements will be made for their next trial visits.

If participants are deemed ineligible following one of the above assessments, data will not be collected for these participants.

The reason(s) for ineligibility will be explained to participants and any questions they have will be answered. They will be thanked for their participation and any relevant information from this will be recorded on the CRF.

If participants are deemed ineligible following results from safety bloods collected at Screening/Baseline visit, a delegated site research staff will contact the participant (within 6 hours of results receipt) to explain the reason(s) for ineligibility and instruct them to interrupt the therapy immediately. The research staff will enquire on any adverse reactions and side effects that the participant might have experienced since starting the trial medications and record details on the Screening/Baseline CRF. In order to ensure participant’s safety, arrangements for immediate collection of the medication kit from the participant’s home will be made (within 24 hours), and a pills count will be performed on receipt of the medication kit at site.

**For International sites, Screening/Baseline Visit (Day 1) must be conducted in compliance with country-specific procedures and in accordance with local IRB/IEC and national regulatory authority requirements.**

See country-specific addendum for further details on site-specific procedures.

# 6.4 Interventions

The interventions for this trial are favipiravir plus LPV/r or favipiravir plus LPV/r matched placebo or favipiravir matched placebo plus LPV/r or matched placebo of both drugs for 7 days.

## 6.4.1. Products and Treatment Schedule

## 6.4.1.1 Packaging, labelling and QP release

RenaClinical Limited will be packaging, labelling, blinding and QP release all the medications for use in this trial. The trial medication will be delivered to Pharmacy, who will be responsible for local storage, dispensing, accountability and return of trial drugs.

RenaClinical Limited will be sourcing active Lopinavir/ritonavir, manufacturing Lopinavir/ritonavir matched placebo.

RenaClinical Limited will be also performing packaging, labelling, blinding and QP release of favipiravir tablets and matched placebo received from FUJIFILM Toyama Chemical Co., Ltd.

The trial medications will be packaged for clinical trial use in kits containing the adequate number of units required for the treatment period (7 days) and labelled in compliance with Annex 13 of GMP.

Each kit will contain:

- 1 bottle of favipiravir or placebo (of 66 tablets)

**AND**

- 1 bottle of Lopinavir/ritonavir or placebo (of 28 tablets)

The treatment, packaging and labelling will be identical in appearance and treatment will be allocated using blinded kit codes (see 6.9.1.3).

## 6.4.1.2 Favipiravir and placebo

Favipiravir (Avigan®) 200 mg tablets and matched placebo will be provided by FUJIFILM Toyama Chemical Co., Ltd.

On Day 1, 9 tablets (1800 mg) will be taken twice separated by at least 6 hours (e.g., if first dose is at 5pm the second dose should be at 11pm), followed by 2 tablets (400 mg) four (4) times daily from Day 2 to Day 7.

## 6.4.1.3 Lopinavir/ritonavir and placebo

Lopinavir/ritonavir 200 mg/50 mg tablets will be sourced by RenaClinical Limited and matched placebo manufactured by the same company.

On Day 1, 2 tablets (400 mg/100 mg) will be taken twice separated by at least 6 hours (e.g., if first dose is at 5pm the second dose should be at 11pm), followed by 1 tablet (200mg/50mg) four (4) times daily from Day 2 to Day 7.

Detailed information with regard to the labelling, administration, storage etc. will be provided in the FLARE IMP Management Plan.

Treatment dosing regimen is summarised in **Table 1** below:

| **Arm** | **Total daily dosage** | **Regimen** | **Total No of tablets daily** |
| --- | --- | --- | --- |
| **FAVI active + LPV/r active**  **OR**  **FAVI active + LPV/r placebo**  **OR**  **FAVI placebo + LPV/r active**  **OR**  **FAVI placebo + LPV/r placebo** | **Day 1:**  FAVI = **3600mg**  **PLUS**  LPV/r = **800/200mg** | **FAVI**:  1^st^ Dose: 1800mg (9 tablets)  2^nd^ Dose: 1800mg (9 tablets)  **PLUS**  **LPV/r:**  1^st^ Dose: 400/100mg (2 tablets)  2^nd^ Dose: 400/100mg (2 tablets) | **Day 1:**  FAVI = 18 tablets  **PLUS**  LPV/r = 4 tablets |
|  | **Day 2 to Day 7:**  FAVI = **1600mg**  **PLUS**  LPV/r = **800/200mg** | **FAVI**:  1^st^ Dose: 400mg (2 tablets)  2^nd^ Dose: 400mg (2 tablets)  3^rd^ Dose: 400mg (2 tablets)  4^th^ Dose: 400mg (2 tablets)  **PLUS**  **LPV/r:**  1^st^ Dose: 200/50mg (1 tablet)  2^nd^ Dose: 200/50mg (1 tablet)  3^rd^ Dose: 200/50mg (1 tablet)  4^th^ Dose: 200/50mg (1 tablet) | **Day 2 to Day 7:**  FAVI = 8 tablets  **PLUS**  LPV/r = 4 tablets |

**Table 1. FLARE Trial dosing regimen**

**Follow-up Visits (Days 7, 14 and 28) and Day 5 Telephone contact**

Following randomisation at Screening/Baseline (Day 1), participants will take trial medications for 7 days. During this period, they will collect a daily saliva sample and complete a symptoms diary including four daily temperature measurements (except for Day 1 where only two temperature measurements will be recorded, and Day 7 where one or two temperature measurements will be recorded depending on the schedule time of Day 7 visit). The last dose will be taken at Day 7 (4^th^ dose on Day 7).

**For International sites, follow-up Visits (Days 7, 14 and 28) and Day 5 will be conducted in compliance with country-specific procedures and in accordance with local IRB/IEC and national regulatory authority requirements.**

See country-specific addendum for further details on site-specific procedures.

Day 5

At Day 5, participants will receive a telephone call from the research team to ensure that participants collect their daily saliva sample, arrange time for Day 7 visit (PK sampling), to provide instructions for dose intake on Day 7 and to enquire about any relevant clinical events (AEs and SAEs) that may require further clinical assessment.

**In order to accommodate sites where the research staff do not work during weekends and where a participant is randomised on a Tuesday or a Wednesday, the Day 5 telephone call may be performed on the Friday of that week.**

Day 7

At Day 7, participants will be assessed by the trial team, undergo blood tests for toxicity and pharmacokinetic assessment and provide a stool sample.

**In order to accommodate sites where the research staff do not work during weekends and where a participant is randomised on a Monday, a trial visit allowance of + 1 day will be permitted. Pharmacokinetics samples will not be collected for these participants as the last dose of IMP will be taken at Day 7 (4^th^ dose on Day 7).**

Day 14

**Where no significantly abnormal results for safety blood tests taken at Day 7 are observed, participants will not undergo blood tests at the Day 14 visit.** The research team will enquire about any relevant clinical events (AEs and SAEs) that may require further clinical assessment. Female participants of childbearing potential will attend the Day 14 visit in order to undergo urine pregnancy testing.

**A trial visit allowance of +3 days will be permitted for the Day 14 visit.**

Day 28

Participants will have a telephone follow up three (3) weeks after their last day of treatment (Day 7). The research team will enquire about any relevant clinical events that may require further clinical assessment and collect further information through a questionnaire. A trial visit allowance of +/- 3 days will be permitted for the Day 28 call.

**Please refer to section 6.7 - Participant Timeline for details of the trial assessments performed at each visit.**

## 6.4.1.4 Dose modifications, interruptions and discontinuations

No dose modifications will be permitted, there will be no planned interruptions to IMP administration. If participants suffer intolerable side effects or severe intercurrent illness, medication can be discontinued, and the study team informed at the earliest opportunity.

## 6.4.2 Dispensing

As favipiravir and lopinavir/ritonavir are not licensed for treating COVID-19, their use in this trial will be off-label. Prescribing must only be conducted by members of the research team who have been delegated this task (prescribers) and appear on the Trial Delegation Log and have suitable training, qualifications and experience.

Only one dispensing episode will occur at Screening/Baseline Visit (Day 1) on receipt of a completed Trial Prescription, and participants will be provided with comprehensive instructions on the dosing regimen.

**Home visits**

The IMP kits will be collected from pharmacy by the research staff on behalf of the participants ahead of the visit. The IMP kits will be placed in an insulated envelope with a single use temperature logger at the moment of collection from Pharmacy, and a first temperature reading recorded by the research staff. A second reading will be recorded once the research staff arrives at the participant’s home. A chain of custody form will be completed by the research staff collecting the IMP and signed by both pharmacy and the research staff. The Chain of Custody form should be completed only if the participant has their Screening/Baseline visit at home.

**On-site visits**

For Screening/Baseline visits taking place on-site (in designated hospital area), the IMP kits will be collected from pharmacy by the research staff on behalf of the participants ahead of the visit and dispensed directly to the participants during their visit.

Detailed information with regard to the labelling, administration and storage will be provided in the FLARE IMP Management Plan.

**For International sites, dispensing will be in compliance with country-specific procedures and in accordance with local IRB/IEC and national regulatory authority requirements.**

See country-specific addendum for further details on site-specific procedures.

## 6.4.3 Accountability

The site pharmacist will be delegated oversight of IMP supplies. Full IMP Accountability records will be maintained at the site pharmacy. Empty bottles and unused medication will be requested to be returned upon discontinuation of trial medication and destroyed or disposed of by the pharmacy on confirmation by the Sponsor. The CCTU will request copies of accountability logs and confirmation of destruction/disposal. Further details may be found in the FLARE IMP Management Plan.

For sites where empty bottles and participant’s unused medication cannot be destroyed or disposed by pharmacy as part of the site local policy for COVID-19 trials, the research team will be responsible for the disposal of the IMP and for maintaining records of the IMP accountability. The CCTU will request copies of Participant Returns Accountability and Disposal Log to ensure IMP chain of custody.

## 6.4.4 Concomitant Care

All medications (except those that are contraindicated according to the Lopinavir/ritonavir SPC and Favipiravir IB or mentioned in **Appendix 3**) which the trial investigator responsible for the participant’s care feels are clinically appropriate, are permitted in the trial.

Medications which are not permitted in the trial or which should be used with care are listed in **Appendix 3.**

If a participant is hospitalised during the trial period they will be encouraged where possible to continue taking trial medication, taking saliva samples, and recording temperature, but any urgent medical treatment required will be permitted.

## 6.4.5 Overdose of Trial Medication

If a participant reports overdose of trial medication, they will be advised to seek medical help. Since only 7 days of supplies are provided and both drugs have reasonably wide therapeutic indices the consequence of overdose are thought to be limited.

## 6.4.6 Protocol Treatment Discontinuation

In consenting to the trial, participants are consenting to trial treatments, trial follow-up and data collection. However, an individual participant may stop treatment early or be stopped early for any of the following reasons:

- Unacceptable treatment toxicity or adverse event
- Inter-current illness that prevents further treatment
- Any change in the participant’s condition that in the clinician’s opinion justifies the discontinuation of treatment
- Participant no longer wants to continue taking the trial medications
- Withdrawing from the trial by the participant

As participation in the trial is entirely voluntary, the participant may choose to discontinue trial treatment at any time without penalty or loss of benefits to which they would otherwise be entitled. Although not obliged to give a reason for discontinuing their trial treatment, a reasonable effort should be made to establish this reason, whilst remaining fully respectful of the participant’s rights.

Participants who discontinue protocol treatment, for any of the above reasons, should remain in the trial and undergo their remaining visits for the purpose of follow up and data analysis.

## 6.5 Sample Management

### 6.5.1 Schedule of samples

The following laboratory samples will be collected during the trial:

| **Sample** | **Day 1** | **Days 2 - 6** | **Day 7** | **Day 14^[[1]](#footnote-2)^** |
| --- | --- | --- | --- | --- |
| Blood samples | - Full blood count (4mL) - Urea & electrolytes, liver function tests, uric acid (5mL) - Serum for storage (10mL) |  | - Full blood count (4mL) - Urea & electrolytes, liver function tests, uric acid (5mL) - Serum for storage (10mL) - Plasma^[[2]](#footnote-3)^ Pharmacokinetic samples (2x2mL) | - Full blood count (4mL) - Urea & electrolytes, liver function tests, uric acid (5mL) |
| Nasopharyngeal swab (*Only if COVID-19 testing not done yet)* | - Diagnostic sample |  |  |  |
| Saliva | Sample for viral load measurement / viral sequencing | Sample for viral load measurement / viral sequencing taken daily [self-sampled] | Sample for viral load measurement / viral sequencing |  |
| *Stool samples |  |  | Sample for viral load measurement / viral sequencing |  |

***For International sites, stool samples will not be collected.**

See country-specific addendum for further details on site-specific procedures.

#### 6.5.1.1 Pharmacokinetic sampling

**Sample timing:** Samples will be drawn for pharmacokinetics on Day 7. Participants will be telephoned on Day 5 by the trial site team to arrange the Day 7 visit time.

*If Day 7 visit scheduled for the morning (before 12:00):* Participants will be instructed to take the morning dose and record the time to the nearest minute of that dose. The second dose of the day should not be taken until after the study visit. A blood sample will be drawn for PK sampling during the visit, and time to the nearest minute recorded. The second dose of the day will then be taken and the time to the nearest minute recorded. A further blood sample will be drawn after 30 to 60 minutes with time to the nearest minute recorded

*If Day 7 visit scheduled for the afternoon (after 12:00):* Participants will be instructed to take the morning dose (first dose of the day) and the lunchtime dose (second dose of the day) which should be taken at 12:00. Participants will be asked to record the time to the nearest minute of the second dose of the day. A blood sample will be drawn for PK sampling during the visit, and time to the nearest minute recorded. The afternoon dose (third dose of the day) will then be taken and the time to the nearest minute recorded. A further blood sample will be drawn after 30 to 60 minutes with time to the nearest minute recorded

**Sample collection:** If sampling from a newly inserted venous cannula, 2 mL of blood should be drawn and discarded. Then 2mL of blood should be collected into a plasma sampling tube, with time to the nearest minute and date recorded along with the participant ID. The next scheduled dose should then immediately be taken with time to the nearest minute recorded. A further 2mL of blood should be collected into a plasma sampling tube, with time to the nearest minute and date recorded along with the participant ID 30 to 60 minutes later.

### 6.5.2 Processing of samples

- Safety bloods (Full blood count, urea and electrolytes, liver function tests and uric acid) – sent to Great Ormond Street Hospital NHS diagnostic laboratory for standard assays, or will be analysed locally where applicable and as agreed with the Sponsor prior to site activation.
- Serum for storage – sent to Royal Free Hospital Centre for Clinical Microbiology (CCM) and processed by the laboratory staff: sample centrifuged and supernatant stored at -80^o^C
- Diagnostic nasopharyngeal swab – sent to Great Ormond Street Hospital NHS diagnostic laboratory for standard PCR assay for SARS-CoV-2, or will be analysed locally where applicable and as agreed with the Sponsor prior to site activation.
- Saliva and stool samples for viral load measurement and viral sequencing – sent to the Microbiology Department at Great Ormond Street Hospital for extraction, storage and subsequent qPCR with a locally developed assay and sequencing.
- Pharmacokinetic samples: sample tubes should be returned to the Great Ormond Street Hospital NHS diagnostic laboratory on the day of collection (Day 7). Plasma should be extracted and placed into a 2mL tube. This tube should then be:
- Centrifuged (3000 rpm, 15 min) and plasma extracted
- Virus inactivated by adding 1:4 (plasma:ethanol) i.e. 4mL of ethanol for each 1 mL of plasma
- Centrifuged (3000 rpm, 15 min) and supernatant extracted
- Supernatant frozen and stored at or below -20 °C until analysis by third party laboratory (bioanalytical contractors of FUJIFILM Toyama Chemical Co., Ltd in Japan).

Detailed information with regard to the samples collection, labelling, storage and processing will be provided in the FLARE Samples Management Plan.

**For international sites, country-specific procedures will be implemented in accordance with local IRB/IEC and national regulatory authority requirements.**

**See country-specific addendum for further details on site-specific procedures and documents.**

## 6.6 Outcomes

### 6.6.1 Primary Outcomes

The primary outcome is the upper respiratory tract viral load at Day 5.

Method of measurement: quantitative polymerase chain reaction (PCR) performed on saliva samples.

### 6.6.2 Secondary Outcomes

The secondary outcomes are the following:

- percentage of participants with undetectable upper respiratory tract viral load after 5 days of therapy

Method of measurement: quantitative polymerase chain reaction (PCR) performed on saliva samples

- proportion of participants with undetectable stool viral load after 7 days of therapy *(not applicable for international sites as stools samples will not be collected internationally)*

Method of measurement: PCR performed on stool samples

- rate of decrease in upper respiratory tract viral load during 7 days of therapy

Method of measurement: PCR performed on daily saliva samples

- duration of fever following commencement of medication

Methods of measurement: daily body temperature records between Day 1 and Day 7 post-randomisation

- proportion of participants with hepatotoxicity after 7 days of therapy

Method of measurement: standard diagnostic laboratory assays for liver transaminases, alkaline phosphatase and bilirubin

- proportion of participants with other medication-related toxicity after 7 days of therapy and 14 days post-randomisation

Methods of measurement: determination of medication-related adverse events by investigators

- Proportion of participants admitted to hospital with COVID-19 related illness

Methods of measurement: participant self-report, review of hospital records and discharge summaries

- Proportion of participants admitted to ICU with COVID-19 related illness

Methods of measurement: participant self-report, review of hospital records and discharge summaries

- Proportion of participants who have died with COVID-19 related illness

Methods of measurement: next of kin report, review of hospital records and discharge summaries

- pharmacokinetic and pharmacodynamic analysis of favipiravir

Method of measurement: assay of favipiravir levels in plasma at Day 7 of therapy. All participants from each arm will provide a pre-dose trough sample and a post-dose (30 to 60 min) sample on Day 7 of therapy. A nonlinear mixed effects model will be fitted jointly to favipiravir pharmacokinetic and viral load (pharmacodynamic) data.

The model will estimate the following primary PK parameters:

PK: Clearance (CL), Volume of distribution (V), Absorption rate constant (Ka)

From which the following secondary parameters will be derived:

Maximum concentration (Cmax),

Time to maximum concentration (Tmax),

Elimination rate constant (Ke),

Area Under the Curve extrapolated to infinity (AUC (0-inf))

The model will also estimate the following pharmacodynamic parameters:

Rate of viral load decline (delta),

Maximum increase in viral load under drug treatment (Emax),

Concentration to achieve half the maximum possible effect (EC50)

- Exploratory: proportion of participants with deleterious or resistance-conferring mutations in SARS-CoV-2

Method of measurement: deep sequencing of virus and bioinformatic analysis.

## Participant Timeline

For international sites, country-specific procedures will be implemented in accordance with local IRB/IEC and national regulatory authority requirements. See country-specific addendum for further details on site-specific participant timeline.

| TRIAL PERIOD | ENROLLMENT | RANDOMISATION | POST-RANDOMISATION | | | FOLLOW-UP |
| --- | --- | --- | --- | --- | --- | --- |
| VISIT | Pre-screening^[[3]](#footnote-4)^ | Screening/ Baseline | Between visits^[[4]](#footnote-5)^ | Day 7 | Day 14 | Day 28 |
| Time point:^[[5]](#footnote-6)^ |  | Day 1 | Day 1 to Day 6 | Day 7 | Day 14 | Day 28 |
| Visit number | 0 | 1 |  | 2 | 3 | 4 |
| Type of contact | 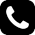 | Home visit/Clinic^[[6]](#footnote-7)^ | 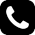 | Home visit/Clinic^[[7]](#footnote-8)^ | Home visit/Clinic^4^ | 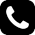 |
| Eligibility assessment and verbal consent | X | X |  |  |  |  |
| Informed consent |  | X^^[[8]](#footnote-9)^^ |  |  |  |  |
| Randomisation |  | X |  |  |  |  |
| Demographics^[[9]](#footnote-10)^ | X | X^[[10]](#footnote-11)^ |  |  |  |  |
| Medical history | X | X |  |  |  |  |
| Concomitant medications | X | X |  | X | X | X |
| HIV status | X | X |  |  |  |  |
| Urine Pregnancy test^^[[11]](#footnote-12)^^ |  | X |  |  | X |  |
| Clinical assessment^[[12]](#footnote-13)^ |  | X |  | X | X |  |
| AEs and SAEs check |  | X |  | X | X | X |
| Issue trial medication |  | X |  |  |  |  |
| Symptoms diary issued to participant |  | X |  |  |  |  |
| Day 5 telephone contact^[[13]](#footnote-14)^ |  |  | X |  |  |  |
| INTERVENTIONS | | | | | | |
| Trial medication intake |  | X | X | X |  |  |
| Witnessed dose |  | X |  | X |  |  |
| ASSESSMENTS | | | | | | |
| Nasopharyngeal swab^[[14]](#footnote-15)^ |  | X |  |  |  |  |
| Saliva sample^[[15]](#footnote-16)^ |  | X | X | X |  |  |
| Temperature recording^[[16]](#footnote-17)^ |  | X | X | X |  |  |
| Blood sample *(for full blood count, Urea & electrolytes, liver function tests, uric acid)* |  | X^[[17]](#footnote-18)^ |  | X | X^[[18]](#footnote-19)^ |  |
| Serum sample for storage |  | X |  | X |  |  |
| Pharmacokinetic plasma samples (2x2mL) |  |  |  | X |  |  |
| Stool samples |  |  |  | X |  |  |
| Trial medication adherence check |  |  |  | X |  |  |
| Tablets count |  |  | X | X |  |  |
| Symptom diary completion by participant |  | X | X | X |  |  |
| Follow up questionnaire |  |  |  |  |  | X |
| Completion including data transfer and query resolution | X | X | X | X | X | X |

### 6.7.1 Early Stopping of Follow-up

If a participant chooses to discontinue their trial treatment, they should continue to be followed up as closely as possible to the follow-up schedule defined in the protocol, providing they are willing to. They should be encouraged and facilitated not to leave the whole trial, even though they no longer take the trial treatment. If, however, the participant exercises the view that they no longer wish to be followed up either, this view must be respected, and the participant withdrawn entirely from the trial. CCTU should be informed of the withdrawal in writing using the appropriate FLARE trial documentation. Data already collected will be kept and included in analyses according to the intention-to-treat principle for all participants who stop follow up early.

Participants who stop trial follow-up early will not be replaced.

If a participant’s baseline diagnostic swab for SARS-CoV-2 is negative, they will be retained in the trial due to the relatively high rate of false negative results in this test. However, if the baseline saliva PCR is also negative, they will be excluded from the modified ITT analysis (see protocol section 6.11.5 Analysis Population and Missing Data).

Participants who start Day 1 of dosing but are deemed ineligible following results from safety bloods collected at Screening/Baseline visit will be considered as screen failure.

### 6.7.2 Participant Transfers

If a participant moves from the area making continued follow up at their consenting centre inappropriate, every effort should be made for them to be visited or to return for at least one visit. Given the short duration of follow-up, this is not anticipated to be a significant issue.

### 6.7.3 Loss to Follow-up

All efforts will be made to ensure complete follow-up all of participants, including via a telephone call (or a clinic visit as per local requirement at some of the international sites) from the trial team on Day 5 to ensure collection of saliva samples for the primary end-point. Given the short duration of follow-up, loss to follow-up is not anticipated to be a significant issue. However, participants failing to attend their scheduled follow-up visits should be contacted as soon as possible to re-arrange their visits at a more suitable date. Participants will be considered as lost to follow-up after three unsuccessful attempts (3 consecutive days) to contact them by the Research staff at participating sites.

### 6.7.4 Trial Closure

When the whole target sample is recruited, the last participant has completed the last follow-up and the database is locked for analysis.

## 6.8 Sample Size

We have conducted extensive power calculation and power simulation exercises for the proposed factorial design. In the proposed design 240 participants (216 evaluable participants, which implies a 10% allowance for attrition and other methodological challenges) will be recruited, so that half of participants receive each of the experimental treatments, one quarter receive both treatments, and one quarter receive neither (with double dummy placebos in place). This sample size has 90% power to detect the effect of each active treatment on its own, with alpha of 2.5%, assuming a difference of 0.9 log_10_ viral load compared to placebo. The interaction term is powered (with 80% power and nominal alpha of 5%) to find a difference, either added to or subtracted from the additive effect of each treatment, of 1.0 log_10_ viral load to be statistically significant.

## 6.9 Recruitment and Retention

### 6.9.1 Recruitment

The trial will be advertised at each site and participant identification centre, as well as public domain websites (such as Sponsor webpage, NIHR Be Part of Research) and sponsor specific social media platforms, and potential participants provided with the trial team’s contact details. Occupational Health teams at each participating centre will be provided with the trial information which they can provide to potential participants who consult them. Potential participants will also be approached after research on databases (e.g., NHS Digital) or via the COVID-19 national NHS Test and Trace Programme. 240 participants will be recruited in total (60 participants in each arm) over an initial period of 14 months.

Should recruitment targets be slower than anticipated other London or UK based NHS organisations and international organisations will be approached to enrol participants, which will be done centrally by the trial team in any case. Recruitment will be monitored regularly to identify any barriers to recruitment. Reporting will be conducted on a regular basis over the trial set up and recruitment period to the Trial Management Group (TMG), trial oversight committees and the funder. Remedial action will be put in place as soon as any concerns have been raised.

**For international sites, country-specific recruitment procedures will be implemented in accordance with local IRB/IEC and national regulatory authority requirements.**

**See country-specific addendum for further details on site-specific procedures and documents.**

### 6.9.2 Retention

Participants will be in regular contact by phone and text message during the trial duration.

## 6.10 Assignment of Intervention

### 6.10.1 Allocation

#### 6.10.1.1 Sequence generation

Participants will be randomised 1:1:1:1 using a concealed online minimisation process, with the following factors: study site, age (≤ 55 vs > 55 years old), gender, obesity (BMI <30 vs ≥30), symptomatic or asymptomatic, current smoking status (Yes = current smoker, No = ex-smoker, never smoker), ethnicity (Caucasian, other), previous COVID-19 specific vaccination (Yes = ≥ 1 dose, No = no previous covid-19 specific vaccination) and presence or absence of comorbidity (defined as diabetes, hypertension, ischaemic heart disease (including previous myocardial infarction), other heart disease (arrhythmia and valvular heart disease), asthma, COPD, other chronic respiratory disease). The minimisation algorithm will incorporate a random element to maximise balance in the stratifying variables between the randomised groups. These data will be collected at the pre-screening telephone contact and will be used for the purposes of registration and IMP kit pre-allocation. Considering the importance of starting therapy immediately upon entering this trial, IMP kit codes will be pre-allocated to the participants ahead of the Screening/Baseline visit on Day 1.

The Trial Statistician at CCTU will generate a sequence of unique identifiers for every active/placebo IMP kit. A copy of the sequence will be held securely at CCTU.

#### 6.10.1.2 Randomisation concealment mechanism

The IMP kit codes will be provided to Sealed Envelope and the IMP final manufacturer (RenaClinical Limited) such that the trial team and participants remain blind to treatment allocation.

#### 6.10.1.3 Randomisation Implementation

Minimisation is a dynamic system that depends on previous participants enrolled in the trial.

Following the pre-screening telephone contact before Screening/Baseline visit, the clinical investigator or delegate will enter the participant’s initials, gender, age, height, weight and other factors detailed in Section 6.10.1.1 and confirm participant eligibility on the SealedEnvelope.com secure online system and allocate the appropriate PIN to the patient.

Delegated staff at participating sites will be provided with a secure login to the SealedEnvelope.com website, according to their role in the trial. The randomisation result will be shown directly online as a unique IMP kit code, with an email confirmation sent to the user and to the CCTU trial team. The investigator will provide details of the allocated unique kit code assigned to the participant on the prescription. Delegated staff will collect the allocated IMP kit from pharmacy on behalf of the participant ahead of the Screening/Baseline visit.

Randomisation will be considered complete after the participant has signed the consent form, ingested their first dose of trial medication during Screening/Baseline visit and is deemed eligible following results from safety bloods collected at Screening/Baseline visit. Participants who withdraw consent before taking their first dose or those who start Day 1 of dosing but are deemed ineligible following results from safety bloods will be considered as screen failures.

### 6.10.2 Blinding

Participants and investigators will both be blinded to treatment allocation (double blinded). All IMP will be packaged and labelled to maintain blinding, and blinded IMP kit codes will be used to identify the medication. IMP kit codes will be linked to the PIN allocated to the participant at randomisation.

Detailed information regarding allocation implementation and blinding is provided in the IMP Management Plan and FLARE Randomisation and Unblinding Plan.

### 6.10.3 Emergency Unblinding

If the participant reports overdosing on study medication and is hospitalised as a result, emergency unblinding will take place.

All recruited participants will be given a card with contact details for the trial team including emergency contact 24 hours a day, 7 days per week. In the event of unblinding becoming necessary, emergency unblinding can occur at any time through the 24-hour web-based service offered by SealedEnvelope.com. Unblinding may occur for any participant experiencing a medical emergency for which the clinical management will be facilitated by the unblinding of the patient’s treatment allocation. The Principal Investigator (PI) cannot overrule any decision made by a referring clinician. It is anticipated that in most instances, appropriate clinical management can proceed with the assumption that the patient has been treated with active IMP without needing to unblind the participant.

Detailed information regarding unblinding is provided in the Randomisation and Unblinding Plan.

### 6.10.4 Unblinding for the submission of SUSAR reports

All SAEs that are related to the trial medication (i.e., SARs) and are suspected to be unexpected i.e., SUSARs, need to be submitted to the regulatory agencies within pre-specified timelines. When SAEs reports are received at the CCTU, if the event is recorded as being a SUSAR then the following procedure will be used to unblind the SUSAR to determine if the participant was receiving active trial medication, and therefore, that the SUSAR needs onward reporting to the regulatory agencies:

- A member of the CCTU trial SUSAR Reporting Team will unblind the participant’s trial treatment allocation using the Sealed Envelope randomisation service.
- If the participant is revealed to the CCTU SUSAR Reporting team to be receiving active treatment, the CCTU trial SUSAR Reporting Team member will report the SUSAR on the e-SUSAR database available through the MHRA website and to the MHRA and REC as required.
- This information will not be forwarded to the trial team at the CCTU or at the sites. It will be kept in a separate file by the CCTU SUSAR reporting team.

## 6.11 Data Collection, Management and Analysis

### 6.11.1 Data Collection Methods

Each participant will be given a unique trial Participant Identification Number (PIN). Data will be collected at the time-points indicated in the Trial Schedule.

Data will be entered onto paper Case Record Forms (CRFs) prior to entry onto the database. Staff will receive training on data collection. Paper CRFs will be placed in clear plastic wallets which allow reading both sides of the CRF without opening the wallets. The plastic wallets will be decontaminated before leaving the participant's residence as per infection control guidance.

Data collection, data entry and queries raised by a member of the FLARE trial team will be conducted in line with the CCTU Standard Operating Procedures and the FLARE Data Management Plan.

Identification logs, screening and enrolment logs will be kept at the trial site in a locked cabinet within a secured room.

Research team members across all participating sites will receive training on the FLARE protocol and on the data entry in the approved FLARE database.

All data will be handled in accordance with the Data Protection Act 2018 and the GDPR 2016/679 including all further updates.

### 6.11.2 Data Management

Data will be entered remotely in the approved FLARE database by delegated members of the research team at sites and protected using established CCTU procedures.

Coded data: Participants will be given a unique trial Participant Identification Number (PIN). Data will be entered under this identification number onto the central database stored on the servers based at CCTU. The database will be password protected and only accessible to members of the FLARE trial team at CCTU, and external regulators if requested. The servers are protected by firewalls and are patched and maintained according to best practice. The physical location of the servers is protected by CCTV and security door access.

The database and coding frames have been developed by the Clinical Trial Manager in conjunction with CCTU. The database software provides a number of features to help maintain data quality, including maintaining an audit trail, allowing custom validations on all data, allowing users to raise data query requests, and search facilities to identify validation failure/ missing data.

After completion of the trial, the database will be retained on the servers of UCL for on-going analysis of secondary outcomes.

The identification, screening and enrolment logs, linking participant identifiable data to the pseudoanonymised PIN, will be held locally by the trial site. This will either be held in written form in a locked filing cabinet or electronically in password protected form on hospital computers. After completion of the trial the identification, screening and enrolment logs will be stored securely by the sites for 10 years unless otherwise advised by CCTU.

### 6.11.3 Non-Adherence and Non-Retention

Every attempt to contact participants who do not adhere to the trial or who terminate participation prematurely will be made. This will include a telephone call on Day 5 from delegated members of the research team at sites. Participants will complete diaries and tablet counts at the end of the intervention period will be carried out. Reasons for non-adherence or non-retention will be documented.

If a participant wishes to withdraw consent (full withdrawal from treatment and follow-up), this should be documented on the withdrawal CRF. This conversation should be documented, and the correct level of withdrawal selected on the CRF. Once consent has been withdrawn all follow-up will cease but all the information already obtained will be kept. To safeguard participant rights, only the minimum personally identifiable information possible will be used.

Due to the nature of the trial database and processes in place for auditing and accountability for trial data collection, it is not possible to remove all the data collected on the participant from the trial. Participants will be made aware of this at the time of recruitment.

### 6.11.4 Statistical Methods

#### 6.11.4.1 Statistical Analysis Plan

A statistical analysis plan will be written and approved by the Trial Steering Committee and Independent Data Monitoring Committee prior to the first formal data analysis. The rest of this section provides the general statistical principles.

#### 6.11.4.2 Statistical Methods – Outcomes

1. A CONSORT diagram will be produced to report of the flow of participants in the trial.

Summary of baseline characteristics, by trial arms, will be by frequency and percentage for categorical variables, and for continuous variables by mean and standard deviation (or median and inter-quartile range for non-normally distributed data).

The primary endpoint will be analysed using an analysis of covariance (ANCOVA) model, including each experimental treatment as factors, the interaction between them, the baseline (Day 1) values of viral load and site of recruitment.

Secondary endpoints include binary endpoints and continuous endpoints (note that the pharmacokinetic analysis of favipiravir is described in a separate section below).

For binary endpoints, a logistic regression will be used.

The comparison of duration of fever following commencement of medication between trial arms will be by t-test, or non-parametric equivalent depending on the distribution of the data.

#### 6.11.4.3 Additional Analyses – Subgroup

Using an interaction term, we will explore whether a differential treatment effect is observed according to the following factors: sex (male vs female), age (≤ 55 vs > 55), obesity (BMI <30 vs ≥30), symptomatic or asymptomatic, current smoking status (Yes = current smoker, No = ex-smoker, never smoker), ethnicity (Caucasian, other), previous COVID-19 specific vaccination (Yes = ≥ 1 dose, No = no previous covid-19 specific vaccination) and presence or absence of comorbidity (defined as diabetes, hypertension, ischaemic heart disease (including previous myocardial infarction), other heart disease (arrhythmia and valvular heart disease), asthma, COPD, other chronic respiratory disease).

#### 6.11.4.4 Additional Analyses – Adjusted

The primary outcome model will be adjusted for baseline viral load and site of recruitment. The secondary outcome model of rate of decrease of viral load will also be adjusted for baseline viral load and site of recruitment.

### 6.11.5 Analysis Population and Missing Data

The primary analysis will be in the intention-to-treat (ITT) population. There will be no imputation for missing data for any of the study outcomes.

A modified ITT analysis will be performed that will only include participants who have a confirmed diagnosis at baseline of SARS-CoV-2 infection (diagnostic swab and saliva samples collected at Baseline).

#### 6.11.6 Pharmacometric evaluations

On Day 7, a pre-dose and post-dose plasma samples will be taken for all participants in each arm. Participants will be asked to record the time to the nearest minute of each dose taken on Day 7.

Further details of sample processing are in section 6.5 Sample Management.

#### 6.11.6.1 Pharmacometric Analysis Plan

Population PKPD modelling and dosing simulations will be undertaken with non-linear mixed-effects modelling. Viral load with time will be modelled with a viral dynamic model, and the influence of favipiravir included to estimate an in vivo EC50.

Plasma concentrations will be sent from the bioanalytical laboratory on a spreadsheet, and dosing history and relevant covariate information extracted from the clinical database. These data will be imported into R (version 3.4 or above) and merged for exploratory analysis and formatted for subsequent modelling using NONMEM version 7.4 or above (Globomax, USA) and or nlmixr version 1.0 or above.

Model selection criteria will include: (i) successful minimisation, (ii) standard error of estimates, (iii) number of significant digits, (iv) termination of the covariance step and (v) correlation between model parameters. Goodness of fit will be assessed by graphical methods, including population and individual predicted vs. observed concentrations, conditional weighted residual vs. observed concentrations and time, correlation matrix for fixed vs. random effects, correlation matrix between parameters and covariates and normalised predictive distribution error (NPDE). Comparison of hierarchical models will be based on the likelihood ratio test. A superior model will be also expected to reduce inter-subject variance terms and/or residual error terms. Standard error of the parameter estimates will be approximated using of the asymptotic covariance matrix.

#### 6.11.6.2 Additional analyses

Viral sequencing will be undertaken according to established protocols and analysis of mutagenesis or the appearance of potentially deleterious mutations will be interrogated as previously described for other RNA viruses^10^.

## 6.12 Data Monitoring

### 6.12.1 Data Monitoring Committee

An Independent Data Monitoring Committee (IDMC) will be appointed for the trial, the IDMC will review unblinded data and make recommendations to the Trial Steering Committee. Further details of the roles and responsibilities of the IDMC, including membership, relationships with other committees, decision making processes, and the timing and frequency of interim analyses, (and description of stopping rules and/or guidelines where applicable) are described in detail in the FLARE IDMC Terms of Reference (ToR).

### 6.12.2 Interim Analyses

There will be no interim analyses. Monitoring of the safety of the trial will be undertaken by the IDMC which will have untrammeled access to the trial data, and whose work is governed by a separate charter.

### 6.12.3 Data Monitoring for Harm

Descriptive statistics will be used to compare rates of adverse events between treatment arms. No additional formal monitoring will be performed except from those required by the IDMC.

#### 6.12.3.1 Safety reporting

Definitions of harm of the EU Directive 2001/20/EC Article 2 based on the principles of ICH GCP apply to this trial.

Table 2: Adverse Event Definitions

| **Adverse Event (AE)** | Any untoward medical occurrence in a patient or clinical trial participant administered a medicinal product and which does not necessarily have a causal relationship with this product. |
| --- | --- |
| **Adverse Reaction (AR)** | Any untoward and unintended response to an investigational medicinal product related to any dose administered |
| **Unexpected Adverse Reaction (UAR)** | An adverse reaction, the nature or severity of which is not consistent with the applicable product information (e.g. Investigator’s Brochure for an unauthorised product or summary of product characteristics (SPC) for an authorised product) |
| **Serious Adverse Event (SAE) or Serious Adverse Reaction (SAR)** | Any AE or AR that at any dose:   - results in death - is life threatening* - requires hospitalisation or prolongs existing hospitalisation** - results in persistent or significant disability or incapacity - is a congenital anomaly or birth defect - or is another important medical condition*** |
| **Suspected Unexpected Serious Adverse Reaction (SUSAR)** | A serious adverse reaction, the nature of severity of which is not consistent with the known potentially expected events associated with the applicable trial treatment. The event is evaluated as having a possible, probable or definite relationship to a trial treatment and is unexpected for that trial treatment.  OR  An unexpected adverse reaction is one that is not reported in the current and approved IB and SPCs section 4.8 or one that is more frequently reported or more severe than previously reported. If a SAR is assessed as being unexpected it becomes a SUSAR (Suspected, Unexpected, Serious Adverse Reaction). |

| * the term life threatening here refers to an event in which the patient (or participant) is at risk of death at the time of the event; it does not refer to an event that might hypothetically cause death if it was more severe (e.g., a silent myocardial infarction)  ** Hospitalisation is defined as an in-patient admission, regardless of length of stay, even if the hospitalisation is a precautionary measure for continued observation. Hospitalisation for pre-existing conditions (including elective procedures that have not worsened) do not constitute an SAE  *** Medical judgement should be exercised in deciding whether an AE or AR is serious in other situations. Important AEs or Ars that may not be immediately life threatening or result in death or hospitalisation, but may seriously jeopardise the participant by requiring intervention to prevent one of the other outcomes listed in the table (e.g., a secondary malignancy, an allergic bronchospasm requiring intensive emergency treatment, seizures or blood dyscrasias that do not require hospitalisation, or development of drug dependency). |
| --- |

##### *6.12.3.1.1 Adverse Reactions*

**Phototoxicity:**

Due to the phototoxicity potential of favipiravir, participants should avoid excessive exposure to sunlight or artificial ultraviolet light.

Adverse reactions potentially related to favipiravir and lopinavir/ritonavir are listed in **Appendix 4**.

##### *6.12.3.1.2 Adverse Events*

Adverse events (AEs) include:

- an exacerbation (i.e., increase in the frequency or intensity) of a pre-existing illness, episodic event or symptom (initially recorded at the screening/baseline visit), that is detected after trial drug administration/intervention
- Occurrence of a new illness, episodic event or symptom, that is detected after trial drug administration/intervention
- Any overdose of trial medication must be reported
- Abnormalities shown on blood sample results which are considered in the opinion of the investigator as clinically significant against the local blood sample reference ranges.

Adverse events do NOT include:

- Medical or surgical procedures: the condition that leads to the procedure is the AE
- Pre-existing disease or a condition present before treatment that does not worsen
- Hospitalisation where no untoward or unintended response has occurred e.g. elective cosmetic surgery

#### 6.12.3.4 Procedures to follow in the event of female participants or female partners of male participants becoming pregnant

Highly effective contraception, supplemented with a barrier method (preferably male condom), is required for this trial as favipiravir has potential teratogenic properties. Female participants with a positive pregnancy test at screening will not be eligible for inclusion in this trial. Participants will be advised to contact the trial team immediately if they, or the female partners of male participants, become pregnant during the treatment period or 7 days afterwards. Pregnant participants or partners will be advised to discontinue the trial medication immediately and to seek urgent medical advice.

All pregnancies and suspected pregnancies occurring in participants must be reported immediately to UCL CCTU upon site becoming aware using the Pregnancy Notification & Outcome Form CRF. A follow up Pregnancy report must be completed which will provide further information on pregnancy outcomes when this becomes available. Informed consent from the patient must be sought prior to collecting information on pregnancy outcomes. Any congenital abnormality or birth defect resulting from the pregnancy should be reported as an SAE to UCL CCTU.

#### 6.12.3.5 Investigator responsibilities relating to safety reporting

All non-serious AEs and Ars, whether expected or not, should be recorded in the participant Adverse Event Log and reported to CCTU within 14 days. SAEs and SARs should be notified to CCTU immediately the investigator becomes aware of the event (in no circumstance should this notification take longer than 24 hours).

##### 6.12.3.5.1 Seriousness assessment

When an AE or AR occurs, the investigator responsible for the care of the participant must first assess whether or not the event is serious using the definition given in Table 2. If the event is classified as ‘serious’ then an SAE form must be completed and CCTU (or delegated body) notified immediately (no longer that 24hours).

##### 6.12.3.5.2 Severity or grading of Adverse Events

The severity of all AEs and/or Ars (serious and non-serious) in this trial should be graded using the toxicity gradings in Common Terminology Criteria for Adverse Events (CTCAE) v5.0.

**Grade 1:** Mild; asymptomatic or mild symptoms; clinical diagnostic observations only; intervention not indicated.

**Grade 2:** Moderate; minimal, local or non-invasive intervention indicated; limiting age-appropriate instrumental activities of daily living (ADL)*.

**Grade 3:** Severe or medically significant but not life threatening; hospitalisation or prolongation of hospitalisation indicated; disabling; limiting self-care ADL**.

**Grade 4:** Life threatening consequences; urgent intervention indicated.

**Grade 5**: Death related to AE or AR.

*Instrumental ADL refer to preparing meals, shopping for groceries or clothes, using the telephone, managing money etc.

** Self-care AD refer to bathing, dressing and undressing, feeding self, using the toilet, taking medications and not bedridden.

##### 6.12.3.5.3 Causality

The investigator must assess the causality of all serious events or reactions in relation to the trial therapy using the definitions in Table 3.

Table 3: Causality definitions

| Relationship | Description | Event type |
| --- | --- | --- |
| Unrelated | There is no evidence of any causal relationship | Unrelated SAE |
| Unlikely to be related | There is little evidence to suggest that there is a causal relationship (e.g., the event did not occur within a reasonable time after administration of the trial medication). There is another reasonable explanation for the event (e.g., the participant’s clinical condition or other concomitant treatment) | Unrelated SAE |
| Possibly related | There is some evidence to suggest a causal relationship (e.g., because the event occurs within a reasonable time after administration of the trial medication). However, the influence of other factors may have contributed to the event (e.g., the participant’s clinical condition or other concomitant treatment) | SAR |
| Probably related | There is evidence to suggest a causal relationship and the influence of other factors is unlikely | SAR |
| Definitely related | There is clear evidence to suggest a causal relationship and other possible contributing factors can be ruled out. | SAR |

If an SAE is considered to be related to trial treatment, and treatment is discontinued, interrupted or the dose modified, refer to the relevant Interventions sections of the protocol.

##### 6.12.3.5.4 Expectedness

If there is at least a possible involvement of the trial medications (including any comparators), the sponsor will assess the expectedness of the event. An unexpected adverse reaction is one that is not reported in the current Favipiravir IB and Lopinavir/ritonavir (LPV/r) SPC, or one that is more frequently reported or more severe than previously reported. The reference safety information (RSI) for this trial are the current version of the IB for Favipiravir (specifically section 7.13 ‘Reference Safety Information’) and the SPC for Lopinavir/ritonavir (LPV/r) (specifically section 4.8 ‘Undesirable effects’). See the appended package inserts for a list of expected toxicities associated with the drugs being used in this trial. If a SAR is assessed as being unexpected it becomes a SUSAR (suspected, unexpected, serious adverse reaction). MHRA reporting guidelines apply (see protocol section 6.12.3.6 Notifications).

#### 6.12.3.6 Notifications

##### 6.12.3.6.1 Notifications by the Investigator to CCTU

CCTU must be notified of all SAEs within 24 hours of the investigator becoming aware of the event.

Investigators should notify CCTU of any SAEs and other Notifiable Adverse Events (NAEs) occurring from the time of randomisation until 30 days after the last protocol treatment administration, including SARs and SUSARs. From this point forward the site will not actively monitor SAEs or NAEs but will notify the CCTU of any SARs and SUSARs if they become aware of them until trial closure.

Any subsequent events that may be attributed to treatment should be reported to the MHRA using the yellow card system (https://yellowcard.mhra.gov.uk/the-yellow-card-scheme/).

The SAE form must be completed by the investigator (the consultant named on the delegation of responsibilities list who is responsible for the participant’s care) with attention paid to the grading, and causality of the event. In the absence of the responsible investigator, the SAE form should be completed and signed by a member of the site trial team and emailed as appropriate within the timeline. The responsible investigator should check the SAE form at the earliest opportunity, make any changes necessary, sign and then email to CCTU. Detailed written reports should be completed as appropriate. Systems will be in place at the site to enable the investigator to check the form for clinical accuracy as soon as possible.

The minimum criteria required for reporting an SAE are the trial number and date of birth, name of reporting investigator and sufficient information on the event to confirm seriousness. Any further information regarding the event that is unavailable at the time of the first report should be sent as soon as it becomes available.

The SAE form must be scanned and sent by email to the trial team at CCTU on [cctu.flare@ucl.ac.uk](mailto:cctu.flare@ucl.ac.uk) with the subject ‘SAE FLARE’

Participants must be followed up until clinical recovery is complete and laboratory results have returned to normal or baseline values, or until the event has stabilised. Follow-up should continue after completion of protocol treatment and/or trial follow-up if necessary. Follow-up SAE forms (clearly marked as follow-up) should be completed and emailed to CCTU as further information becomes available. Additional information and/or copies of test results etc may be provided separately. The participant must be identified by trial number, date of birth and initials only. The participant’s name should not be used on any correspondence and should be blacked out and replaced with trial identifiers on any test results.

##### 6.12.3.6.2 CCTU responsibilities

Medically qualified staff at CCTU and/or the Chief Investigator (CI or a medically qualified delegate) will review all SAE reports received. The sponsor clinical reviewer will complete the assessment of expectedness in light of the Reference Safety Information (RSI).

CCTU is undertaking the duties of trial sponsor and is responsible for the reporting of SUSARs and other SARs to the regulatory authorities (MHRA) and the Ecs as appropriate. Fatal and life threatening SUSARs must be reported to the competent authorities within 7 days of CCTU becoming aware of the event; other SUSARs must be reported within 15 days.

CCTU will keep investigators informed of any safety issues that arise during the course of the trial.

The trial manager or delegate at CCTU will submit Development Safety Update Reports (DSURs) to competent authorities.

### 6.12.4 Quality Assurance and Control

#### 6.12.4.1 Definitions

Quality Assurance (QA), defined as all the planned and systematic actions established to ensure the study is performed and data generated, documented and/or recorded and reported in compliance with the principles of ICH GCP and applicable regulatory requirements.

Quality Control (QC), defined as the operational techniques and activities performed within the Quality Management System (QMS) to verify that the requirements for quality of the trial related activities are fulfilled.

#### 6.12.4.2 Quality and Risk Management

Quality Assurance (QA) for the FLARE trial will implemented as described in CCTU Quality Management Policy and all SOPs and working instructions, which define the CCTU QMS that are applicable to the study’s completion. The study proposal from the Chief Investigator is reviewed and developed in line with the processes required by the Trial Adoption Group (TAG) and this protocol has been reviewed, updated and finalised passing through the Protocol Review Committee process. The study will be subject to a dynamic risk assessment process throughout its life cycle; the CCTU Quality Management Group reviews the risk assessment periodically. Study risks are defined in terms of their impact on: the rights and safety of participants, project concept including study design, reliability of results and institutional risk; project management; and other considerations.

All aspects of the FLARE trial will be subject to the procedures followed by the CCTU QA function, this includes delivery of the CCTU Audit Programme and other due diligence procedures (vendor assessments). Aspects and entities that are subject to QA function procedures include, but are not limited to; outcome analysis laboratories, Contract Research Organisations CROs, study sites and internal CCTU procedures which apply to the FLARE trial.

#### 6.12.4.3 Central Monitoring at CCTU

CCTU staff will review Case Report Form (CRF) data for errors and missing key data points. The trial database will also be programmed to generate reports on errors and error rates. Essential trial issues, events and outputs, including defined key data points, will be detailed in the FLARE trial Data Management Plan and Quality Management and Monitoring Plan (QMMP).

#### 6.12.4.3 On-site Monitoring

The frequency, type and intensity of routine and triggered on-site monitoring will be detailed in the FLARE Quality Management and Monitoring Plan (QMMP). The QMMP will also detail the procedures for review and sign-off of monitoring reports. In the event of a request for a trial site inspection by any regulatory authority UCL CCTU must be notified as soon as possible.

##### 6.12.4.3.1 Direct access to participant records

Participating investigators must agree to allow trial related monitoring, including audits, REC review and regulatory inspections, by providing access to source data and other trial related documentation as required. Participant consent for this must be obtained as part of the informed consent process for the trial.

#### 6.12.4.4 Trial Oversight

Trial oversight is intended to preserve the integrity of the trial by independently verifying a variety of processes and prompting corrective action where necessary. The processes reviewed relate to participant enrolment, consent, eligibility, and allocation to trial groups; adherence to trial interventions and policies to protect participants, including reporting of harms; completeness, accuracy and timeliness of data collection; and will verify adherence to applicable policies detailed in the Compliance section of the protocol. Independent trial oversight complies with the CCTU trial oversight policy.

In multi-centre trials this oversight is considered and described both overall and for each recruiting centre by exploring the trial dataset or performing site visits as described in the FLARE QMMP.

##### 6.12.4.4.1 Trial Team

The Trial Team (TT) will be set up to assist with developing the design, co-ordination and day to day operational issues in the management of the trial, including budget management. The membership, frequency of meetings, activity (including trial conduct and data review) and authority will be covered in the TT terms of reference.

##### 6.12.4.4.2 Trial Management Group

A Trial Management Group (TMG) will be set up to assist with developing the design, co-ordination and strategic management of the trial. The membership, frequency of meetings, activity (including trial conduct and data review) and authority will be covered in the TMG terms of reference.

##### 6.12.4.4.3 Independent Trial Steering Committee

The Independent Trial Steering Committee (TSC) is the independent group responsible for oversight of the trial in order to safeguard the interests of trial participants. The TSC provides advice to the CI, CCTU, the funder and sponsor on all aspects of the trial through its independent Chair. The membership, frequency of meetings, activity (including trial conduct and data review) and authority will be covered in the TSC terms of reference.

##### 6.12.4.4.4 Independent Data Monitoring Committee

The Independent Data Monitoring Committee (IDMC) is the only oversight body that has access to unblinded accumulating comparative data. The IDMC is responsible for safeguarding the interests of trial participants, monitoring the accumulating data and making recommendations to the TSC on whether the trial should continue as planned. The membership, frequency of meetings, activity (including review of trial conduct and data) and authority will be covered in the IDMC terms of reference. The IDMC will advise the TSC through its Chair.

##### 6.12.4.4.5 Trial Sponsor

The role of the sponsor is to take on responsibility for securing the arrangements to initiate, manage and finance the trial. UCL is the trial sponsor and has delegated the duties as sponsor to CCTU via a signed letter of delegation.

# 7 Ethics and Dissemination

## 7.1 Ethics Committee Approval

Before initiation of the trial at any clinical site, the protocol, all informed consent forms and any material to be given to the prospective participant will be submitted to the relevant EC for approval. Any subsequent amendments to these documents will be submitted for further approval. Before initiation of the trial at each additional clinical site, the same/amended documents will be submitted for local permissions.

The rights of the participant to refuse to participate in the trial without giving a reason must be respected. After the participant has entered the trial, the clinician remains free to give alternative treatment to that specified in the protocol, at any stage, if they assess this to be in the best interest of the participant. The reasons for doing so must be recorded. After randomisation the participant must remain within the trial for the purpose of follow up and data analysis according to the treatment option to which they have been allocated. However, the participant remains free to change their mind at any time about the protocol treatment and follow-up without giving a reason and without prejudicing their further treatment.

**For international sites, this protocol will be submitted to the country-specific IRB/IEC and approvals will be required to be in place before the commencement of the trial.**

**See country-specific addendum for further details on national procedures and documents.**

## 7.2 Competent Authority Approvals

This protocol will be submitted to the MHRA.

This is a Clinical Trial of an Investigational Medicinal Product (IMP) as defined by the EU Directive 2001/20/EC. Therefore, a CTA is required in the UK.

**For international sites, this protocol will be submitted to the** **country-specific national regulatory authority and approval will be required to be in place before the commencement of the trial.**

**See country-specific addendum for further details on national procedures and documents.**

The progress of the trial, safety issues and reports, including expedited reporting of SUSARs, will be reported to the Competent Authority, regulatory agency or equivalent in accordance with relevant national and local requirements and practices.

## 7.3 Other Approvals

The protocol will be submitted by those delegated to do so to the Health Research Authority for national approval and relevant R&D departments of each participating site for review of local Trust Capacity and Capability. A copy of the local permissions (or other relevant approval as above) and of the Participant Information Sheet (PIS) and consent form on local headed paper must be forwarded to the co-ordinating centre before participants are randomised to the trial.

The protocol has received formal review and methodological, statistical, clinical and operational input from the CCTU Protocol Review Committee.

## 7.4 Protocol Amendments

Protocol amendments will be agreed between the co-investigators and Trial Steering Committee. They will be submitted by the CI to the REC and MHRA and disseminated to the study team.

**For international sites, protocol amendments will be submitted in accordance with local IRB/IEC and national regulatory authority requirements and approvals will be required to be in place before implementation at sites.**

**See country-specific addendum for further details on national procedures and documents.**

## 7.5 Consent or Assent

Participants will be provided with a Participant Information Sheet (PIS) and given time to read it fully. Following a discussion with a medical qualified investigator or suitable trained and authorised delegate, any questions will be satisfactorily answered and if the participant is willing to participate, written informed consent will be obtained. During the consent process it will be made completely and unambiguously clear that the participant is free to refuse to participate in all or any aspect of the trial, at any time and for any reason, without incurring any penalty or affecting their treatment.

Consent will be re-sought if new information becomes available that affects the participant’s consent in any way. This will be documented in a revision to the participant information sheet and the participant will be asked to sign an updated consent form. These will be approved by the ethics committee prior to their use.

A copy of the approved consent form is available from the CCTU trial team.

**For international sites, approved country-specific** **Participant Information Sheet (PIS) and Informed Consent Form (ICF) will be used to document consent procedure.**

**See country-specific addendum for further details on national consent procedures and documents.**

## 7.6 Confidentiality

Adequate measures will be in place to ensure all participant data collected are secure. Each participant will be assigned a unique trial Participant Identification Number (PIN). CRFs will record the participant initials and month/year of birth but not their name. The only link between the PIN and the participant’s name will be on the screening log kept at site and accessed only by the site trial team.

Data will be recorded on the CRFs and entered onto the FLARE custom-designed database under this identification number. The database will be password protected and only accessible to members of the FLARE trial team at CCTU, trained and authorised site staff, and external regulators if requested. The servers are protected by firewalls and are patched and maintained according to best practice. The physical location of the servers is protected by CCTV and security door access.

Blood samples will be labelled with the FLARE participant identification number ensuring the pseudonymity of the participants who have provided the samples. At the laboratories (see section 6.5 Sample Management), brief clinical details will be stored including age and sex. Blood results will be stored on a web-based, secure confidential database, including after completion of the FLARE trial.

## 7.7 Declaration of Interests

The investigators named on the protocol have no financial or other competing interests that impact on their responsibilities towards the scientific value or potential publishing activities associated with the trial.

## 7.8 Indemnity

UCL holds insurance to cover participants for injury caused by their participation in the FLARE trial. Participants may be able to claim compensation if they can prove that UCL has been negligent. However, as the FLARE trial is being carried out by hospital staff, the hospital continues to have a duty of care to the participant in the FLARE trial. UCL does not accept liability for any breach in the hospital’s duty of care, or any negligence on the part of hospital employees. This applies whether the hospital is an NHS Trust or otherwise. This does not affect the participant’s right to seek compensation via the non-negligence route.

Participants may also be able to claim compensation for injury caused by participation in the FLARE trial without the need to prove negligence on the part of UCL or another party. Participants who sustain injury and wish to make a claim for compensation should do so in writing in the first instance to the Chief Investigator, who will pass the claim to UCL’s insurers, via the Sponsor’s office.

Hospitals selected to participate in the FLARE trial shall provide clinical negligence insurance cover for harm caused by their employees and a copy of the relevant insurance policy or summary shall be provided to UCL, upon request.

**For international sites, a country-specific** **reinsurance policy will be in place. Policies will be issued in accordance with market standard wordings and will meet minimum statutory insurance coverage requirements wherever they exist. A Limit of Indemnity will be provided in each country and will be subject to changes in the country’s taxes.**

**See country-specific addendum for further details on national consent procedures and documents.**

## 7.9 Finance

FLARE is fully funded by LifeArc, grant reference [COVID0005]. It is not expected that any further external funding will be sought.

FUJIFILM Toyama Chemical Co., Ltd. Will provide Favipiravir and matching placebo for the trial free of charge.

## 7.10 Archiving

The investigators agree to archive and/or arrange for secure storage of FLARE trial materials and records for a minimum of 5 years after the close of the trial unless otherwise advised by the CCTU.

## 7.11 Access to Data

The CI, CPM, Trial Manager, Data Manager, Statistician and Trial Management Team will have full access to the trial data. Requests for access to trial data will be considered, and approved in writing where appropriate, after formal application to the TSC. Considerations for approving access are documented in the TSC Terms of Reference.

## 7.12 Ancillary and Post-trial Care

No arrangements will be in place for trial treatment after the planned treatment phase. Participants will be cared for as per standard NHS care (or as per country-specific healthcare system for international sites) should they require further treatment or hospitalisation for their COVID-19 symptoms. This will be made clear in the Participant Information Sheet.

## 7.13 Publication Policy

### 7.13.1 Trial Results

The results of the trial will be published in peer-reviewed journals and may be presented at academic conferences. The results will be communicated to trial participants who have requested to be informed. Results will be reported within 6 months of the end of the trial. The results of the trial will be disseminated regardless of the direction of effect.

Publication and dissemination of the results will be coordinated by the FLARE trial team, following the CCTU Publication Policy.

### 7.13.2 Authorship

All investigators and any other individual who has made a significant intellectual contribution (at the discretion of the Chief Investigator) will be authors. No professional writing assistance will be sought.

### 7.13.3 Reproducible Research

Requests to access the full protocol, dataset and statistical code will be granted to editors and reviewers of submitted manuscripts detailing the results of the trial and will be considered for other researchers or entities on a case-by-case basis by the TSC.

# 8 Ancillary Studies

There are no ancillary studies.

# 9 Protocol Amendments

| **Protocol Version Number** | **Protocol Date** | **Summary of Changes** |
| --- | --- | --- |
| 1.0 | 03-Jul-2020 | N/A |
| 2.0 | 13-Jul-2020 | Changes requested by the MHRA during initial review: addition of wording about the potential phototoxicity of favipiravir and updated Section 6.12.3.5.4 of the protocol (Reference safety). |
| 3.0 | 29-Sep-2020 | - Update to the protocol to broaden the entry criteria from key workers and their household contacts to all adults aged 18-70 years as the population at risk has expanded. This maximises recruitment and opens the trial to more potential participants. We have specified that participants can attend for recruitment visits in a designated area of the hospital site, rather than only as a home visit.  - General administrative changes (e.g., staff and trial management membership)  - Change in the processing of the plasma samples for pharmacokinetic analysis, specifying that sample inactivation should be with ethanol rather than heat, and that storage temperature can be at -20 C or below  - Updated the statistical analysis section (no changes to participant numbers, endpoints or the running of the trial) |
| 4.0 | 07-Jan-2021 | - General administrative changes (e.g., staff and trial management and IDMC membership) - Addition of exclusion criteria for Participants who have received the COVID-19 vaccine. - Addition of flexibility window to the following visits: - Day 5 Telephone contact: as many site research staff do not work during the weekends, participants randomised on a Tuesday or a Wednesday may receive their Day 5 telephone call on Friday of the same week. - Day 7 and Day 14 visits: as site research staff do not work during the weekends, participants randomised on Mondays will not be able to attend their Day 7 visit on Sundays and PK samples will not be collected for these participants. Where possible, Day 7 visits will be re-scheduled for the following Monday (Day 8). We have introduced more flexibility for the Day 14 visit (up to +3 days). - Changes for Day 14 Visit: - to remove the safety blood tests at Day 14 for participants where no abnormal results are observed on Day 7. - to remove the collection of serum sample for storage - to remove the collection of stool sample - Updated secondary outcomes accordingly following the changes for Day 14 Visit - Changes to Day 7 temperature recordings for time points 2, 3 and 4: as sites have been instructed to collect participant’s diary at visit 2 (Day 7), temperature recording for time points 2, 3 and 4 are likely to not be recorded as participants will have their visit before these time points - Section 6.4.3 Addition of guidance for sites where unused medication cannot be destroyed or disposed by pharmacy as part of the site local policy for COVID-19 trials - Section 6.5.1 Update of schedule of samples table following changes to Day 14 visit. - Section 6.7 Updated participant timeline table to reflect all changes regarding Day 5, Day 7 and Day 14 visits - Section 6.7.1 Clarification regarding participants who start Day 1 of dosing but are deemed ineligible following results from safety bloods collected at Screening/Baseline visit - Section 6.7.3 Guidance regarding sites contacting participants lost to follow-up |
| 5.0 | 18-May-2021 | - General administrative changes (e.g., staff and trial management and TMG membership) - Corrections, formatting and minor amendments to the overall text. - Protocol version history updated. - Section 1.1 Compliance: International sites GCP requirements added. - Section 1.3 Structured trial summary, Countries of Recruitment: Updated countries of recruitment to add international sites - Section 1.3 Structured trial summary, Exclusion criteria: Addition of specification for exclusion criterion (7): Hospitalisation for isolation or infection control reasons will not considered an exclusion for this trial. - Section 1.3 Structured trial summary, Study type: Trial design – Updated wording to specify that the trial is a multi-site, UK and international trial. - Section 1.3 Structured trial summary, Key Secondary Outcomes – Addition of specification for secondary outcome 2 to clarify that the outcome will not be applicable to international sites as stool samples will not be collected internationally. - Section 1.4.3 Trial Team: Removal of Trial Team member Amalia Ndoutoumou. - Section 1.4.4 Trial Management Group: Removal of TMG member Amalia Ndoutoumou and addition of Anna Checkley. - Section 3 Abbreviations – Addition of two abbreviations: IEC and IRB - Section 5.3 Trial Design – Updated wording to specify that the trial is a multi-site, UK and international trial. - Section 6.1.1 Study Setting – Updated wording to clarify that up to four sites internationally including sites in Malaysia and Kenya in the first instance - Section 6.1.2.1 Principal Investigator’s (PI) Qualifications and Agreements – Addition of wording to clarify that investigator(s) at International sites must be willing to comply with their protocol country- specific addendum and their country specific Good Clinical Practice. Addition of reference to the country-specific addendum. - Section 6.2 Site approval and activation – Addition of wording to clarify that International sites must conduct the trial in compliance with country-specific procedures and in accordance with local IRB/IEC and national regulatory authority requirements. Addition of reference to the country-specific addendum. - Section 6.3.1.1 Participant selection – Addition of wording to clarify that each country will adopt an approach that is appropriate for enrolment according to local conditions, and one that is in compliance with its IRB/REC and national regulatory authorities’ guidelines. Addition of reference to the country-specific addendum. - Section 6.3.1.3 Participant Exclusion Criteria – Addition of specification for exclusion criterion (7): Hospitalisation for isolation or infection control reasons will not considered an exclusion for this trial. - Section 6.3.1.6 Eligibility Criteria for Individuals Performing the Interventions – Addition of wording to clarify that International sites must conduct the trial in compliance with country-specific procedures and in accordance with local IRB/IEC and national regulatory authority requirements. Addition of reference to the country-specific addendum. - Section 6.3.1.8 Screening Procedures and Pre-randomisation Investigations – Addition of wording to clarify that International sites must be conduct screening procedures and pre-randomisation investigations in compliance with country-specific procedures and in accordance with local IRB/IEC and national regulatory authority requirements. Addition of wording for conduction of Screening/Baseline Visit (Day 1), Follow-up Visits (Days 7, 14 and 28) and Day 5 Telephone contact at international sites. - Section 6.4.2 Dispensing – Addition of wording to clarify that International sites must be conduct dispensing in compliance with country-specific procedures and in accordance with local IRB/IEC and national regulatory authority requirements. - Section 6.5.1 Schedule of samples – Addition of wording to clarify that stool samples will not be collected for International sites. - Section 6.5.2 Processing of samples –clarification that saliva and stool samples will be sent to Microbiology Department at Great Ormond Street Hospital (deletion of Prof Breuer). Addition of wording to clarify that country-specific procedures will be implemented in accordance with local IRB/IEC and national regulatory authority requirements for international sites. - Section 6.7 Participant timeline – Addition of wording to clarify that country-specific procedures will be implemented in accordance with local IRB/IEC and national regulatory authority requirements at International sites. - Section 6.7.3 Loss to Follow-up – Updated wording to clarify conduction of Day 5 visit at some international sites. - Section 6.8 Sample Size: Updated wording for clarity - Section 6.9.1 Recruitment – updated period of recruitment to 14 months and addition of international organisations.   Addition of wording to clarify that country-specific recruitment procedures will be implemented in accordance with local IRB/IEC and national regulatory authority requirements.   - Section 6.11.4.2 Statistical Methods – Outcomes: Site has been added as an adjusting factor in the primary model. - Section 6.11.4.4 Additional Analyses – Adjusted: Site has been added as an adjusting factor in the primary model. - Section 7.1 Ethics Committee Approval – Addition of wording to clarify that protocol will be submitted to the country-specific IRB/IEC and approvals will be required to be in place before the commencement of the trial. - Section 7.2 Competent Authority Approvals – Addition of wording to clarify that protocol will be submitted to the country-specific national regulatory authority and approvals will be required to be in place before the commencement of the trial. - Section 7.4 Protocol Amendments – Addition of wording to clarify that protocol will be submitted in accordance with local IRB/IEC and national regulatory authority requirements and approvals will be required to be in place before implementation at sites. - Section 7.5 Consent or Assent – Addition of wording to clarify that approved country-specific Participant Information Sheet (PIS) and Informed Consent Form (ICF) will be used to document consent procedure. - Section 7.8 Indemnity – Addition of wording to clarify country-specific reinsurance policy for international sites. - Section 7.12 Ancillary and Post-trial Care – Addition of wording to clarify that participants will be cared as country-specific healthcare system for international sites once completed the trial. - Section 11.6 Appendix 6 Country Specific Addendum added |
| 6.0 | 08-Jun-2021 | - Section 1.3 Structured trial summary, Exclusion criteria: Removed ‘Participants who have received the COVID-19 vaccine’ and added clarification to exclusion criterion 10 that ‘Participants that have received the COVID-19 vaccine can be included in this trial. - Section 1.3 Structured trial summary, Study type: Added minimisation factor for randomisation - ‘previous COVID-19 specific vaccination (Yes = ≥ 1 dose, No = no previous covid-19 specific vaccination)’. - Section 5.3 Trial Design: Added minimisation factor for randomisation - ‘previous COVID-19 specific vaccination (Yes = ≥ 1 dose, No = no previous covid-19 specific vaccination)’. - Section 6.3.1.3 Participant Exclusion Criteria: Removed exclusion criteria 11 ‘Participants who have received the COVID-19 vaccine’ and added clarification to exclusion criterion 10 that ‘Participants that have received the COVID-19 vaccine can be included in this trial. - Section 6.3.1.8 Screening Procedures and Pre-randomisation Investigations: Added ‘previous COVID-19 specific vaccination’ details as additional data collection items for pre-screening visit. - Section 6.9.1 Recruitment: Added clarification that potential participants will be approached via the COVID-19 national NHS Test and Trace Programme and clarification that other London or UK based NHS organisations will be approached if needed. - Section 6.10.1.1 Sequence generation: Added minimisation factor for randomisation - ‘previous COVID-19 specific vaccination (Yes = ≥ 1 dose, No = no previous covid-19 specific vaccination)’. - Section 6.11.4.3 Additional Analyses – Subgroup: Added ‘previous COVID-19 specific vaccination (Yes = ≥ 1 dose, No = no previous covid-19 specific vaccination)’. - Section 11.6 Appendix 6 Country Specific Addendum: Updated wording to the appendix to align with protocol. |

# References

^1^ Chan AW, Tetzlaff JM, Altman DG et Al. SPIRIT 2013 Statement: Defining Protocol Items for Clinical Trials. *Ann Intern Med* 2013; 158:200-207.

^2^ Chan AW, Tetzlaff, Gotzsche et Al. SPIRIT 2013 explanation and elaboration: guidance for protocols of clinical trials. *BMJ* 2013; 346: e7586.

^3^ <https://www.cdc.gov.tw/File/Get/ht8jUiB_MI-aKnlwstwzvw> (Favipiravir (Avigan®) tablets 200mg insert)

^4^ Chu 2004: Chu CM, Cheng VC, Hung IF, Wong MM, Chan KH, Chan KS, Kao RY, Poon LL, Wong CL, Guan Y, Peiris JS, Yuen KY; HKU/UCH SARS Study Group. Role of lopinavir/ritonavir in the treatment of SARS: initial virological and clinical findings. Thorax 2004; 59:252-6

^5^ Chan 2003: Chan KS, Lai ST, Chu CM, Tsui E, Tam CY, Wong MM, Tse MW, Que TL, Peiris JS, Sung J, Wong VC, Yuen KY. Treatment of severe acute respiratory syndrome with lopinavir/ritonavir: a multicentre retrospective matched cohort study. Hong Kong Med J. 2003 Dec;9(6):399-406.

^6^ Park 2019: Park SY, Lee JS, Son JS, Ko JH, Peck KR, Jung Y, et al. Post-exposure prophylaxis for Middle East respiratory syndrome in healthcare workers. J Hosp Infect. 2019 Jan;101(1):42–46.

^7^ Kim 2020: Kim KS, Ejima K, Ito Y, Iwanami S, Ohashi H, Koizumi Y, Asai Y, Nakaoka S, Watashi K, Thompson RN, Iwami S. Modelling SARS-CoV-2 Dynamics: Implications for Therapy. medRxiv 2020. 03.23.20040493; doi: <https://doi.org/10.1101/2020.03.23.20040493>

^8^ Gonçalves 2020: Gonçalves A, Bertrand J, Ke R, Comets E, de Lamballerie X, Malvy D, Pizzorno A, Terrier O, Calatrava MR, Mentré F, Smith P, Perelson AS, Guedj J.Timing of antiviral treatment initiation is critical to reduce SARS-CoV-2 viral load [submitted draft manuscript shared by authors]

^9^ Wölfel 2020: Wölfel R. et al. Virological assessment of hospitalized patients with COVID-2019 Nature (2020). <https://doi.org/10.1038/s41586-020-2196-x>

^10^ Ruis 2018: Ruis C, Brown LK, Roy S, Atkinson C, Williams R, Burns SO, Yara-Romero E, Jacobs M, Goldstein R, Breuer J, Lowe DM. Mutagenesis in Norovirus in Response to Favipiravir Treatment. N Engl J Med 2018; 379:2173-2176

^11^ Sissoko D, Laouenan C, Folkesson E, M'Lebing AB, Beavogui AH, Baize S*, et al.* Experimental Treatment with Favipiravir for Ebola Virus Disease (the JIKI Trial): A Historically Controlled, Single-Arm Proof-of-Concept Trial in Guinea. *PLoS Med* 2016,**13**:e1001967

^12^ PMDA 2014: 6. Japanese Pharmaceuticals and Medical Devices Agency (PMDA). Report on the Deliberation Results (English version) [Internet]. 4 Mar 2014 [last accessed 8/4/20]. Available: https://www.pmda.go.jp/files/000210319.pdf

^13^ Du 2020: Du YX, Chen XP. Favipiravir: Pharmacokinetics and Concerns About Clinical Trials for 2019‐nCoV Infection. Clinical Pharmacology and Therapeutics, First published: 04 April 2020 https://doi.org/10.1002/cpt.1844

^14^ Cai, Q. *et al*. Experimental treatment with Favipiravir for COVID‐19: an open‐label control study. *Engineering*. <https://doi.org/10.1016/j.eng.2020.03.007>.

^15^ Chen 2020: Chen C, Huang J, Cheng Z, Wu J, Chen S, Zhang Y, Chen B, Lu M, Luo Y, Zhang J, Yin P, Wang X. Favipiravir versus Arbidol for COVID-19: A Randomized Clinical Trial. medRxiv 2020.03.17.20037432; doi: https://doi.org/10.1101/2020.03.17.20037432

^16^ Cao 2020: Cao B, Wang Y, Wen D, Liu W, Wang J, Fan G, Ruan L, Song B, Cai Y, Wei M, Li X, Xia J, Chen N, Xiang J, Yu T, Bai T, Xie X, Zhang L, Li C, Yuan Y, Chen H, Li H, Huang H, Tu S, Gong F, Liu Y, Wei Y, Dong C, Zhou F, Gu X, Xu J, Liu Z, Zhang Y, Li H, Shang L, Wang K, Li K, Zhou X, Dong X, Qu Z, Lu S, Hu X, Ruan S, Luo S, Wu J, Peng L, Cheng F, Pan L, Zou J, Jia C, Wang J, Liu X, Wang S, Wu X, Ge Q, He J, Zhan H, Qiu F, Guo L, Huang C, Jaki T, Hayden FG, Horby PW, Zhang D, Wang C. A Trial of Lopinavir-Ritonavir in Adults Hospitalized with Severe Covid-19. N Engl J Med 2020. doi: 10.1056/NEJMoa2001282

^17^ [http://www.hma.eu/fileadmin/dateien/Human_Medicines/01-About_HMA/Working_Groups/CTFG/2014_09_HMA_CTFG_Contraception.pdf](https://urldefense.proofpoint.com/v2/url?u=http-3A__www.hma.eu_fileadmin_dateien_Human-5FMedicines_01-2DAbout-5FHMA_Working-5FGroups_CTFG_2014-5F09-5FHMA-5FCTFG-5FContraception.pdf&d=DwMGaQ&c=bXyEFqpHx20PVepeYtwgeyo6Hxa8iNFcGZACCQj1uNM&r=oAAR0oHDdzIKflCkXFVIlbpPttULuMBvFtpFGgjWBIQ&m=LXAkblVq2Le3u9pir38_7jBW6s4BnYq3wSomAglN4uE&s=f6B2iDXK1XF5dyxCHUAJKfvlt2ngJYZbMWSNF7zVCKs&e=)]

# 11 Appendices

## 11.1 Appendix 1 Figures referred to in the text


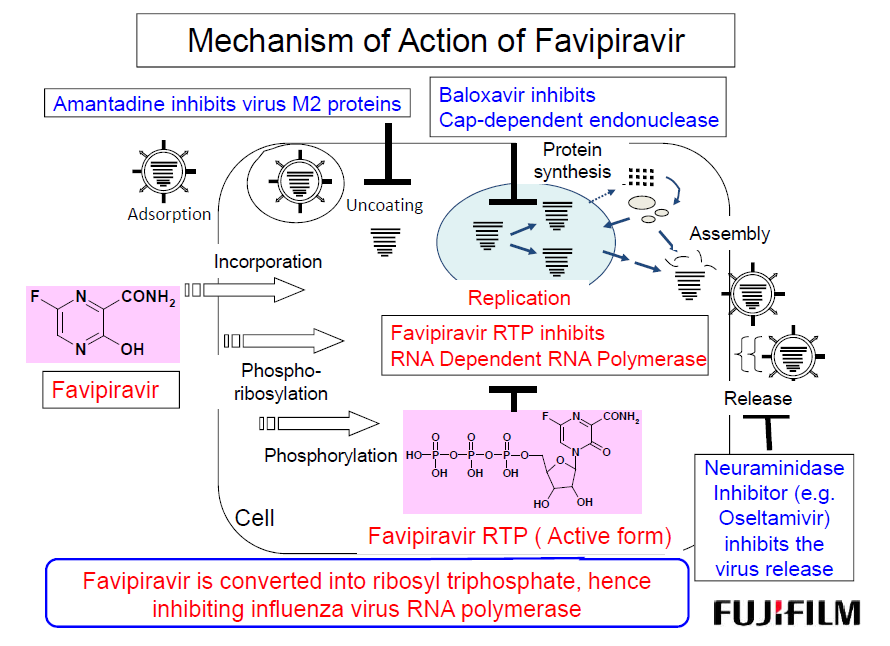


**Figure 1.** Mechanism of action of Favipiravir. Data provided by Fujifilm.

**Figure 2.** Potential spectrum of activity of favipiravir. Data provided by Fujifilm.

**Figure 3.** Pharmacokinetic analysis of favipiravir administered at the doses proposed for the current trial. Data provided by Fujifilm.

**A**

**B**

**Figure 4.** Changes in (A) serum uric acid during treatment with favipiarivir versus placebo and (B) serum alanine aminotransferase per patient during treatment with favipiravir. Data from JP120 study in healthy adult males aged 20-39 years. Data provided by Fujifilm.

## 11.2 Appendix 2 Drug ingredients and excipients

1. **Lopinavir/ritonavir (Kaletra):**

| **Active ingredients** | **200 mg of lopinavir co-formulated with 50 mg of ritonavir** |
| --- | --- |
| **Excipients** | **Copovidone**  **Sorbitan laurate**  **Colloidal anhydrous silica**  **Sodium stearyl fumarate**  **Hypromellose**  **Titanium dioxide**  **Macrogols type 400 (Polyethylene glycol 400)**  **Hydroxypropyl cellulose**  **Talc**  **Colloidal anhydrous silica**  **Macrogols type 3350 (Polyethylene glycol 3350)**  **Yellow ferric oxide E172**  **Polysorbate 80** |

1. **Favipiravir:**

| **Active ingredients** | **Favipiravir 200mg** |
| --- | --- |
| **Excipients** | **Povidone**  **Colloidal silicon dioxide**  **Low-substituted hydroxypropyl cellulose**  **Crospovidone**  **Sodium stearyl fumarate**  **Hypromellose**  **Titanium dioxide**  **Talc**  **Yellow ferric oxide** |

## 11.3 Appendix 3 Drug interactions and contraindications

A comprehensive list of drug interactions and contraindications for Favipiravir and Lopinavir/ritonavir (Kaletra) can be found at the following link:

<https://liverpool-covid19.s3.eu-west-2.amazonaws.com/landing-page/Covid_InteractionSummary_Web_2020_Mar12.pdf>

1. **Lopinavir/ritonavir (Kaletra)**

Kaletra contains lopinavir and ritonavir, both of which are inhibitors of the P450 isoform CYP3A. Kaletra should not be co-administered with medicinal products that are highly dependent on CYP3A for clearance and for which elevated plasma concentrations are associated with serious and/or life threatening events. These medicinal products include:

| **Medicinal product class** | **Medicinal products within class** | **Rationale** | |
| --- | --- | --- | --- |
| Concomitant medicinal product levels increased | | | |
| Alpha_1_-adrenoreceptor antagonist | Alfuzosin | Increased plasma concentrations of alfuzosin which may lead to severe hypotension. The concomitant administration with alfuzosin is contraindicated. | |
| Antianginal | Ranolazine | Increased plasma concentrations of ranolazine which may increase the potential for serious and/or life-threatening reactions. |  |
| Antiarrhythmics | Amiodarone, dronedarone | Increased plasma concentrations of amiodarone and dronedarone. Thereby, increasing the risk of arrhythmias or other serious adverse reactions. | |
| Antibiotic | Fusidic Acid | Increased plasma concentrations of fusidic acid. The concomitant administration with fusidic acid is contraindicated in dermatological infections. | |
| Anticancer | Neratinib | Increased plasma concentrations of neratinib which may increase the potential for serious and/or life-threatening reactions. |  |
|  | Venetoclax | Increased plasma concentrations of venetoclax. Increased risk of tumor lysis syndrome at the dose initiation and during the ramp-up phase. |  |
| Anti-gout | Colchicine | Increased plasma concentrations of colchicine. Potential for serious and/or life-threatening reactions in patients with renal and/or hepatic impairment. | |
| Antihistamines | Astemizole, terfenadine | Increased plasma concentrations of astemizole and terfenadine. Thereby, increasing the risk of serious arrhythmias from these agents. | |
| Antipsychotics/ Neuroleptics | Lurasidone | Increased plasma concentrations of lurasidone which may increase the potential for serious and/or life-threatening reactions. | |
|  | Pimozide | Increased plasma concentrations of pimozide. Thereby, increasing the risk of serious haematologic abnormalities, or other serious adverse effects from this agent. | |
|  | Quetiapine | Increased plasma concentrations of quetiapine which may lead to coma. The concomitant administration with quetiapine is contraindicated. | |
| Ergot alkaloids | Dihydroergotamine, ergonovine, ergotamine, methylergonovine | Increased plasma concentrations of ergot derivatives leading to acute ergot toxicity, including vasospasm and ischaemia. | |
| GI motility agent | Cisapride | Increased plasma concentrations of cisapride. Thereby, increasing the risk of serious arrhythmias from this agent/ | |
| Hepatitis C virus direct acting antivirals | Elbasvir/grazoprevir | Increased risk of alanine transaminase (ALT) elevations. |  |
|  | Ombitasvir/paritaprevir/ritonavir with or without dasabuvir | Increased plasma concentrations of paritaprevir; thereby, increasing the risk of alanine transaminase (ALT) elevations. |  |
| Lipid-modifying agents  HMG Co-A Reductase Inhibitors | Lovastatin, simvastatin | Increased plasma concentrations of lovastatin and simvastatin; thereby, increasing the risk of myopathy including rhabdomyolysis. |  |
| Microsomal triglyceride transfer protein (MTTP) inhibitor | Lomitapide | Increased plasma concentrations of lomitapide. |  |
| Phosphodiesterase (PDE5) inhibitors | Avanafil | Increased plasma concentrations of avanafil. | |
|  | Sildenafil | Contraindicated when used for the treatment of pulmonary arterial hypertension (PAH) only. Increased plasma concentrations of sildenafil. Thereby, increasing the potential for sildenafil-associated adverse events (which include hypotension and syncope). | |
|  | Vardenafil | Increased plasma concentrations of vardenafil. | |
| Sedatives/hypnotics | Oral midazolam, triazolam | Increased plasma concentrations of oral midazolam and triazolam. Thereby, increasing the risk of extreme sedation and respiratory depression from these agents. | |
| **Lopinavir/ritonavir medicinal product level decreased** | | | |
| Herbal products | St. John's wort | Herbal preparations containing St John's wort (*Hypericum perforatum)* due to the risk of decreased plasma concentrations and reduced clinical effects of lopinavir and ritonavir. | |

Other interactions with medicinal products

Strong CYP3A4 inhibitors such as protease inhibitors may increase **bedaquiline** exposure which could potentially increase the risk of bedaquiline-related adverse reactions. Therefore, combination of bedaquiline with lopinavir/ritonavir should be avoided. However, if the benefit outweighs the risk, co-administration of bedaquiline with lopinavir/ritonavir must be done with caution. More frequent electrocardiogram monitoring and monitoring of transaminases is recommended (refer to the bedaquiline SmPC).

Co-administration of **delamanid** with a strong inhibitor of CYP3A (as lopinavir/ritonavir) may increase exposure to delamanid metabolite, which has been associated with QTc prolongation. Therefore, if co-administration of delamanid with lopinavir/ritonavir is considered necessary, very frequent ECG monitoring throughout the full delamanid treatment period is recommended (refer to the delamanid SmPC).

Life-threatening and fatal drug interactions have been reported in patients treated with colchicine and strong inhibitors of CYP3A like ritonavir. Concomitant administration with **colchicine is contraindicated in patients with renal and/or hepatic impairment**.

The combination of Kaletra with:

- **tadalafil**, indicated for the treatment of pulmonary arterial hypertension, is not recommended;

- **riociguat** is not recommended;

- **vorapaxar** is not recommended;

- **fusidic acid in osteo-articular infections** is not recommended;

- **salmeterol** is not recommended;

- **rivaroxaban** is not recommended.

The combination of Kaletra with **atorvastatin** is not recommended. If the use of atorvastatin is considered strictly necessary, the lowest possible dose of atorvastatin should be administered with careful safety monitoring. Caution must also be exercised and reduced doses should be considered if Kaletra is used concurrently with rosuvastatin. If treatment with a HMG-CoA reductase inhibitor is indicated, pravastatin or fluvastatin is recommended.

*PDE5 inhibitors*

Particular caution should be used when prescribing **sildenafil** or **tadalafil** for the treatment of erectile dysfunction in patients receiving Kaletra. Co-administration of Kaletra with these medicinal products is expected to substantially increase their concentrations and may result in associated adverse events such as hypotension, syncope, visual changes and prolonged erection. Concomitant use of avanafil or vardenafil and lopinavir/ritonavir is contraindicated. Concomitant use of sildenafil prescribed for the treatment of pulmonary arterial hypertension with Kaletra is contraindicated.

Particular **caution** must be used when prescribing Kaletra and medicinal products known to induce QT interval prolongation such as: **chlorpheniramine, quinidine, erythromycin, clarithromycin**. Indeed, Kaletra could increase concentrations of the co-administered medicinal products and this may result in an increase of their associated cardiac adverse reactions. Cardiac events have been reported with Kaletra in preclinical studies; therefore, the potential cardiac effects of Kaletra cannot be currently ruled out.

Co-administration of Kaletra with **rifampicin** is not recommended. Rifampicin in combination with Kaletra causes large decreases in lopinavir concentrations which may in turn significantly decrease the lopinavir therapeutic effect. Adequate exposure to lopinavir/ritonavir may be achieved when a higher dose of Kaletra is used but this is associated with a higher risk of liver and gastrointestinal toxicity. Therefore, this co-administration should be avoided unless judged strictly necessary.

Concomitant use of Kaletra and **fluticasone or other glucocorticoids that are metabolised by CYP3A4, such as budesonide and triamcinolone**, is not recommended unless the potential benefit of treatment outweighs the risk of systemic corticosteroid effects, including Cushing's syndrome and adrenal suppression.

**2) Favipiravir**

To date, studies that have been completed include acetaminophen, hydrazine, norethindrone-ethinyl estradiol contraceptive (Ortho-Novum®), oseltamivir, pyrazinamine, and raloxifene. Except as noted, favipiravir co- administration was well tolerated and did not significantly alter the PK of either agent or in a clinically important manner.

**Paracetamol (Acetaminophen)**: Favipiravir in combination with paracetamol (acetaminophen) increased acetaminophen blood levels 14 to 17% based on plasma AUC comparisons.

Maximum permitted daily dose of paracetamol: 3000mg (max. 6 tablets in 24 hours).

**Hydralazine:** Co-administration of favipiravir and hydralazine resulted in a 13% reduction in hydralazine AUC. No changes in favipiravir PK were observed.

**Ortho Novum® 1/35 (norethindrone and ethinyl estradiol):** Favipiravir co-administration increased both norethindrone and ethinyl estradiol blood levels. Norethindrone plasma AUC increased 47% and ethinyl estradiol plasma AUC increased 43%. One subject discontinued the study due to transient, mildly elevated ALT (2.8 × normal) and AST (1.7 × normal).

**Oseltamivir:** Favipiravir did not alter the PK of oseltamivir nor did oseltamivir alter favipiravir PK.

**Raloxifene**: Co-administration of favipiravir with raloxifene, a potent aldehyde oxidase inhibitor, did not appreciably alter favipiravir PK. Favipiravir plasma AUC was reduced 15% when administered in combination with raloxifene.

**Repaglinide**: Favipiravir administration with repaglinide, an anti-diabetic agent that is extensively metabolized by CYP2C8 and CYP3A4, increased repaglinide plasma AUC 30 to 50% due to inhibition of CYP2C8.

**Pyrazinamide**: Pyrazinamide administration with favipiravir examined possible renal urate transporter interactions. Pyrazinamide increased blood uric acid levels 2 to 9 mg/dL over baseline. The addition of favipiravir increased blood uric acid levels 4 to 11 mg/dL over baseline, indicating a moderate additive effect. One subject developed headache and fever in association with elevated liver function tests (AST, ALT, gamma-glutamyl transferase, and lactate dehydrogenase) following pyrazinamide and favipiravir co-administration, which was determined to be a serious adverse event (SAE) and resolved upon discontinuation.

## 11.4 Appendix 4 Tabulated list of adverse reactions associated with the trial IMPs

1. **Lopinavir/ritonavir**

*Adverse reactions from clinical trials and post-marketing experience in adult patients:*

The following events have been identified as adverse reactions. The frequency category includes all reported events of moderate to severe intensity, regardless of the individual causality assessment. The adverse reactions are displayed by system organ class. Within each frequency grouping, undesirable effects are presented in order of decreasing seriousness: very common (≥ 1/10), common (≥ 1/100 to < 1/10), uncommon (≥ 1/1000 to < 1/100) and rare (≥1/10,000 to <1/1000).

| **Undesirable effects in clinical studies and post-marketing in adult patients** | | |
| --- | --- | --- |
| **System organ class** | **Frequency** | **Adverse reaction** |
| Infections and infestations | Very common | Upper respiratory tract infection |
|  | Common | Lower respiratory tract infection, skin infections including cellulitis, folliculitis and furuncle |
| Blood and lymphatic system disorders | Common | Anaemia, leucopenia, neutropenia, lymphadenopathy |
| Immune system disorders | Common | Hypersensitivity including urticaria and angioedema |
|  | Uncommon | Immune reconstitution inflammatory syndrome |
| Endocrine disorders | Uncommon | Hypogonadism |
| Metabolism and nutrition disorders | Common | Blood glucose disorders including diabetes mellitus, hypertriglyceridaemia, hypercholesterolemia, weight decreased, decreased appetite |
|  | Uncommon | Weight increased, increased appetite |
| Psychiatric disorders | Common | Anxiety |
|  | Uncommon | Abnormal dreams, libido decreased |
| Nervous system disorders | Common | Headache (including migraine), neuropathy (including peripheral neuropathy), dizziness, insomnia |
|  | Uncommon | Cerebrovascular accident, convulsion, dysgeusia, ageusia, tremor |
| Eye disorders | Uncommon | Visual impairment |
| Ear and labyrinth disorders | Uncommon | Tinnitus, vertigo |
| Cardiac disorders | Uncommon | Atherosclerosis such as myocardial infarction, atrioventricular block, tricuspid valve incompetence |
| Vascular disorders | Common | Hypertension |
|  | Uncommon | Deep vein thrombosis |
| Gastrointestinal disorders | Very common | Diarrhoea, nausea |
|  | Common | Pancreatitis^1^, vomiting, gastrooesophageal reflux disease, gastroenteritis and colitis, abdominal pain (upper and lower), abdominal distension, dyspepsia, haemorrhoids, flatulence |
|  | Uncommon | Gastrointestinal haemorrhage including gastrointestinal ulcer, duodenitis, gastritis and rectal haemorrhage, stomatitis and oral ulcers, faecal incontinence, constipation, dry mouth |
| Hepatobiliary disorders | Common | Hepatitis including AST, ALT and GGT increases |
|  | Uncommon | Jaundice, hepatic steatosis, hepatomegaly, cholangitis, hyperbilirubinemia |
| Skin and subcutaneous tissue disorders | Common | Rash including maculopapular rash, dermatitis/rash including eczema and seborrheic dermatitis, night sweats, pruritus |
|  | Uncommon | Alopecia, capillaritis, vasculitis |
|  | Rare | Stevens-Johnson syndrome, erythema multiforme |
| Musculoskeletal and connective tissue disorders | Common | Myalgia, musculoskeletal pain including arthralgia and back pain, muscle disorders such as weakness and spasms |
|  | Uncommon | Rhabdomyolysis, osteonecrosis |
| Renal and urinary disorders | Uncommon | Creatinine clearance decreased, nephritis, haematuria |
| Reproductive system and breast disorders | Common | Erectile dysfunction, menstrual disorders - amenorrhoea, menorrhagia |
| General disorders and administration site conditions | Common | Fatigue including asthenia |

1. **Favipiravir**

*Adverse reactions from clinical trials in adult patients:*

**
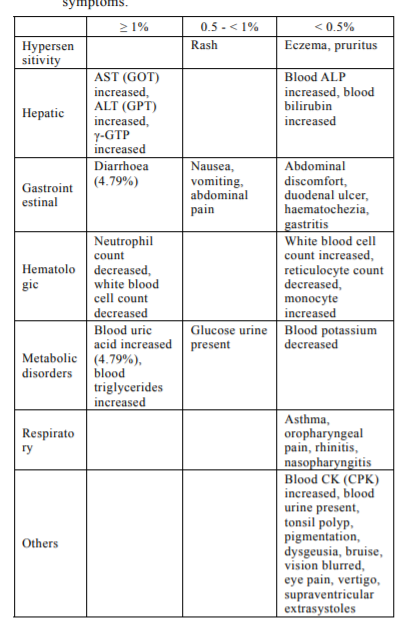
**

## 11.5 Appendix 5 PPE Guidance (Applicable for the UK sites only)

<https://assets.publishing.service.gov.uk/government/uploads/system/uploads/attachment_data/file/878750/T2_poster_Recommended_PPE_for_primary__outpatient__community_and_social_care_by_setting.pdf>


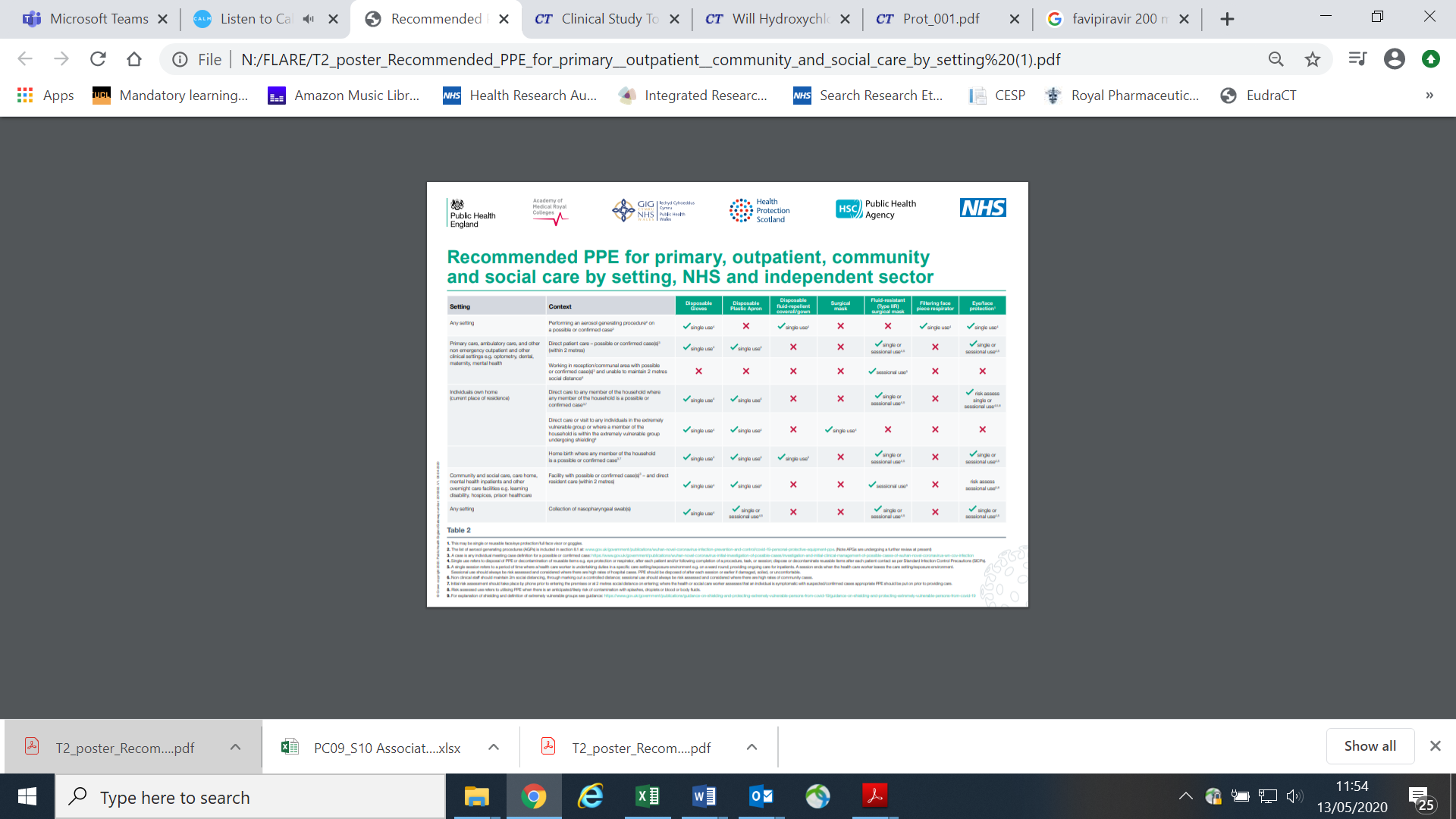


## Appendix 6 COUNTRY-SPECIFIC ADDENDUM

Please refer to FLARE Protocol Appendix 6 Country-specific Addendum.

1. Day 14 safety blood tests will not be repeated if no significantly abnormal safety blood tests results were observed on Day 7. [↑](#footnote-ref-2)
2. As Participants randomised on Mondays will not be able to attend their Day 7 visit on Sundays, PK samples will not be collected for these participants. [↑](#footnote-ref-3)
3. Initial eligibility assessment and participant verbal confirmation. [↑](#footnote-ref-4)
4. Research staff to contact participant within 6 hours of safety bloods results if participant not eligible. Stop IMP intake immediately, AEs/SAEs check and arrange IMP kit collection within 24 hours. Tablets count on receipt at site. [↑](#footnote-ref-5)
5. There will be flexibility for Day 7 (+1 day) and Day 14 visits (+ 3 days), and Day 28 Follow-up (+/- 3 days) [↑](#footnote-ref-6)
6. This visit will take place at the participant’s home or on-site (in designated hospital area) [↑](#footnote-ref-7)
7. Day 7 and Day 14 visits can take place in clinic if the participant has completed their isolation period. [↑](#footnote-ref-8)
8. Participant eligibility (based on pre-screening telephone contact assessment of inclusion and exclusion criteria) must be checked again at Screening/Baseline visit (Day 1) prior to randomisation. [↑](#footnote-ref-9)
9. Including age, sex and ethnicity, smoking status, height & body weight [↑](#footnote-ref-10)
10. Demographics collected at pre-screening will only be checked and re-confirmed at Screening/Baseline visit. [↑](#footnote-ref-11)
11. Women of childbearing potential (WOCBP) only: urine pregnancy test at screening/Baseline visit (Day 1) before randomisation, and at least 7 days after their last dose of IMP (last day of treatment). [↑](#footnote-ref-12)
12. including temperature, pulse rate and respiratory rate [↑](#footnote-ref-13)
13. Telephone contact to remind participant to collect Day 5 sample, arrange time visit for Day 7 visit and instructions for IMP intake time on Day 7, check for AEs and SAEs. Where Day 5 is scheduled to fall over a weekend, telephone contact may be performed on a Friday. [↑](#footnote-ref-14)
14. One diagnostic swab will be taken at baseline unless already taken separately at testing site. [↑](#footnote-ref-15)
15. Daily saliva sample for viral load measurement/viral sequencing. Collected by trial staff on Day 1. Self-collected by participants Day 2 to Day 7. [↑](#footnote-ref-16)
16. Self-recorded 4 times daily by participants (except for Day 1 where only two temperature measurements will be recorded and first recording will be done by trial staff, and Day 7 where only one or two temperature measurements are expected to be recorded before diary collected by trial staff)*.* [↑](#footnote-ref-17)
17. Results will be assessed as soon as available (within 24 hours) and ineligible participant contacted within 6 hours from results receipt for immediate discontinuation of trial medication intake. [↑](#footnote-ref-18)
18. Day 14 safety blood tests will not be repeated if no abnormal safety blood tests results were observed on Day 7. [↑](#footnote-ref-19)
