# Supplementary material for: Favipiravir, lopinavir-ritonavir, or combination therapy (FLARE): A randomised, double-blind, 2 × 2 factorial placebo-controlled trial of early antiviral therapy in COVID-19
Source: PLoS Med. 2022 Oct 19;19(10):e1004120. doi: 10.1371/journal.pmed.1004120 (PMC9629589; doi:10.1371/journal.pmed.1004120)
Supplement: Attachment — Submitted filename: Response to reviewers 170822.docx [file pmed.1004120.s018.docx]

**Response to editorial and reviewer comments**

**PMEDICINE-D-22-01160R1; Favipiravir, lopinavir-ritonavir or combination therapy (FLARE): a randomised, double blind, 2x2 factorial placebo-controlled trial of early antiviral therapy in COVID-19**

**Editorial Comments**

In addition, we request that you upload any figures associated with your paper as individual TIF or EPS files with 300dpi resolution at resubmission

Thank-you – we have included the figures as individual TIF files with 300dpi resolution.

We ask every co-author listed on the manuscript to fill in a contributing author statement, making sure to declare all competing interests. If any of the co-authors have not filled in the statement, we will remind them to do so when the paper is revised.

We believe that all authors have completed the statement but please inform us if this is not the case.

Please ensure that the paper adheres to the PLOS Data Availability Policy (see <http://journals.plos.org/plosmedicine/s/data-availability)>, which requires that all data underlying the study's findings be provided in a repository or as Supporting Information. For data residing with a third party, authors are required to provide instructions with contact information for obtaining the data. PLOS journals do not allow statements supported by "data not shown" or "unpublished results." For such statements, authors must provide supporting data or cite public sources that include it.

With the additional tables requested by reviewers (see below), all relevant data are in the manuscript and supporting information. Patient level data is available from the authors upon reasonable request. This is in line with the informed consent form for this study which included the following statements:

“I agree that my data gathered in this trial will be stored in a secure facility (with limited access by individuals from the sponsor’s office (University College London) for 10 years after the completion of the trial as set out by the UK Medicines & Healthcare products Regulatory Agency.”

“I give consent for my data collected as part of the trial to be shared with other researchers and used in other ethically approved research following legal requirements to conceal my identity.”

Please complete the CONSORT checklist and ensure that all components of CONSORT are present in the manuscript, including [how randomization was performed, allocation concealment, blinding of intervention, definition of lost to follow-up, power statement]. When completing the checklist, please use section and paragraph numbers, rather than page numbers.

We have completed the CONSORT checklist and included this in Supplementary Information.

A pre-specified statistical analysis plan is mentioned in the methods section (line 183). Please include the relevant prospectively written document with your revised manuscript as a Supporting Information file to be published alongside your study, if accepted and cite it in the Methods section. A legend for this file should be included at the end of your manuscript.  Please also include the study protocol document.

We have included the Statistical Analysis Plan and Protocol as Supporting Information.

Please remove COI/funding source/ info from the end of the manuscript and instead include in the manuscript submission form

This has been removed from the manuscript.

ABSTRACT
Please structure your abstract using the PLOS Medicine headings (Background, Methods and Findings, Conclusions). Please combine the Methods and Findings sections into one section, “Methods and findings”.
In the last sentence of the Abstract Methods and Findings section, please describe the main limitation(s) of the study's methodology.

We have restructured the abstract as required and added a sentence to describe the major limitation: “The major limitation was that the study population was relatively young and healthy compared to those most affected by the COVID-19 pandemic.”

At this stage, we ask that you include a short, non-technical Author Summary of your research to make findings accessible to a wide audience that includes both scientists and non-scientists. The Author Summary should immediately follow the Abstract in your revised manuscript. This text is subject to editorial change and should be distinct from the scientific abstract. Please see our author guidelines for more information: <https://journals.plos.org/plosmedicine/s/revising-your-manuscript#loc-author-summary>

Thank-you, we have included an author summary as requested.

METHODS and FINDINGS
When a p value is given, please specify the statistical test used to determine it.

We have added a sentence to clarify the test used in comparing categorical outcomes (Lines 223-4). All other methods used to obtain estimates and p-values are defined in the Statistical analysis section.

FIGURES & TABLES
Please consider avoiding the use of red and green in order to make your figures more accessible to those with colour blindness

Thank-you for this suggestion. One of the senior authors is red-green colour blind and has reviewed the figures and is satisfied with the interpretability. We have included different types of line (eg solid, dashed) to improve clarity.

Please define the abbreviations in Figure 3 (VL), figure 4 (EC50, EC90), suppl figure 5 (LPV/r), supp figure 6 (the treatment arms are labelled differently than other figures/tables throughout the manuscript i.e. favipira Vs favipiravir. Please ensure congruency and include abbreviations as necessary)

Thank-you. We have defined the abbreviations as requested and have updated Supplementary Figure 6 to be consistent with other tables and figures.

Please define the abbreviation in table 2 (BMI), table 3 (ITT, CI), table 4 (CI), table 5 (CI), supp table 2 (IQR), supp table 3 (Pk)
Please indicate in the figure caption the meaning of whiskers in Figure 4
Please indicate in the figure caption the meaning of whiskers in supp figure 6

We have defined the abbreviations as requested. Whiskers represent 1.5*IQR and we have updated the figure captions to explain this.

DISCUSSION
Please present and organize the Discussion as follows: a short, clear summary of the article's findings; what the study adds to existing research and where and why the results may differ from previous research; strengths and limitations of the study; implications and next steps for research, clinical practice, and/or public policy; one-paragraph conclusion.

We have reorganised the order of paragraphs in the Discussion and hope that we now meet these requirements.

Comments from the reviewers:

**Reviewer #1: Statistical Review**

Lowe and colleagues present the results of a 2x2 factorial designed study of early antiviral treatment in COVID-19. This review considers the use of statistics in the paper.

Overall, these are very good, but I do have a few minor comments.

The modelling approach is reasonable, and the authors apply a number of sensitivity analyses, but I would like to have seen the results of models fitted without interaction terms; this could be done in a supplementary table.

We thank the reviewer for the comment. The trial was powered to look at the two active treatments separately as well as in combination. The interaction term is a key feature of the factorial design. It would not be possible to obtain the intended interpretations of the results without having the interaction term in the model. However, we have presented the suggested model in the table below for information.

|  | **N** | **Favipiravir** | | **LPV/r** | |  |
| --- | --- | --- | --- | --- | --- | --- |
|  |  | Coefficient (95% CI) | p-value | Coefficient (95% CI) | p-value |  |
|  |  |  |  |  |  |  |
|  |  |  |  |  |  |  |
| ITT population | 224 | -0.28 (-0.73, 0.18) | 0.23 | 0.11 (-0.34, 0.57) | 0.63 |  |

In the text, the primary analysis results are reported as reductions in the treated arms relative to placebo/placebo, though the confidence intervals quoted are for the differences (i.e. the opposite sign to the point estimates), which is slightly confusing.

We agree with the reviewer and have changed the text to minimise confusion. The new text reads as follows: “change in viral load versus placebo for favipiravir monotherapy -0.57 log_10_ copies/mL (95% confidence interval (CI) -1.21 to 0.07, p=0.08), for lopinavir-ritonavir monotherapy -0.18 log_10_ copies/mL (95% CI -0.82 to 0.46, p=0.58).”

The main conclusion that there is no clear evidence that either treatment produces clinically relevant reductions in viral load, seems a little negative. A clinically important reduction is quoted as being in the range 0.5 to 1.0 log10 copies per ml, and the main effect of favipiravir is 0.57 in the primary analysis, or more in the supporting analyses, and much more in the subgroup with higher baseline viral load. Saying that, the authors do discuss these points.

We have removed the word ‘modestly’ in reference to significant results from our previous Discussion. However, we believe that our current interpretation is the fairest presentation of the results and readers will be able to reach their own conclusions from the data.

There is mention of a pre-specified statistical analysis plan, but it is not referenced, and not included in the supplementary materials.

As detailed above, we have now included this in Supplementary material.

**Reviewer #2:**

Lowe et al report the results of a blinded randomised clinical trial for COVID patients of favipiravir, LPV/r, the combination, and placebo. The primary outcome was salivary viral load at day 5 (adjusted for baseline level). This was a phase 2a trial. There was no statistically significant change in viral load compared to placebo for any of the treatment arms in the primary analysis.

Overall, this is a well written manuscript that reports on the trial as planned in the trial protocol and as registered on clinicaltrials.gov. The use of placebo is a particular strength. It provides an example of a well conducted phase 2 trial that can inform the design of future trials. As the authors state in the discussion more trials such as this may have helped to guide which agents should be included in larger phase 3 trials during a pandemic. Unfortunately, the time taken to complete this study also provides an example that such earlier phase trials need to be conducted and reported quickly to make such an impact. The reality is that the results from FLARE are unlikely to have great impact on the therapeutic landscape or subsequent trials given the alternative options in mid 2022 - at least in high income countries. One suggestion is that in the discussion the authors could consider the issue of whether favipiravir should still be used or trialled in lower middle income countries that may not have access to monoclonals or paxlovid and molnupiravir.

We thank the reviewer for their generous comments about the study. We accept that the results will be reported later than we had originally hoped. However, manufacture of placebos and regulatory approval had been completed by September 2020 with the study opened for recruitment in the same month. Multiple sites expressed interest and we believe the trial could have been completed rapidly (with additional drugs and combinations assessed subsequently to inform optimal choices for Phase III). However, studies in the UK were subjected to a prioritisation process and NHS sites were required to prioritise only those with ‘Urgent Public Heath’ status. FLARE was not awarded this badging and, as a result, could only proceed at two sites with most recruitment at a single site.

We accept that the results may now be of more relevance to low- and middle-income countries and we have emphasised this point in the Discussion, adding the following sentence: “However, they retain importance for low- and middle-income countries where favipiravir is already in routine use and where molnupiravir or nirmatrelvir-ritonavir may be prohibitively expensive.”

I would like to see details on whether all randomised participants had confirmed SARS-CoV-2 - the first inclusion criteria appears to only require consistent symptoms. The protocol suggests that they all were swabbed for a diagnosis, but I think they could be randomised prior to the results. Can probably infer this from the figures, but should report at start of results.

Three patients were included in the trial on the basis of this inclusion criteria – i.e. symptoms compatible with COVID-19 disease (Fever >37.8C on at least one occasion AND either cough and/ or anosmia) within the first 5 days of symptom onset. Two of these patients had detectable virus at baseline (viral load 4.51 and 6.20 log10) but one had a negative baseline sample. We have added a comment to explain this.

In terms of the reporting of the primary outcome, I would have liked to have seen more detail in the main manuscript. Specifically in table 3, reporting the coefficient was not intuitively understood. To me it would be clearer to see the baseline and day 5 viral loads (I know this is in figure 2, but the summary mean numbers could be reported in the text or table) in each of the 4 groups, the change in viral load in the 4 groups, and then the coefficient which represents the difference in change between the 3 active groups and the placebo group (if I have understood the analysis).

We have added the data underlying Figure 2 as a new Supplementary Table 1.

Figure 2 and 3 and suppl figure 2 are certainly helpful as well. Table 4 is also helpful in seeing the raw numbers in terms of numerator and denominator. At line 234-235, I'm not sure it is appropriate to say the pre-defined threshold for significance. I haven't seen the Statistical analysis plan, but I would have thought the pre-defined threshold related to the primary analysis, and not to the
various additional analyses that were performed. I think better to just state the numbers. Same point when this is discussed in the discussion at line 313-314.

We thank the reviewer for this point. As mentioned earlier, we have now included the Statistical Analysis Plan as Supplementary information. We have removed reference to the ‘pre-specified’ significance threshold.

At line 240-245, was this 'post-hoc supportive analysis' in pre-defined sub-groups? Would be good to be clear about that. E.g, 'In the post-hoc supportive analysis (i.e., this was not a pre-defined sub-group analysis) …'

Thank-you – we have added the clarification as suggested: “In a post-hoc supportive analysis (i.e. these were not pre-defined subgroups)…”

For clarity and ease of use of data for meta-analyses etc, I suggest all the secondary outcomes be reported in a table (excluding the PK-PD modelling aspects). I can get this data from the manuscript text, but I would encourage clear reporting of all the secondary outcomes.

We thank the reviewer for this suggestion and have included duration of fever and rates of hospitalisation, ITU admission and death in a new Supplementary Table 5

Line 324 - the clinical trial results for molnupiravir have now been reported.

The molnupiravir trial results were available at the time of manuscript preparation and are included as Reference 2. However, the original trial was conducted in unvaccinated patients and the effect was relatively modest (especially in the latter part of the trial) and we therefore believe that ‘real-world’ clinical efficacy is not guaranteed. Ongoing trials such as PANORAMIC seek to ascertain this.

Line 354-358 - rather than using 'this' in some of the sentences, it would be clearer to directly refer to 'LPV-ritonavir'.

Thank-you – we have changed ‘this drug’ to lopinavir-ritonavir.

Line 369-378 - the paragraph could come earlier in the discussion. I suggest grouping all the favipiravir paragraphs together.

We have reordered the Discussion as suggested.

Limitations wise - a discussion of timeliness of results and therefore ability to impact on subsequent trials or practice could be considered in this section.

We have added a statement as follows: “Due to the lack of UK Urgent Public Health status, recruitment took longer than anticipated and the results of FLARE are available only after the identification of other oral antiviral agents for COVID-19.”

**Reviewer #3:**

The authors report on the FLARE study which was  a Phase 2, proof of principle, randomised, placebo-controlled, 2x2 factorial, double-blind trial of outpatients with early COVID-19 (within 7 days of symptom onset) at two sites in the United Kingdom. The study consisted of 4 arms;
1)      favipiravir + lopinavir-ritonavir.
2)      favipiravir plus lopinavir-ritonavir placebo.
3)      lopinavir-ritonavir plus favipiravir placebo.
4)      both placebos.

The primary outcome was viral load relative to baseline on day 5.

Abstract
The favipiravir alone arm demonstrated the greatest reductions in viral load but the confidence intervals were large ( 1.21 to 0.07 log10 reductions, p=0.08) leading to a non significant result.

Methods
The patient population are patients diagnosed with coronavirus within 7 days of symptom onset who were managed in the outpatient setting. The authors state ambulatory patients in the methods and then in exclusion mention participants were being treated as a hospital inpatient for any condition. They should just make this clear in the abstract and methods that the trial participants were treated in the outpatient/community setting (hence their population is classed as a non severe patient group).

Thank-you – we have added “in ambulatory outpatients” in the first sentence of the Methods section. The abstract already stated that the trial was performed in outpatients but we have clarified that they were ambulatory.

The viral Laos was measured in daily saliva samples. Is there sufficient data to show how it correlates in terms of viral load with nasopharyngeal samples.

Many papers address this issue and we have added the following comment in the Discussion: “In other reports, viral load has been reported to be either higher [33] or lower [34] in saliva compared to nasopharyngeal swab but generally concordance is very good [33-35].” The early modelling paper used to inform FLARE [4] included viral trajectories in both saliva and NPS.

# 33. Beyene GT, Alemu F, Kebede ES, Alemayehu DH, Seyoum T, Tefera DA, *et al*. Saliva is superior over nasopharyngeal swab for detecting SARS-CoV2 in COVID-19 patients. *Sci Rep* 2021; 11:22640

# 34. Ursic T, Kogoj R, Sikonja J, Roskaric D, Virant MJ, Bogovic P, et al. Performance of nasopharyngeal swab and saliva in detecting Delta and Omicron SARS-CoV-2 variants. *J Med Virol* 2022; doi: 10.1002/jmv.27898.

# 35. Herrera LA, Hidalgo-Miranda A, Reynoso-Noveron N, Meneses-Garcia AA, Mendoza-Vargas A, Reyes-Grajeda JP, et al. Saliva is a reliable and accessible source for the detection of SARS-CoV-2. *Int J Infect Dis* 2021; 105:83-90.

# 4. Gastine S, Pang J, Boshier FAT, Carter SJ, Lonsdale DO, Cortina-Borja M*, et al.* Systematic Review and Patient-Level Meta-Analysis of SARS-CoV-2 Viral Dynamics to Model Response to Antiviral Therapies. *Clin Pharmacol Ther* 2021,110:321-333.

Statistical analyses

The study is inadequately powered but at the time of setting up the study in the early phase of the pandemic there was insufficient information and the authors have made reasonable attempts at estimating effect size.

We designed the trial at the start of the pandemic when there were limited data available and hence it was challenging to estimate the sample size parameters. The observed overall standard deviation is around 1.8 log10 and thus the variation is somewhat greater than that estimated. However, the attrition proportion on the trial is lower than we made allowance for. Overall, the observed treatment effect is smaller than the effect that we intended to detect, and there was no evidence of an interaction effect from the data. The study was powered to detect a particular effect which we anticipated to be clinically meaningful. A larger study may have detected a smaller effect on viral load but we did not consider this to be clinically worthwhile.

Results
Dominant population that has been recruited was under the age of 55 years with average age of 40 years. Hence, in this healthy population the placebo groups may have also more effectively cleared the virus and hence reducing the difference between the treatment and placebo group. Furthermore, almost 50% of patients recruited had received 2 doses of the vaccine. This may have further reduced the effective difference in viral clearance between the treat,ent and placebo groups. Have the authors factored age and vaccination into their linear mixed model.

We agree with the reviewer and indeed noticed an effect of vaccination (Supplementary Figure 5). Both age and vaccination status (and antibody status) were included in the model. Note that age was treated as a categorical variable (≤ 55 vs > 55 years) as we did not anticipate the effect to be linear.

It is very likely that a lot of the adjustments are post hoc and the authors need to be explicit in which factors were planned a priori and which factors were post hoc eg effect of delta variant, effect of vaccination if considered.

The only variable included post-hoc was baseline viral load. The inclusion of the Statistical Analysis Plan should help to clarify this. The mixed model analysis was also performed post-hoc but using the same predictor variables. This is specified in the text (see response to Reviewer 2 above).

Do the authors have an explanation why only 50% of patients had drug levels measured?

This was due to three main reasons. First, as specified in the protocol, when the Day 7 visit would have fallen on a Sunday (i.e. participant recruited on a Monday), this visit was usually performed on Day 8. As participants had completed their medication on Day 7, blood samples for pharmacokinetics could not be taken. Other aspects of the trial were unaffected (saliva samples in RNA preservation buffer are stable for up to 3 months at room temperature).

Second, some participants had discontinued medication before Day 7 due to side effects or personal choice and we could therefore not collect meaningful pharmacokinetic data on them.

Finally, some of the samples were unfortunately lost by a courier *en route* to Japan for analysis. Despite exhaustive searches, these could not be located.

We have not added specific text to explain these factors but would be happy to do so, according to editorial direction.

Viral pcr: did favipiravir have any effect on the viral sequence on day 5 compared to the other therapies. As favipiravir is a ribosomal-dependent RNA polymerase (RdRp) inhibitor we would anticipate multiple mutations in the virus. The authors mention they are performing viral sequencing and that data would significantly strengthen this study.

We thank the reviewer for raising this point which is very important. Sequencing of the samples is ongoing. Sequencing results also need to be interpreted in the context of pharmacokinetics and viral dynamics but this analysis will be reported separately.

**Reviewer #4:**

Overall summary: This phase 2 proof of principle, placebo controlled RCTwith 2x2 factorial double blind trial of OP with early COVID-19  from 2 sites in England using favipiravir, favipiravir and LPV-ritonavir, favipiravir plus LPV-ritonavir placebo, and both placebo using SARS-CoV2 VL at day 5 obtained through saliva as an outcome was safe, but with frequent side effects, most notably GI. There was no clinically significantly reduced VL in the primary analysis of the study drug.

Main strengths: The analysis was done during the pandemic when there were substantial challenges in patient recruitment, research staffing and changes in standards of care. It was also very difficult to bring outpatients in for research studies in a safe way due to limitations on transportation and health care facility rules. The authors should be commended for conducting an outpatient trial during this time period due to these substantial barriers.

Potential points to further explore:
It is unclear why patients who were asymptomatic were included in the trial, as they would be less likely to show an effect (ie they likely had lower viral loads to begin with) do the results change by excluding those?

We thank the reviewer for their generous comments about our trial. We accept that the inclusion of asymptomatic patients raised a potential issue as we could not define time since infection. This was why we only allowed a short timeframe for recruitment. However, only one patient was included under this criterion: we have clarified this in the manuscript. Their baseline viral load was 5.4 log10 which was above the mean.

In the demographics table, the authors indicated the number of doses received of the vaccine. It might be better to classify whether the participants who were vaccinated, were fully vaccinated or partially vaccinated (ie were they more than 14 days out from their most recent/final vaccination)? How did some patient receive 3 vaccines?

We considered this issue when the Steering Committee approved the inclusion of vaccinated participants. We do not believe there is a clear definition of ‘fully vaccinated’ in terms of number of vaccines and duration since vaccination. As the reviewer points out, the number of vaccinations varied in our study between 1 and 3 and we felt the fairest presentation of the data was to clearly state the number of doses received.

In the minimization table participants are labelled as vaccinated or not. Would redo this analysis with fully/partially/not vaccinated.

We agree that this would be an interesting analysis. However, this was not pre-specified in the Statistical Analysis Plan and we are reluctant to add too many post-hoc analyses. Furthermore, the biological justification in this particular cohort is less clear as participants had generally mounted a robust antibody response even to one vaccination (and antibody status was also included as a variable in the model).

The authors note that there was no significant reduction in VL in the primary analysis. The difficulty with the analysis is that the samples obtained were in the saliva, which is more convenient, but less likely to show an effect than a nasal swab. This is noted in the limitations, but significantly impacts the results of the study.

We have addressed this issue in our response above to Reviewer 3 and acknowledge the potential limitation of saliva samples in the Discussion.

A notable issue is the significant number of withdrawals/discontinuations of the groups taking LPV-r due to GI side effects, and the substantial drug-drug interactions. Appropriately the authors discuss the issues with using ritonavir for boosting; however this is being done fairly successfully with nirmatrelvir in 2022. Do the authors have any speculation for the differences between drop out rates for paxlovid real world data and for the LPV-r arms in this trial?

We thank the reviewer for raising this interesting point. Potential explanations might include:

1.) Lopinavir may have more side effects than nirmatrelvir.
2.) Our cohort was relatively young and healthy and may have been more likely to discontinue treatment as their capacity to benefit clinically was lower than in a high-risk group.
3.) We administered medications four times daily to maximise the predicted time >EC90 and this might have been a more difficult regime to adhere to than twice daily Paxlovid.

However, potential drug interactions remain a significant issue with Paxlovid and are similar to those with lopinavir-ritonavir.

We have added a comment into the Discussion as follows:

“Fortunately, data from trials so far have not indicated a high rate of discontinuation with nirmatrelvir-ritonavir [5]. Possible explanations include fewer side effects with nirmatrelvir compared to lopinavir, improved adherence among a high-risk group with more potential for clinical benefit and better adherence to a twice daily regime. However, this will need to be monitored in ‘real world’ settings.”

As noted in the limitations, this study population was young and very healthy. Would specifically note that there were few minorities, low rates of comorbidities and obesity, and over half were vaccinated, which could have affected the study effect. And make it less representative of those most affected by the COVID-19 pandemic. As such, would note this in the limitations. The authors do not report any data on resolution of symptoms other than duration of fever. It would be helpful to identify what symptoms patients had at the start of the trial and at the end to identify how sick they were. Presumably none of the patients were hospitalized, but that should be explicitly noted.

We agree with the reviewer on this point. This was already in the limitations but we have expanded as follows:

“The recruited cohort was relatively young and healthy with lower rates of comorbidities and obesity than often seen in the hospitalised population with COVID-19. Approximately 18% of participants were of non-white ethnicity which is similar to the UK population but may be less reflective of those with poor outcome. However, the trial was designed predominantly to look at viral load rather than clinical outcomes and therefore these factors were less important.”

We have also now included baseline symptom data in Table 1. None of the patients were hospitalized at baseline, as explained and clarified above in the response to Reviewer 3.
