# Supplementary material for: Favipiravir, lopinavir-ritonavir, or combination therapy (FLARE): A randomised, double-blind, 2 × 2 factorial placebo-controlled trial of early antiviral therapy in COVID-19
Source: PLoS Med. 2022 Oct 19;19(10):e1004120. doi: 10.1371/journal.pmed.1004120 (PMC9629589; doi:10.1371/journal.pmed.1004120)
Supplement: Attachment — Submitted filename: Response to Reviewers 300922.docx [file pmed.1004120.s019.docx]

**Response to editorial and reviewer comments**

**PMEDICINE-D-22-01160R2; Favipiravir, lopinavir-ritonavir or combination therapy (FLARE): a randomised, double blind, 2x2 factorial placebo-controlled trial of early antiviral therapy in COVID-19**

Requests from Editors:

GENERAL
The Data Availability Statement (DAS) requires revision. For each data source used in your study:
a) If the data are freely or publicly available, note this and state the location of the data: within the paper, in Supporting Information files, or in a public repository (include the DOI or accession number).
b) If the data are owned by a third party but freely available upon request, please note this and state the owner of the data set and contact information for data requests (web or email address). Note that a study author cannot be the contact person for the data.
c) If the data are not freely available, please describe briefly the ethical, legal, or contractual restriction that prevents you from sharing it. Please also include an appropriate contact (web or email address) for inquiries (again, this cannot be a study author).

We have adjusted the Data Availability Statement to include the contact details of the director of the UCL CCTU who can be contacted for inquiries.

In the first 1/3-1/2 of the manuscript you use square brackets to sign post tables and/or figures etc, in the latter part circular brackets.  Suggest the use of circular brackets throughout reserving square brackets for in-text reference call-outs and for however duplication of brackets are necessary)

Thank-you. We have changed square brackets to circular brackets for signpost tables and figures as suggested.

AUTHOR SUMMARY
Thank you for including an author summary I have made some suggestions for modifications as below, as we understand things (please note the requirement for bullet points):
Why was this study done?
   * The FLARE trial aimed to discover whether existing oral antiviral drugs could reduce the viral load of the SARS-CoV-2 virus if given soon after symptoms started.
   * If effective this strategy could reduce the risk of hospitalisation and death from COVID-19.

What did the researchers do and find?
   * The researchers performed a clinical trial of two medications - favipiravir and lopinavir/ritonavir, testing them on their own and in combination.
   * Combination therapies were less effective than favipiravir monotherapy, but many people taking lopinavir/ritonavir had gastrointestinal side effects and favipiravir drug levels were lower in the combination arm, possibly due to poor absorption.
   * SARS-CoV-2 viral loads were not significantly lower with any of the drug treatments after 5 days compared to placebo, although more people taking favipiravir had undetectable levels of the virus.

What do these findings mean?
   * None of these therapies should be used routinely at the current doses investigated.
   * Further studies investigating the effect of Favipiravir when administered at higher doses should be undertaken.

Many thanks – we are happy with this modified Author Summary and have included it in the manuscript.

METHODS and RESULTS
Please indicate explicitly in the main manuscript text that consent was written.

This is already included in the manuscript in two places: in the final paragraph of the ‘Study Design and Participants’ section and in the first paragraph of the ‘Study Procedures’ section.

TABLES
Table 3: please also provide unadjusted analyses

The ITT analysis in this table (i.e. the first line of data) is not adjusted for any baseline characteristics. The main model we used is an ANCOVA model, which is a regression between the value of viral load (VL) at follow-up against the baseline value and the treatment arm. The reason why it is necessary to include baseline viral load is that participants do not start the trial with the same VL. This is not an adjustment in the conventional sense of the term. Instead, it is part of the ANCOVA methodology. We have changed ‘adjusted for’ to ‘accounting for’ in the title of the Table to reduce any confusion.

FIGURES
Suppl figure 5: the graphs are very small thus rather inaccessible to the reader, please revise

Thank you for pointing this out. We have split Figure 5A and 5B onto separate pages so that the graphs are clearer.

Comments from Reviewers:

Reviewer #1: Alex McConnachie, Statistical Review

I thank the authors for their responses to my original comments. I am generally happy with these.

Seeing the SAP is good, but the paper does not signpost it when it is referred to in the methods section. I had hoped that the SAP would provide more information about the sample size calculation/simulation, but the text in the SAP is very much the same as the paper. Sufficient detail needs to be given to allow someone to replicate what was done (e.g. assumed treatment effects, and SD of the primary outcome).

Many thanks for the comment. We have signposted the SAP in the Methods section of the text.

You are correct that the missing part was the standard deviation. We have now added it in this revision of the manuscript. The SD of change for each group was assumed to be 1.3 log_10_ copies/mL.

My guess is that the sample size calculation for the main effects relates to a model without interaction, essentially treating the study as two separate trials, where the analysis in relation to one randomisation treats the other randomisation as a stratification factor; this is why I prefer that the non-interaction analysis is reported somewhere in the paper. Saying that, it is true that in this case, the interaction model is more informative, given the magnitude of the interaction term. Plus, it is possible to work out the results without the interaction, given the information presented, so seeing these results is more for convenience, than a requirement.

We can clarify that the sample size calculation includes an interaction term. Indeed, one of the main objectives of this trial was to identify a potential interaction between the two experimental treatments. The simulation used 2.5% alpha two-sided for the main effects, and 5% for the interaction term. We have added an additional supplementary appendix to this submission that contains the whole sample size simulation code in R.

Reviewer #2: I am satisfied that the authors have addressed the comments made on previous review. Well done on a valuable trial.

We thank the reviewer for their generous comment.
